# Exploring nucleo-cytoplasmic large DNA viruses in Tara Oceans microbial metagenomes

**DOI:** 10.1038/ismej.2013.59

**Published:** 2013-04-11

**Authors:** Pascal Hingamp, Nigel Grimsley, Silvia G Acinas, Camille Clerissi, Lucie Subirana, Julie Poulain, Isabel Ferrera, Hugo Sarmento, Emilie Villar, Gipsi Lima-Mendez, Karoline Faust, Shinichi Sunagawa, Jean-Michel Claverie, Hervé Moreau, Yves Desdevises, Peer Bork, Jeroen Raes, Colomban de Vargas, Eric Karsenti, Stefanie Kandels-Lewis, Olivier Jaillon, Fabrice Not, Stéphane Pesant, Patrick Wincker, Hiroyuki Ogata

**Affiliations:** 1 CNRS, Aix-Marseille Université, Laboratoire Information Génomique et Structurale (UMR 7256), Mediterranean Institute of Microbiology (FR 3479), Marseille, France; 2 CNRS and Université Pierre et Marie (Paris 06), UMR 7232, Observatoire Océanologique, Banyuls-sur-Mer, France; 3 Department of Marine Biology and Oceanography, Institute of Marine Science (ICM), CSIC, Passeig Marítim de la Barceloneta, Barcelona, Spain; 4 CEA, Institut de Génomique, Genoscope, Evry, France; 5 Department of Structural Biology, VIB, Brussel, Belgium; 6 Department of Applied Biological Sciences (DBIT), Vrije Universiteit Brussel, Brussels, Belgium; 7 European Molecular Biology Laboratory, Heidelberg, Germany; 8 CNRS, Université Pierre et Marie Curie (Paris 06), UMR 7144, Station Biologique de Roscoff, Roscoff, France; 9 MARUM—Center for Marine Environmental Sciences, Universität Bremen, Bremen, Germany; 10 Education Academy of Computational Life Sciences, Tokyo Institute of Technology, Tokyo, Japan

**Keywords:** eukaryotic viruses, marine NCLDVs, taxon co-occurrence, oomycetes

## Abstract

Nucleo-cytoplasmic large DNA viruses (NCLDVs) constitute a group of eukaryotic viruses that can have crucial ecological roles in the sea by accelerating the turnover of their unicellular hosts or by causing diseases in animals. To better characterize the diversity, abundance and biogeography of marine NCLDVs, we analyzed 17 metagenomes derived from microbial samples (0.2–1.6 μm size range) collected during the Tara Oceans Expedition. The sample set includes ecosystems under-represented in previous studies, such as the Arabian Sea oxygen minimum zone (OMZ) and Indian Ocean lagoons. By combining computationally derived relative abundance and direct prokaryote cell counts, the abundance of NCLDVs was found to be in the order of 10^4^–10^5^ genomes ml^−1^ for the samples from the photic zone and 10^2^–10^3^ genomes ml^−1^ for the OMZ. The Megaviridae and Phycodnaviridae dominated the NCLDV populations in the metagenomes, although most of the reads classified in these families showed large divergence from known viral genomes. Our taxon co-occurrence analysis revealed a potential association between viruses of the Megaviridae family and eukaryotes related to oomycetes. In support of this predicted association, we identified six cases of lateral gene transfer between Megaviridae and oomycetes. Our results suggest that marine NCLDVs probably outnumber eukaryotic organisms in the photic layer (per given water mass) and that metagenomic sequence analyses promise to shed new light on the biodiversity of marine viruses and their interactions with potential hosts.

## Introduction

Viruses are thought to be extremely abundant in the sea. Indeed, phages alone outnumber all other life forms in seawater, reflecting the abundance of their bacterial hosts (Suttle, 2007). However, little is known about the diversity, abundance and biogeography of marine viruses infecting other cellular organisms, in particular eukaryotes. Although less numerous than bacteria, eukaryotes often represent the bulk of plankton biomass and mediate important biogeochemical and food web processes (Falkowski *et al.*, 2004, Massana, 2011).

Nucleo-cytoplasmic large DNA viruses (NCLDVs; Iyer *et al.*, 2006, Yutin and Koonin, 2012) constitute an apparently monophyletic group of eukaryotic viruses with a large double-stranded DNA (dsDNA) genome ranging from 100 kb up to 1.26 Mb. Their hosts show a remarkably wide taxonomic spectrum from microscopic unicellular eukaryotes to larger animals, including humans. Certain NCLDVs are known to have important roles in marine ecosystems. For instance, *Heterosigma akashiwo* virus (HaV) affects the population dynamics of their unicellular algal host, which forms seasonal harmful blooms in coastal areas (Tomaru *et al.*, 2004). Another well-known virus (*Emiliania huxleyi* viruses (EhV)) controls the population of the ubiquitous haptophyte *E. huxleyi*, which can form vast oceanic blooms at temperate latitudes and exerts complex influence on the carbon cycle (Pagarete *et al.*, 2011). Other NCLDVs cause diseases in fishes and can lead to economic damages in aquaculture industries (Kurita and Nakajima, 2012). NCLDVs include viruses with very large virion particles, which do not pass through 0.2-μm filters typically used in viral metagenomics to separate free viruses from other organisms (Van Etten, 2011). The prototype of such large viruses, also referred to as giruses (Claverie *et al.*, 2006), is the amoeba-infecting *Acanthamoeba polyphaga* Mimivirus with a 0.75-μm virion particle and 1.18-Mb genome (Raoult *et al.*, 2004). Since the discovery of the giant Mimivirus from fresh water samples, NCLDVs have become a subject of broader interest. This has led to several conceptual breakthroughs in our understanding of the origin of viruses and their links to the evolution of cellular organisms (Claverie, 2006; Forterre, 2006; Raoult and Forterre, 2008; Forterre, 2010; Legendre *et al.*, 2012). The sequencing of the Mimivirus genome prompted the discovery of many close homologs in environmental sequence data (Lopez-Bueno *et al.*, 2009; Cantalupo *et al.*, 2011). Most notably, Mimivirus gene homologs were detected in the Global Ocean Sampling (GOS) marine metagenomes (Ghedin and Claverie, 2005; Monier *et al.*, 2008a; Williamson *et al.*, 2008), suggesting Mimivirus relatives exist in the sea. Soon afterwards, two giant viruses related to Mimivirus were isolated from marine environments. These are *Cafeteria roenbergensis* virus (CroV; 750 kb) infecting a major marine microflagellate grazer (Fischer *et al.*, 2010) and *Megavirus chilensis* (1.26 Mb) infecting *Acanthamoeba* (Arslan *et al.*, 2011). About 70 NCLDV genomes have been sequenced so far, of which about 15 represent marine viruses (Pruitt *et al.*, 2012). Thanks to this recent accumulation of sequence data and analyses, the visible portion of the NCLDV phylogenetic tree is fast expanding, and NCLDV abundance in the sea is increasingly being recognized. However, our knowledge of their biology is still limited, leaving such fundamental ecological parameters as their abundance and host taxonomic range to be determined.

Previous studies examined the abundance of specific species/groups of NCLDVs in marine environments using either laboratory culture of viral hosts or flow cytometry (FC). The concentration of HaVs infecting the raphidophyte *H. akashiwo* could reach 10^4^ viruses ml^−1^ in natural sea water during the period of host blooms (Tomaru *et al.*, 2004). The abundance of viruses (*Ostreococcus tauri* virus (OtVs)) infecting the smallest free-living green alga *O. tauri* could vary from undetectable levels to over 10^4^ viruses ml^−1^ depending on the season and the distance from the shore (Bellec *et al.*, 2010). The abundance of EhVs could reach over 10^7^ viruses ml^−1^ in rapidly expanding host populations in mesocosm experiments simulating host blooms (Schroeder *et al.*, 2003, Pagarete *et al.*, 2011). A typical observation in these studies was an episodic sudden increase (> several orders of magnitude) in virus concentration. These studies focused on specific viral species/strains and depended on the availability of host cultures for lysis evaluation or on relatively simple community compositions amenable to FC analysis. Currently, no direct method is available to assess the abundance of diverse NCLDVs in a complex microbial assemblage dominated by an overwhelming amount of bacterial cells and phages.

To better understand the diversity and geographical distribution of marine NCLDVs, we analyzed a subset of metagenomic sequence data (0.2–1.6 μm size fraction) generated by Tara Oceans, an international multidisciplinary scientific program aiming to characterize ocean plankton diversity, the role of these drifting microorganisms in marine ecosystems and their response to environmental changes (Karsenti *et al.*, 2011). Samples were collected during the first year of the expedition from the Strait of Gibraltar, through the Mediterranean and Red Sea, down to the middle of the Indian Ocean (Table 1). Some marine regions under-represented in previous metagenomic studies are included in this sample set, such as those from the Arabian Sea oxygen minimum zone (OMZ) and Indian Ocean lagoons. Most prokaryotic cells and many large virus particles are expected to be captured within the 0.2–1.6 μm size fraction used in the present metagenome study. Here we show that putative NCLDV sequences differ substantially from known reference genomes, suggesting a high diversity of giant marine viruses. The concentration of NCLDV genomes in the samples was estimated by factoring the metagenome data set with prokaryotic abundance determined by FC and microscopy on samples collected concurrently on Tara. Finally, we tested the capacity of the taxon co-occurrence patterns (Chaffron *et al.*, 2010, Steele *et al.*, 2011) present in our data set to provide hints about potential natural hosts for marine NCLDVs.

## Materials and methods

### Sampling and DNA extraction

At the end of March 2012, a 2.5-year circum-global expedition was completed onboard Tara, an arctic exploration schooner modified for global marine research with innovative systems for multiscale sampling of planktonic communities. During the expedition, planktonic organisms ranging in size from viruses to fish larvae together with physico-chemical contextual data were collected from several depths at 153 stations across the world oceans. Plankton were collected from up to three depths: near the surface (SRF; ∼5 m), at the depth of maximum chlorophyll *a* fluorescence (deep chlorophyll maximum, DCM; 20–200 m) and in the mesopelagic layer (MESO; 200–1000 m) to capture deep oceanographic features, such as OMZs. As much as possible where sampling was shallower than 80 m, SRF and DCM samples were collected using a large peristaltic pump (A40, TECH-POMPES, Sens, France), whereas samples from deeper DCM and MESO were collected using 12-l Niskin bottles mounted on a rosette equipped with physico-chemical sensors. For samples analyzed in this study, 100 liters of seawater from each depth were first passed through 200- and 20-μm mesh filters to remove larger plankton, then gently passed in series through 1.6- and 0.22-μm filters (142 mm, GF/A glass microfiber pre-filter, Whatman, Maidstone, UK; and 142 mm, 0.22 μm Express PLUS Membrane, Millipore, Billerica, MA, USA, respectively) using a peristaltic pump (Masterflex, EW-77410-10, Cole-Parmer International, Vernon Hills, IL, USA). The filters were kept for 1 month at −20 °C on board Tara and then at −80 °C in the laboratory until DNA extraction. DNA was extracted using a modified CTAB (hexadecyltrimethylammonium bromide) protocol (Winnepenninckx *et al.*, 1993): (i) the filters were incubated at 60 °C for 1 h in a CTAB buffer (2% CTAB; 100 mM TrisHCl (pH=8); 20 mM EDTA; 1.4 M NaCl; 0.2% β-mercaptoethanol; 0.1 mg ml^−1^ proteinase K; 10 mM DTT (dithiothreitol), (ii) DNA was purified using an equal volume of chloroform/isoamylalcohol (24:1) and a 1-h-long RNase digestion step, and (iii) DNA was precipitated with a 2/3 volume of isopropanol and washed with 1 ml of a EtOH/NH_4_Ac solution (76% and 10 mM, respectively). Finally, the extracted DNA samples were dissolved in 100 μl of laboratory grade water and stored at −20 °C until sequencing. On average, an approximate yield of 1 μg μl^−1^ was obtained for each sample.

### Metagenomic sequence data

All sequencing libraries were created using the Roche-454 Rapid Library kit (Roche Applied Science, Meylan, France). The input for nebulization used 500 ng of extracted DNA. Each library was indexed to avoid cross-contamination and sequenced on one-eighth to one-half of a GS-FLX Titanium plate (Meylan, France). Quality checking of the reads was performed using the 454 standard tools. 454-based pyrosequencing is known to generate artificial duplicates (Briggs *et al.*, 2007). Therefore, for each set of reads generated from the same sample by the same 454 run, we identified and removed artificial duplicates using the 454 Replicate Filter software (Gomez-Alvarez *et al.*, 2009) by applying the following criteria: ⩾5 identical starting nucleotides and ⩾97% overall nucleotide sequence identity. This resulted in an overall reduction of the number of reads by 16%, ranging from 3% to 47% depending on the sample. Metagenomic sequence data generated from Tara Oceans are referred to as Tara Oceans Project (TOP) metagenomes. The sequence data analyzed in this study is based on a subset of TOP metagenomes (Table 2), which is referred to as TOP pyrosequences or, in the present study, simply as TOP data. The sequence data are accessible from the Sequence Read Archive of the European Nucleotide Archive through the accession number ERA155562 and ERA155563. Additional sequence and annotation data are accessible from http://www.igs.cnrs-mrs.fr/TaraOceans.

The GOS metagenomic sequence reads (Rusch *et al.*, 2007) were downloaded from CAMERA (Sun *et al.*, 2011). We used only the sequence data recovered from the samples corresponding to the size fraction between 0.1 and 0.8 μm (that is, 40 samples corresponding to GS001 to GS051). Protein-coding regions in the metagenomic sequences (TOP and GOS) were identified using the FragGeneScan software (Rho *et al.*, 2010).

### Enumeration of prokaryotes by 4,6-diamidino-2-phenylindole (DAPI)

In all, 10 ml of seawater for SRF and DCM and 90 ml for OMZ (pre-filtered through 20-μm mesh) were fixed in paraformaldehyde (1.5% final concentration), filtered onto a 0.2-μm polycarbonate filter and kept frozen until processing. For the enumeration of total prokaryotes, cells were stained with DAPI and between 500 and 1000 DAPI-positive cells were counted manually in a minimum of 10 microscope fields using an Olympus BX51TF epifluorescence microscope (Olympus, Tokyo, Japan).

### Enumeration of prokaryotes by FC

For FC counts, three aliquots of 1 ml of seawater (pre-filtered through 200-μm mesh) were collected from each depth. Samples were fixed immediately using cold 25% glutaraldehyde (final concentration 0.125%), left in the dark for 10 min at room temperature, subsequently flash-frozen and kept in liquid nitrogen on board, and then stored at −80 °C in the laboratory. Two sub-samples were taken for separate counts of heterotrophic prokaryotes and phototrophic picoplankton. For heterotrophic prokaryote determination, 400 μl of sample was added to a diluted SYTO-13 (Molecular Probes Inc., Eugene, OR, USA) stock (10:1) at 2.5 μmol l^−1^ final concentration, left for about 10 min in the dark to complete the staining and run in the flow cytometer. We used a FacsCalibur (Becton and Dickinson, Franklin Lakes, NJ, USA) flow cytometer equipped with a 15-mW Argon-ion laser (488 nm emission). At least 30 000 events were acquired for each subsample (usually 90 000 events). Fluorescent beads (1 μm, Fluoresbrite carboxylate microspheres, Polysciences Inc., Warrington, PA, USA) were added at a known density as internal standards. The bead standard concentration was determined by epifluorescence microscopy. Heterotrophic prokaryotes were detected by their signature in a plot of side scatter vs FL1 (green fluorescence). In a red (FL3) –green (FL1) fluorescence plot, beads fall in one line, heterotrophic prokaryotes in another and noise in a third (respectively, with more FL3 than FL1). Picocyanobacteria fall in between noise and heterotrophic prokaryote. This method is based on del Giorgio *et al.* (1996) as discussed in Gasol and del Giorgio (2000). For phototrophic picoplankton, we used the same procedure as for heterotrophic prokaryote but without addition of SYTO-13. Small eukaryotic algae were identified in plots of side scatter vs FL3, and FL2 vs FL3 (Olson *et al.*, 1993), and excluded in the enumeration of phototrophic prokaryotes. Data analysis was performed with the Paint-A-Gate software (Becton and Dickinson). The abundance of prokaryotic cells was based on the enumerations of heterotrophic and phototrophic prokaryotes.

### NCLDV classification

Throughout this study, we used the NCLDV nomenclature derived from the common ancestor hypothesis (Iyer *et al.*, 2006) based on seven distantly related viral families: Megaviridae, Phycodnaviridae, Marseilleviridae, Iridoviridae, Ascoviridae, Asfarviridae and Poxviridae. Among theses, Megairidae is a recently proposed family (Arslan *et al.*, 2011), which includes Mimivirus, Mamavirus, Megavirus, CroV and other marine viruses such as *Pyramimonas orientalis* virus, *Phaeocystis pouchetii* virus (PpV), *Chrysochromulina ericina* virus (CeV) as well as Organic Lake Viruses (OLPV1, OLPV2) (Ogata *et al.*, 2011; Yau *et al.*, 2011). Although the order Megavirales was recently proposed to refer to the taxonomic classification of NCLDVs (Colson *et al.*, 2012), we simply refer here to these viruses collectively as NCLDVs.

### Marker genes

Sixteen NCLDV marker genes were selected from the 1445 clusters of NCLDV orthologs, represented in the NCVOG database (Yutin *et al.*, 2009). These marker genes were selected based on their conservation in nearly all known NCLDV genomes (four markers) or in a majority of viruses from the two major marine NCLDV families (Megaviridae and Phycodnaviridae; 12 markers), as well as on the observation that these genes typically occur only once in their genomes if present (Supplementary Table S1). For cellular organisms, we used 35 conserved genes normally encoded as a single copy in all the cellular organisms (Raes *et al.*, 2007). Profile-hidden Markov models (Eddy, 2008) derived from the sequence alignments of these marker genes were used to identify their homologs (*E*-value⩽10^−3^) in the translated amino-acid sequence sets derived from metagenomic data. After identification of the marker gene homologs, taxonomic assignment was performed using the dual BLAST based last common ancestor (2bLCA) method described below in order to separate these sequences in distinct NCLDV, Bacteria, Archaea and eukaryote bins. For each marker gene, we then obtained marker gene density in the metagenomes (number of hits per Mbp). A normalization process for the marker gene size was introduced by dividing the computed marker gene density by the length of the reference multiple sequence alignment of the profile-hidden Markov model.

### Phylogenetic mapping

Phylogenetic mapping (Monier *et al.*, 2008a) is a method to place and classify a new sequence (usually a short environmental sequence) within a reference tree using a precompiled multiple sequence alignment. In this study, we compiled a reference sequence set composed of 187 type B DNA polymerase (PolB) homologs and a reference sequence set composed of 154 MutS homologs from diverse cellular organisms and viruses (Supplementary Figures S1 and S2). Multiple sequence alignments and phylogenetic trees were constructed using T-Coffee (Notredame *et al.*, 2000) and RAXML (Rokas, 2011). HMMALIGN was used to align metagenomic sequences on the reference alignments and Pplacer (Matsen *et al.*, 2010) was used to map the sequences in the reference trees using the Bayesian option. This Pplacer approach was used also for the phylogenetic analysis of the reads assigned to the Megaviridae and oomycetes taxonomic nodes. For the visualization of phylogenetic trees, we used Archaeopteryx (Han and Zmasek, 2009), FigTree (http://tree.bio.ed.ac.uk/software/figtree/) and MEGA version 5.1 (Tamura *et al.*, 2011).

### 2bLCA taxonomic annotation

Each 454 read >100 bp in length was assigned a taxonomic classification using a dual BLAST (Altschul *et al.*, 1997; Monier *et al.*, 2008b) based last common ancestor (2bLCA) approach somewhat similar to the method applied by MEGAN (Huson *et al.*, 2007) but using an adaptive *E*-value threshold specific for each protein. For each 454 read, the best local alignment (high-scoring segment pair (HSP)) with known proteins was obtained by a first BLAST (B1; BLASTx) against the UniProt database release April 2011 (UniProt Consortium, 2012). Reads without any HSPs at an *E*-value⩽10^−5^ were classified as ‘no hits'. For each read with at least one significant HSP, the subsequence of the UniProt subject fragment aligned in the best scoring B1 HSP was used as a second BLAST (B2; BLASTp) query against the same UniProt database. All the B2 database hits with an *E*-value⩽B1 HSP were recorded and defined to constitute a set of close homologs for the read (denoted as set H). The taxonomic classifications (Benson *et al.*, 2012) of the set H were then reduced to their LCA, which was finally assigned to the read as its taxonomic annotation. Reads were annotated as ‘ambiguous' if the set H contained representatives from several domains of life. This 2bLCA protocol was applied to the metagenomic reads as well as to the metagenomic marker gene homologs (predicted protein sequences). For the latter case, we used BLASTp for B1 (instead of BLASTx) against a customized reference database (that is, a subset of UniProt) with enriched taxonomic annotations for NCLDVs. The use of two protein reference databases in this study merely reflects the period when the computation was performed.

### Read abundance per taxon

For each set of taxa at a given depth (here fifth level from the root) in the National Center for Biotechnology Information (NCBI) taxonomic tree of life, we estimated the relative read abundance of plankton representatives for each taxon in each Tara Oceans sample (providing a *samples* × *taxa* matrix). The relative read abundance of a specific taxon for a specific sample was calculated as the number of 454 metagenomic reads with a taxonomic annotation at or below the taxon level divided by the total number of 454 reads in the sample. The resulting matrix composed of 712 taxa (rows) across 17 samples (columns) is provided (Supplementary Files S1 and S2).

### Co-occurrence analysis

The 712 taxa × 17 samples matrix from above was first filtered to exclude taxa with <5 total reads, reducing the matrix to 609 taxa. To normalize the read counts with respect to varying sequencing depth across samples, the number of reads in each cell of the matrix was divided by the total number of reads for the corresponding column. In order to detect putative taxon co-occurrences across the 17 samples, rank-based Spearman correlation coefficients (*ρ*) were first computed between taxon pairs using the R ‘stats' package ‘cor' function (R Development Core Team, 2011). Significance of each *ρ* was tested by computing a two-sided *P*-value (asymptotic *t* approximation) using the R ‘stats' package ‘cor.test' function and controlled for multiple tests using false discovery rate (*q*-value) computed by the tail area-based method of the R ‘fdrtool' package (Strimmer, 2008). Taxon associations with |*ρ*|>0.7 and *q*<0.05 were reported with this first approach. Taxon co-occurrences/co-exclusions were also independently assessed by the method described by Faust *et al.* (2012). In this second and more stringent approach, the two samples from OMZ were excluded to reduce the detection of biome-specific patterns in species distributions. In addition, we excluded parent–child taxonomic relationships (for example, an association between ‘Viruses' and ‘Phycodnaviridae') in this second analysis. Briefly, taxon associations were measured with Spearman's correlation (denoted as *ρ*') and Kullback–Leibler distance on the input matrix. The 1000 top- and 1000 bottom- ranking edges for each method were further evaluated according to Faust *et al.* (2012), which mitigates biases introduced by data normalization. This method builds a null distribution of scores for each edge by permuting the corresponding taxon rows while keeping the rest of the matrix unchanged and then restores the compositional bias by renormalizing the matrix. We ran 1000 rounds of permutation-renormalization for each edge and 1000 bootstraps of the matrix columns to calculate the confidence intervals around the edge score. The *P*-value for each measure was obtained from the *Z*-scores of the permuted null and bootstrap confidence interval; they were combined (denoted as *P*'-values) using a method conceived for non-independent tests (Brown, 1975) and corrected for multiple testing using false discovery rate *q*-values (denoted as *q*'-values) according to Benjamini and Hochberg (1995). Taxon associations with *q*'<0.05 were reported with this second approach.

### Horizontal gene transfer (HGT) analysis

To identify potential HGTs between Megaviridae and oomycetes, comprehensive proteome databases for each taxon were assembled as follows. The Megaviridae proteome database contained all 6678 publically available peptides for *M. chilensis* (1120 peptides), Megavirus courdo7 (1139 peptides), *Acanthamoeba castellanii* mamavirus (997 peptides), *A. polyphaga* mimivirus (972 peptides), *A. polyphaga* mimivirus isolate M4 (756 peptides), Moumouvirus Monve (1150 peptides) and CroV BV-PW1 (544 peptides). Because complete oomycete proteomes were poorly represented in the UniRef100 database release December 2010 (Suzek *et al.*, 2007) which we intended to use for HGT detection, we enriched UniRef100 with oomycete proteomes from the following publically available oomycete genome and transcriptome projects (Supplementary Table S2): *Aphanomyces euteiches* ESTs (161 384 open reading frames (ORFs)) (Gaulin *et al.*, 2008), *Hyaloperonospora arabidopsidis* (14 937 ORFs) (Baxter *et al.*, 2010), *Pythium ultimum* (14 224 peptides) (Levesque *et al.*, 2010), as well as *Hyaloperonospora parasitica* (6452 peptides), *Phytophthora infestans* (14 580 peptides), *Phytophthora ramorum* (10 892 peptides), *Phytophthora sojae* (13 995 peptides) and *Saprolegnia parasitica* (17 437 peptides) available from the Broad Institute of Harvard and MIT ‘Saprolegnia and Phytophthora Sequencing Project'. Where peptides were not made available, nucleotide sequences were translated into ORFs >50 amino acids. To these 265 433 non-redundant oomycete peptides, we added a none-oomycete stramenopile proteome from *Thalassiosira pseudonana* (11 532 peptides), absent from UniRef100 but publically available at the NCBI. The 386 000 additional stramenopile peptides were clustered (90% identity, 265 433 peptides) before concatenation with UniRef100 to form the ‘UniRef100+stramenopiles' database.

Potential HGTs between Megaviridae and cellular proteins were first approximated by reciprocal best BLAST hits computed by a method similar to the one described by Ogata *et al.* (2006). Briefly, the best cellular homolog in the UniRef100+stramenopiles database was first identified for each Megaviridae peptide (BLASTp, *E*-value⩽10^−5^). If this best cellular homolog obtained a best hit against a Megaviridae peptide in a second BLASTp search against the UniRef100+stramenopiles+Megaviridae database (excluding hits in the same cellular taxonomic group at the first three NCBI classification levels), they were considered a potential Megaviridae-cell HGT candidate.

The six Megaviridae-oomycete HGT candidates revealed by reciprocal BLAST were then subjected to phylogenetic analysis. Homologs for the six Megaviridae peptides were collected by keeping representative sequences among all detected taxonomic groups using BLAST-EXPLORER (Dereeper *et al.*, 2010). Alignments were built using MUSCLE (Edgar, 2004) and GBLOCKS (Talavera and Castresana, 2007) except for the following two cases. For the putative fucosyltransferase AEJ34901, we used MAFFT/l-INS-i method (Katoh *et al.*, 2005). For the putative RNA methylase gi|311977703, we used CLUSTALW (Chenna *et al.*, 2003) followed by manual curation of the alignment. For these two cases, all alignment positions with >45% gaps were removed before phylogenetic analysis. Phylogenetic trees were inferred using PhyML (Guindon and Gascuel, 2003) implemented in Phylogeny.fr (Dereeper *et al.*, 2008) with 100 bootstrap replicates. The generated trees were mid-point rooted.

## Results

### General features of the metagenomes

Samples in this study were collected as part of the Tara Oceans expedition between 13 September 2009 and 23 April 2010. The 17 microbial samples analyzed are from the 13 sampling sites and correspond to the size fraction between 0.2 and 1.6 μm (Table 1). These samples were selected to represent a broad range of biomes. Direct sequencing of extracted DNA by the GS-FLX Titanium 454 pyrosequencing technology yielded 2.8 billion bp (8 million reads; Table 2), which correspond to >40% of the size of sequence data in total base pairs produced by the previous GOS survey (Rusch *et al.*, 2007). Average G+C % varied from 37% to 48% across samples, and 8  358  544 ORFs (102 aa in average) were identified. These constitute the TOP data set analyzed in this study.

### Abundance of NCLDVs

**Figure 1:**
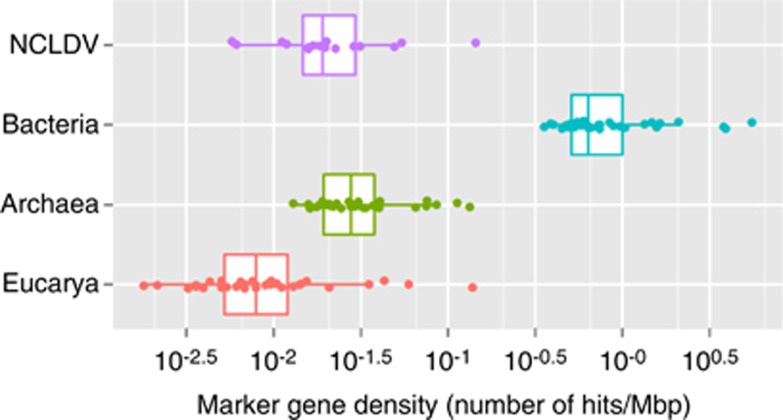
Metagenome-based relative abundance of NCLDV and cellular genomes in the TOP data set. Seventeen TOP metagenomes (0.2–1.6 μm size fraction) were pooled and analyzed as a single data set to generate this plot. Each dot in the plot represents the density of one of the marker genes used in this study (16 markers for NCLDVs and 35 markers for cellular genomes). The estimated abundance of NCLDVs genomes is slightly lower than that of Archaea genomes and amounts to approximately 3% of bacterial genomes.

We used 16 NCLDV marker genes and 35 cellular marker genes to assess the abundance of genomes represented in the metagenomic data. These markers are usually encoded as single copy genes in their genomes, therefore their abundance in metagenomes reflects the number of (haploid) genomes in the sequenced samples. The median density (hits per Mbp) of the NCLDV marker genes in our whole metagenomic data set was found to be 0.019 (Figure 1), which is lower than the marker gene density for Archaea (0.028) and corresponds to 3% of the density for Bacteria (0.64). The median density of the marker genes for eukaryotes was about half that of NCLDVs (0.008). The same method applied to the GOS marine metagenomic data, recovered from microbial samples (0.1–0.8 μm size fraction) collected along a transect from the North Atlantic to the Eastern Tropical Pacific, revealed that the marker gene density of NCLDVs (0.05) was as high as 10% of Bacteria (0.47) (Supplementary Figure S3). This ratio is higher than that for TOP samples likely reflecting the exclusion of large bacterial cells and the inclusion of small NCLDVs in the GOS 0.1–0.8 μm size fraction.

**Figure 2:**
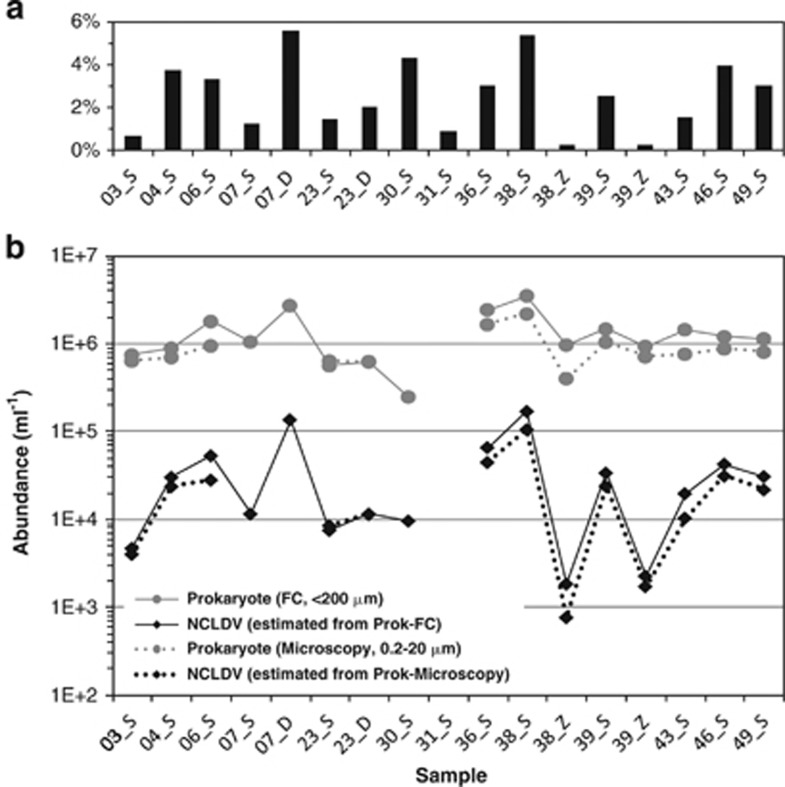
NCLDV genome abundance in the TOP data set. (**a**) Proportion of the average marker gene density for NCLDVs relative to that of prokaryotes (Bacteria and Archaea) for each of the 17 TOP metagenomes. (**b**) Experimentally measured prokaryotic cell densities (gray circles; 16 samples by microscopy and 13 samples by FC) were used to estimate the absolute abundances of NCLDV genomes (black squares) by rescaling the metagenome-based relative abundances. ‘S', ‘D' and ‘Z' in the sample names indicate the depths from which the samples were collected: ‘S' for surface, ‘D' for deep chlorophyll max and ‘Z' for oxygen minimum zone.

The computed abundance of NCLDV genomes relative to prokaryotic genomes varied from 0.2% to 5.6% across the 17 Tara samples (Figure 2a). We used prokaryotic cell abundances measured by FC and microscopy on water samples collected onboard Tara concomitantly with the metagenome samples, to re-scale the relative NCLDV genome abundance into absolute concentrations. FC analysis performed on 16 water samples (<200 μm size fraction) showed that prokaryotic cell density varied from 2.5 × 10^5^ to 3.5 × 10^6^ cells ml^−1^ (Figure 2b). Direct cell count by microscopic analysis for 13 samples (0.2–20 μm size fraction) provided comparable measures varying from 4.0 × 10^5^ to 2.2 × 10^6^ cells ml^−1^. We observed no algal bloom during our sampling, and these measures fall within typical ranges of prokaryotic cell density in the oceans (Suttle, 2005). We used GF/A pre-filters (glass microfiber, 1.6 μm nominal pore size) to collect samples for the present metagenomic sequencing as previous works indicate that the vast majority of prokaryotic cells (90–94%) pass through GF/A filters (Lambert *et al.*, 1993; Massana *et al.*, 1998). By assuming that 90% of prokaryotic cells observed by FC (<200 μm) or microscopy (0.2–20 μm) could pass through the 1.6-μm GF/A pre-filters, the absolute abundance of NCLDV genomes ml^−1^ of sea water in the 0.2–1.6 μm size fraction was estimated (Figure 2b). The NCLDV genome abundance was found to vary from 4 × 10^3^ to 1.7 × 10^5^ ml^−1^ with an average of 4.5 × 10^4^ genomes ml^−1^ for samples from photic zones (SRF and DCM). Samples from OMZ showed reduced NCLDV abundances (7.7 × 10^2^–2.3 × 10^3^ NCLDV genomes ml^−1^).

The detection of homologous sequences by a marker gene depends on numerous factors such as its level of conservation and gene length, as well as the taxonomic composition of the metagenomes being analyzed. We presumed that the use of multiple genes with largely different enzymatic functions would increase the overall accuracy of our procedure. To estimate the effect of possible artifacts, we repeated the above calculations after adding marker gene size normalization. This reduced the abundance estimates of NCLDV genomes by 38% compared with calculations without gene size normalization (Supplementary Figure S4).

### Megaviridae and prasinoviruses are the most abundant group of NCLDVs

**Figure 3:**
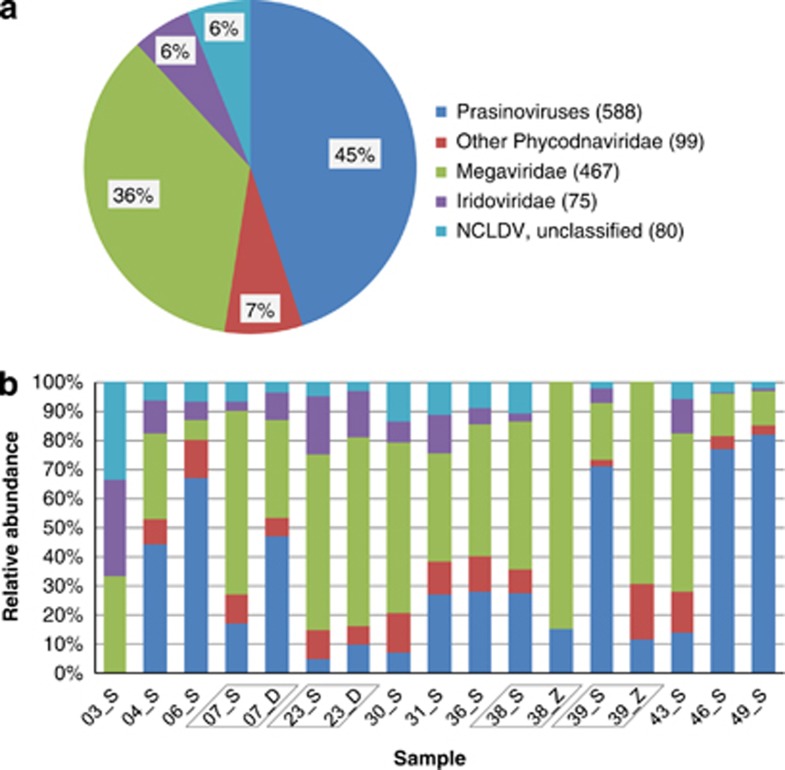
Metagenome-based relative abundance of NCLDV families. (**a**) Representation of different viral groups in the whole TOP metagenomic data set as measured by the NCLDV marker gene density. The number of marker reads taxonomically assigned to each viral group is shown in parentheses in the legend. (**b**) Representation of different viral groups in the 17 TOP metagenomic samples. ‘S', ‘D' and ‘Z' in the sample names indicate the depths from which the samples were collected: ‘S' for surface, ‘D' for deep chlorophyll max and ‘Z' for oxygen minimum zone. In both (**a**) and (**b**), three reads and one read assigned to Asfarviridae and Poxviridae, respectively, were omitted for presentation purpose.

In total, we identified 1309 NCLDV marker gene homologs in the TOP metagenomes. Our BLAST-based taxonomic annotation (see Materials and methods) revealed two dominant NCLDV families (Figure 3). Over half (52%) of them were attributable to the Phycodnaviridae family, while 36% were most closely related to the Megaviridae family. These two families together represented nearly 90% of the detected NCLDV marker gene sequences. This result confirmed a previous observation on the relative abundance of these two families among NCLDVs in a survey of the GOS data set (Monier *et al.*, 2008a). At the same sampling locations (stations 7 and 23), prasinoviruses (infecting green algae of the Mamiellophyceae class) were found to be relatively more abundant in DCM than in SRF samples (2.4–8.3-folds in absolute abundance), consistent with the photosynthetic activity of their hosts. No other notable difference in the virus family patterns was observed across depths (SRF, DCM, OMZ for stations 7, 23, 38, 39).

**Figure 4:**
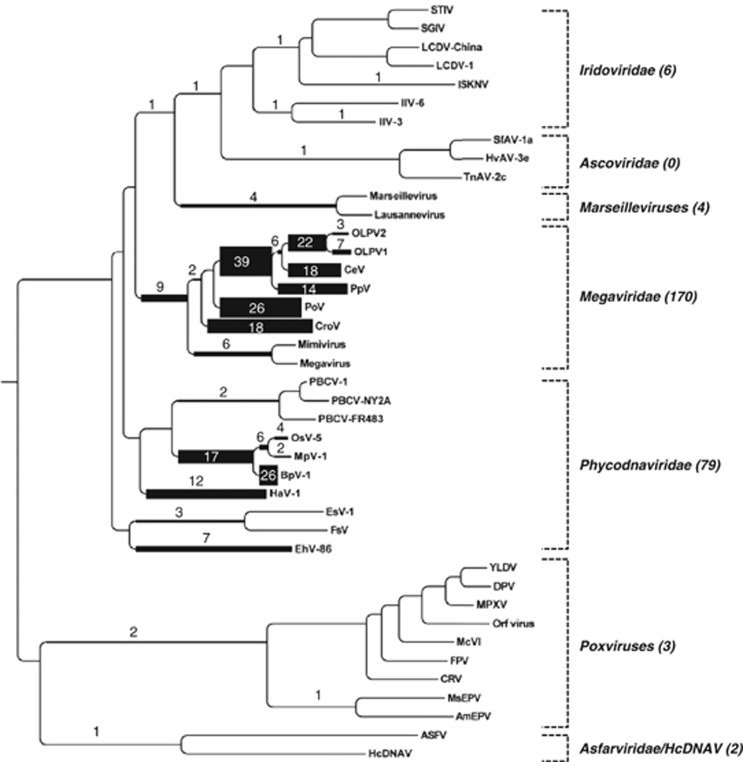
Phylogenetic positions of metagenomic reads closely related to NCLDV DNA polymerase sequences. An HMM search with a PolB profile detected 2028 PolB-like peptide sequences in the TOP metagenomes. Each of these peptides was placed within a large reference phylogenetic tree containing diverse viral and cellular homologs (Supplementary Figure S1) with the use of Pplacer. Of these peptides, 264 were mapped on the branches leading to NCLDV sequences and are shown in this figure. The numbers of mapped metagenomic reads are shown on the branches and are reflected by branch widths. This result is consistent with the preponderance of the Phycodnaviridae and Megaviridae families seen in our BLAST-based marker gene analysis. Only the NCLDV part of the reference tree is shown.

**Figure 5:**
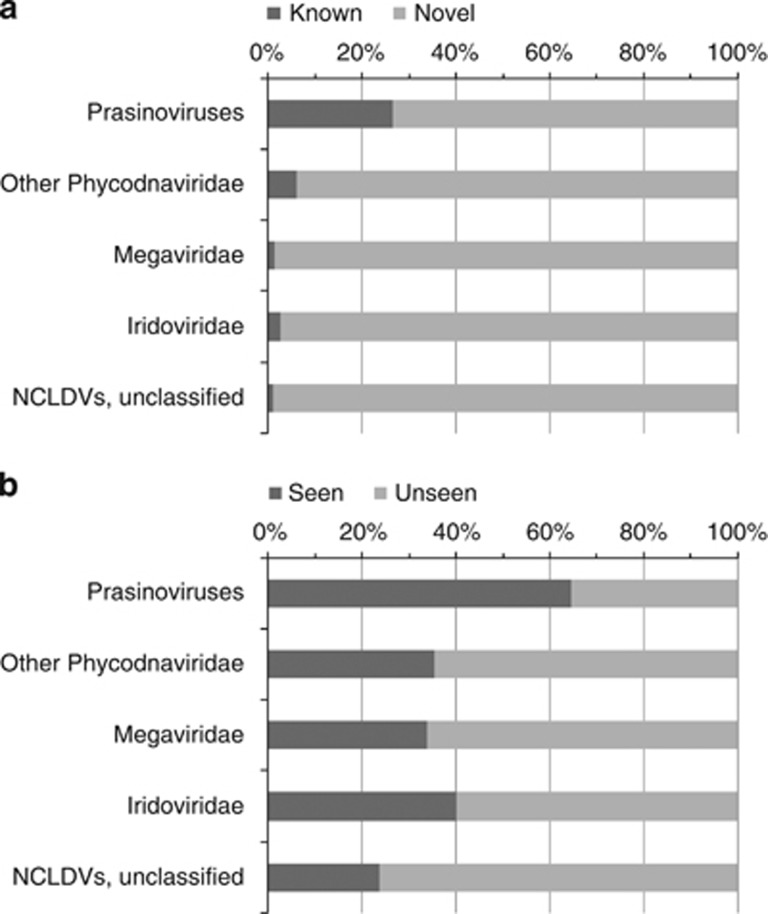
Classification of NCLDV marker genes in the TOP data based on the level of sequence similarity to database sequences. Metagenomic reads showing ⩾80% amino-acid sequence identity to database sequences were classified as ‘known (or seen)', otherwise as ‘novel (or unseen)'. (**a**) BLAST result against UniProt. (**b**) BLAST result against the GOS data. The large proportions of ‘novel (and unseen)' genes suggest current environmental surveys are far from reaching saturation and that diverse yet unknown NCLDVs exist in the sea.

An independent classification using PolB phylogenetic mapping analysis showed a globally similar taxonomic distribution of reads across different NCLDV lineages (Figure 4). Thanks to the recent expansion of available reference genomic sequences for Phycodnaviridae and Megaviridae families, prasinoviruses can now clearly be recognized as the most abundant group of marine phycodnaviruses. Within the Megaviridae branches, the two largest amoeba-infecting viruses (Mimivirus and Megavirus) are rather under-represented (3.5% of Megaviridae), while most reads were assigned to other Megaviridae branches, leading to viruses characterized by reduced genomes (from ∼300 to 730 kb). The hosts of the latter viruses are distributed widely in the classification of eukaryotes: *C. roenbergensis* (stramenopiles; Bicosoecida), *P. orientalis* (Viridiplantae; Chlorophyta; Prasinophyceae), *P. pouchetii* (Haptophyceae; Phaeocystales) and *Haptolina ericina* (formerly *C. ericina*; Haptophyceae; Prymnesiales). Interestingly, many metagenomic reads were assigned to relatively deep branches. For example, 17 PolB-like reads were assigned to the branch leading to the clade containing three prasinoviruses (OsV5, MpV1, BpV1), and 39 PolB-like reads were assigned to the basal branch leading to four marine viruses (PpV, CeV, OLPV1 and OLPV2). To illustrate metagenome sequence divergence with known viral sequences, we arbitrary classified the metagenomic NCLDV marker sequences as ‘known' if they showed ⩾80% amino-acid sequence identity to their closest homolog in the databases and otherwise as ‘novel' (or ‘unseen'). A vast majority (73–99%) of the sequences turned out to be ‘novel' when they were searched against the UniProt sequence database (Figure 5). Similarly, searches against the GOS sequence database revealed that large proportions (36–76%) of the TOP marker gene homologs were ‘unseen' in this previous large-scale marine microbial survey. A fragment recruitment plot for the OLPV1 PolB protein sequence applied to PolB-like metagenomic reads that best matched OLPVs (OLPV1 or OLPV2) further showed a high level of richness among these sequences (even within a single sample) and their large divergence from the reference OLPV1 sequence (Supplementary Figure S5). Overall, these results suggest that the majority of the NCLDVs represented in the TOP samples are highly diverse and only distantly related to known viruses, thus potentially corresponding to viruses infecting different marine eukaryotes.

### Correlated abundance of MutS protein subfamilies with Megaviridae abundance

Two recently identified subfamilies of DNA mismatch repair protein MutS are specific to a set of viruses with large genomes (Ogata *et al.*, 2011). The MutS7 and/or MutS8 subfamilies are encoded in all the known members of the Megaviridae family and in HcDNAV (356 kb); the latter virus infects the bloom-forming dinoflagellate *Heterocapsa circularisquama* and appears to be related to the Asfarviridae family (Ogata *et al.*, 2009). It has been suggested that these hallmark genes of giant viruses are required to maintain the integrity of viral genomes with large sizes (mostly >500 kb; Ogata *et al.*, 2011). These MutS genes are not included in our NCLDV marker gene set. Prompted by the observed high abundance of sequences of possible Megaviridae origin in the TOP data set, we screened our data for MutS7 and MutS8 homologs. In total, we identified 78 reads similar to MutS (68 and 10 reads for MutS7 and MutS8, respectively) in 13 samples (Supplementary Figure S6a). If these MutS genes originate from putative Megaviridae viruses detected by our marker gene method, we expect to see a correlation in their abundance across samples. We tested this hypothesis and found a statistically significant correlation between the relative abundance of the Mut7/8 homologs and the Megaviridae marker gene density (*R*=0.725, *P*=9.90 × 10^−4^; Supplementary Figure S6b). A similar level of correlation was also found in the GOS data set (*R*=0.647; *P*=6.55 × 10^−6^; Supplementary Figure S6c). This result suggests that the TOP reads assigned to the Megaviridae family probably originate from viruses with a large genome as found in known viruses of this family.

### Oomycetes or their stramenopile relatives co-occur with marine Megaviridae

To test whether the present data set might serve to identify potential hosts of marine NCLDVs, we assessed association of taxon occurrences (‘co-occurences' and ‘co-exclusions') across samples using the whole set of the TOP metagenomic reads. We used two approaches for the detection of taxon associations: the first based on Spearman's correlation across all samples (3696 associations, *q*<0.05), and the second combining Spearman's correlation with a Kullback–Leibler measure of dissimilarity on a reduced data set excluding two outlier OMZ samples (108 associations, *q*'<0.05). This resulted in the identification of a total of 3703 potential taxon association pairs, of which 101 were supported by both methods (Supplementary Table S3). The discrepancy between the two lists was due to the higher intrinsic stringency of the second method, as well as to the specific photic-OMZ contrasts, which were only taken into account by the first method. Some of the inferred taxon associations simply reflected uncertainty in the taxonomic assignments, such as the associations between ‘Archaea; environmental samples' and ‘Archaea; Euryarchaeota; Marine Group II; environmental samples;' (*q*=1.38 × 10^−8^, *q*'≈0) or between environmental viruses and myoviruses (*q*=3.8 × 10^−5^, *q*'=9.4 × 10^−3^). These could be explained by the taxonomic assignments of similar organisms into related but distinct taxonomic nodes in the NCBI taxonomy database.

However, our analysis also revealed known biological associations of lineages. For instance, a correlated occurrence (*q*=1.33 × 10^−3^, *q*'=8.42 × 10^−7^) was detected between two distinct *Bacteroidetes* lineages (that is, *Sphingobacteria* and *Cytophagia*), which are known to co-exist in seawater likely being attached to phytoplankton cells (Gomez-Pereira *et al.*, 2012). We also observed known virus–host pairs, such as a T4-like phage/cyanobacteria association (*q*=9.7 × 10^−3^) and an association between unclassified phycodnaviruses (mostly prasinoviruses) and a group of environmental prasinophytes (*q*=0.014). An example of co-excluding taxa was a relationship between *Prochlorococcus*, existing in the euphotic zone, and sulfur-oxidizing symbionts, a lineage of γ-Proteobacteria known to have an important role in sulfur-oxidizing microbial communities in deeper aphotic OMZs (*q*=0.011; Canfield *et al.*, 2010; Stewart *et al.*, 2012). The latter case appeared to simply reflect their non-overlapping waters of residence. These known association examples served as controls, suggesting that the inferred network might be mined usefully for putative novel associations (or segregations) of plankton organisms.

**Figure 6:**
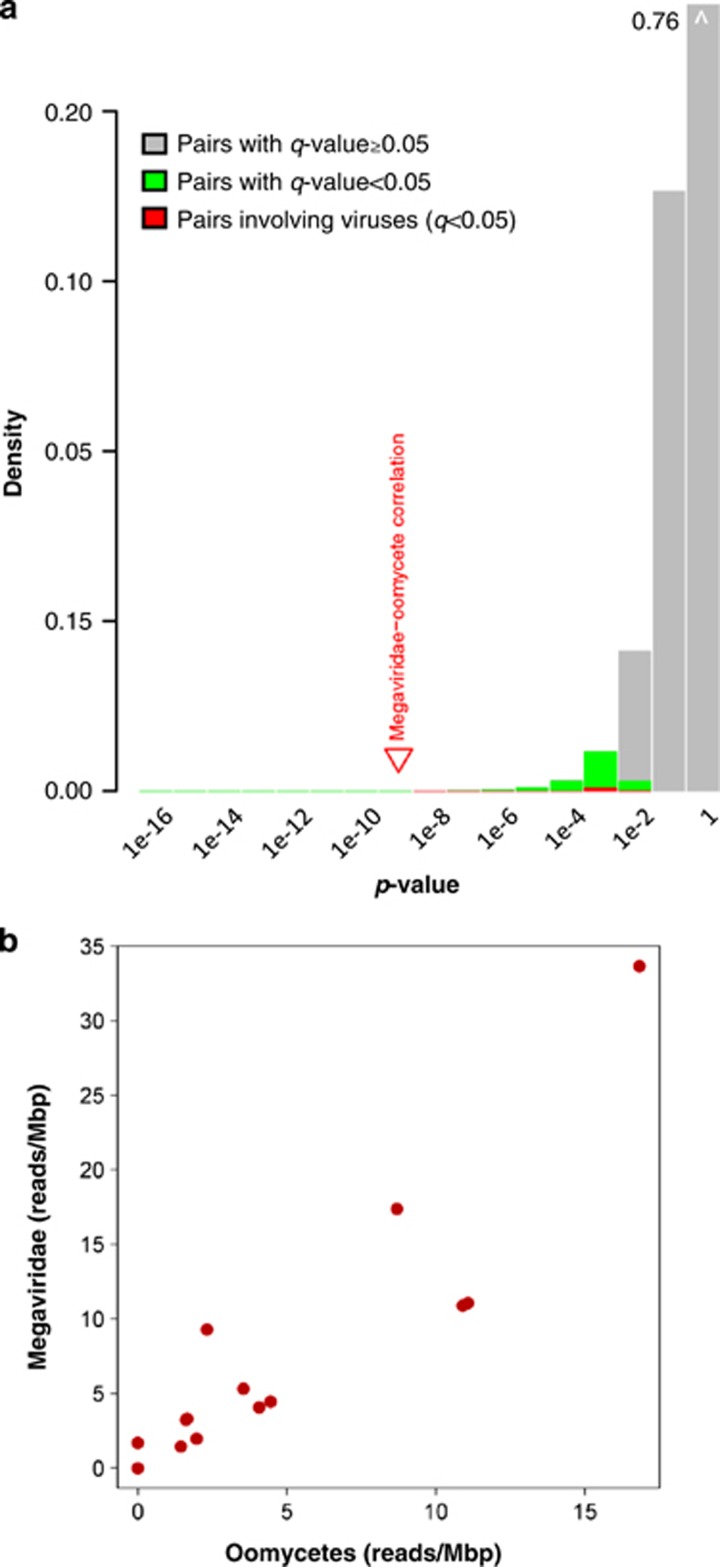
Taxon associations inferred from co-occurrence analysis. (**a**) Distribution of *P*-values for Spearman's correlation coefficients for taxon associations observed in the TOP metagenomic data. Colored (red and green) areas of the histogram represent taxon pairs showing statistically significant correlations. The position of the *P*-value for the hypothetical positive association between the ‘Megaviridae' and ‘oomycetes' taxonomic groups is indicated by a red triangle. (**b**) Correlated occurrence of 454 reads taxonomically assigned to the ‘Megaviridae' and the ‘oomycetes' groups by the BLAST-based 2bLCA method. Each dot corresponds to one of the 17 TOP samples analyzed. Axes represent the density of these reads (number of reads per Mbp) for each of the ‘Megaviridae' and the ‘oomycetes' groups.

Examples of positive and negative correlations between virus and cellular organism abundances are listed in Table 3. We have no simple explanation for some of the taxon pairs, such as the virus–cell mutual exclusions as well as the association of eukaryotic viruses with some bacteria (although the latter could be due to bacterial genes acquired by HGT in a viral genome). However, the association between the taxonomic node for ‘Megaviridae' (NCBI taxonomy: Viruses; dsDNA viruses, no RNA stage; Mimiviridae.) and the node for ‘oomycetes' (NCBI taxonomy: Eukaryota; stramenopiles; oomycetes.) attracted our attention, as this does not correspond to a known virus–host relationship. The association of these two taxonomic nodes, the highest we observed between virus and cells, was statistically significant by both of the two methods we used (*ρ*=0.95, *q*=2.2 × 10^−5^, *ρ*'=0.94, *q*'=0.018; Figure 6), albeit based on a modest number of reads assigned to each of these taxonomic nodes. Thirty-five reads were assigned to the Megaviridae node (31 reads similar to D5 family-predicted DNA helicase/primase sequences (De Silva *et al.*, 2007); 4 reads similar to collagen-like proteins), while 19 reads were assigned to the oomycetes node (homologous to 12 different proteins; Supplementary Table S4). A much larger number of reads were, in fact, assigned to lower taxonomic levels, such as 721 reads assigned to the Mimivirus genus node (that is, ‘Viruses; dsDNA viruses, no RNA stage; Mimiviridae; Mimivirus'). The fact that the majority of the 35 Megaviridae reads corresponded to D5 family primases may be explained by their large gene sizes and usually high sequence conservation (for example, 2880 nt for the Mimivirus L207/L206), a similar observation having been made in a previous marine metagenomic study (Monier *et al.*, 2008b). Consistent with the relatively high ranks of their taxonomic assignments, the reads for the Megaviridae and oomycetes nodes were found to show large divergence from reference protein sequences. The average BLASTx sequence identity for the 35 reads against their closest Megaviridae protein sequences was 50% (ranging from 28% to 88%), and the average sequence identity for the 19 reads assigned to ‘oomycetes' was 58% (30–90%) against their closest known oomycete protein sequences. Their G+C compositions were significantly different with each other (35% for Megaviridae and 48% for oomycete reads, in average; *t*-test, *P*=8.5 × 10^−4^) and comparable with those of their respective reference genomes.

We performed phylogenetic analyses of the 19 reads assigned to the oomycete taxonomic node in an attempt to obtain better taxonomic resolution. Despite their short sizes (∼100 aa) and large evolutionary distances from database homologs, many of these reads appeared related to stramenopiles (12 out of 19 cases), including six cases showing distant yet specific relationships to known oomycete sequences (Supplementary Figures S7-1––S7-12). For the remaining seven reads, their phylogenetic positions were rather poorly resolved and showed no coherent relationship to specific taxonomic groups (Supplementary Figures S7-13––S7-19). A similar analysis of the 31 reads (D5 family proteins) assigned to the Megaviridae node confirmed in most cases their initial taxonomic annotation (Supplementary Figure S8), with some of them assigned close to the root of the viral family. These reads are not closely related to the sequences from CroV (Megaviridae) and phaeoviruses (Phycodnaviridae), the only known NCLDVs parasitizing marine stramenopiles. Phylogenetic analysis was not performed for the four Megaviridae reads similar to collagen-like proteins due to insufficient quality of sequence alignments.

**Figure 7:**
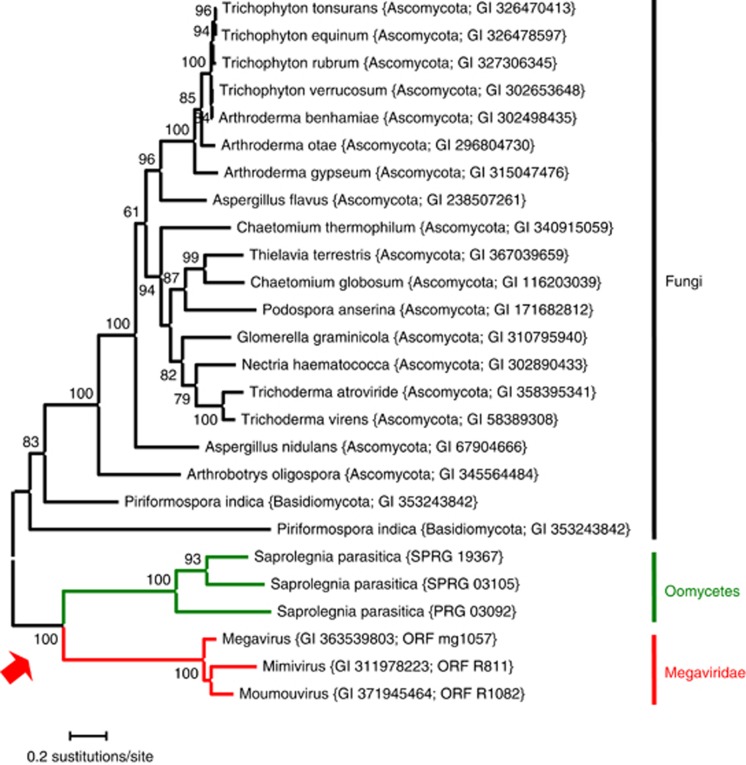
Evidence of horizontal gene transfer between viruses and eukaryotes related to oomycetes. The displayed maximum likelihood tree was generated based on sequences of the Mimivirus hypothetical vWFA domain-containing protein (gi: 311978223) and its homologs using PhyML. The numbers on the branches indicate bootstrap percentages after 100 bootstrap sampling. The tree was mid-point rooted for visualization purpose. The grouping of the Megaviridae and oomycete sequences suggests a gene exchange between the lineage leading to Megaviridae and the lineage leading to oomycetes. Phylogenetic trees for the remaining five putative cases of horizontal gene transfers between these lineages are provided in the Supplementary Figure S9.

If this Megaviridae–stramenopile sympatry revealed by metagenomics reflected an intimate biological interaction (for example, virus–host), we reasoned that an increased rate of genetic exchange might be observable between these organisms. Detection of HGTs between extant genomes of these organisms would thus provide strong independent support for the predicted co-occurrence. We therefore undertook a systematic screening of all publicly available Megaviridae and cellular sequences for hints of potential HGTs. A first reciprocal BLAST best hit search identified 31 candidate HGTs between Megaviridae and cellular organisms (Supplementary Table S5). Surprisingly, the most frequent cellular partner happened to be from the oomycete lineage (six genes). Phylogenetic tree inference provided further evidence that the six genes were likely *bona fide* HGTs (Figure 7 and Supplementary Figure S9). These are a hypothetical protein with a von Willebrand factor type A domain and an in-between ring fingers domain, a putative fatty acid hydroxylase, a hypothetical protein of unknown function, a putative phosphatidylinositol kinase, a putative fucosyltransferase and a putative RNA methylase (S-adenosyl-L-methionine-dependent methyltransferase). For four of these six cases, the monophyletic grouping of the Megaviridae and oomycete sequences was supported by a very high bootstrap value (>97%).

## Discussion

In the late 1970s, Torrella and Morita (1979) revealed unexpected high viral concentrations in aquatic environments using electron microscopy (Bergh *et al.*, 1989). Proctor and Fuhrman (1990) then discovered that viruses were quantitatively important components of marine food webs through the observation of numerous bacteria visibly infected by viruses. Ever since these pioneering works, a large body of research continuously revealed the fascinating ecological and evolutionary functions of viruses, including NCLDVs in marine environments (Wilson *et al.*, 2005; Sullivan *et al.*, 2006; Frada *et al.*, 2008; Nagasaki, 2008; Moreau *et al.*, 2010; Danovaro *et al.*, 2011; Breitbart, 2012).

The abundance of NCLDV genomes was found to be in the range from 4 × 10^3^ to 1.7 × 10^5^ genomes ml^−1^ for the TOP photic layer samples. Our indirect metagenomic estimate of virus abundance is likely to be affected in two opposite ways: overestimation, for instance, due to actively replicating viral genomic DNA in infected small eukaryotic cells, and underestimation due to smaller or larger virion particles not being captured by our size fractionation or reduced efficiency of DNA extraction for encapsidated genomes. In fact, a substantial proportion of prasinovirus OtV particles (∼120 nm in diameter) cannot be retained on the 0.2-μm membrane (Grimsley and Clerissi, data not shown). Furthermore, underestimation was likely to be compounded by the fact that most NCLDV-infected cells are >1.6 μm and thus were excluded from our size fraction. Filtration efficiency is another pitfall of quantitative estimates. Size of retained microbes may vary during pre- and retention filtration (progressively excluding smaller infected cells and retaining smaller NCLDVs than the filter's nominal pore sizes), though we rarely encountered filter clogging for the samples analyzed in this study. Regarding our experimental measurements, we used well-established methods for prokaryotic cell counts (FC and epifluorescence microscopy), which distinguish cells from many viruses, including marine NCLDVs (Jacquet *et al.*, 2002). Yet, we cannot exclude the possibility of the existence of cell-sized (and -shaped) marine viruses that could not be discriminated from cells by these methods. Our metagenomic based ratio of NCLDVs to prokaryotes (<5%) then suggests that the resulting prokaryote overestimation (due to contaminated large viruses) could be 5% at most. Therefore, our estimate should be considered a first approximation for genome abundance of core gene containing NCLDVs in the analyzed size fraction. An early metagenomic survey showed that only 0.02% of the total predicted proteins from the GOS metagenomes corresponded to Mimivirus homologs (Williamson *et al.*, 2008). Such a small proportion cannot be directly compared with the higher genome abundance estimate we obtained in this study (that is, 10% of bacterial genomes in the GOS data), as gene abundance estimates are heavily dependent on genome diversity and the availability of reference genomes. We consider that our marker gene-based approach is rather suitable to quantify the abundance of NCLDV genomes, given the limited number of sequenced NCLDV genomes and the large genomic diversity observed even within a single family of NCLDVs. The abundance of eukaryotic organisms (mainly unicellular) in marine microbial assemblages is typically three orders of magnitude lower than that of prokaryotes (Suttle, 2007; Massana, 2011). In the euphotic zone of the Sargasso Sea, phototrophic/heterotrophic nanoplankton (2–20 μm) and phototrophic/heterotrophic microplankton (20–200 μm) were found to amount to only 0.3% of bacterial abundance (Caron *et al.*, 1995). Therefore, the predicted NCLDV genome abundance by the present study suggests that NCLDVs equal or even outnumber eukaryotic organisms in the photic layer of the sea. In other words, our suggested NCLDV/eukaryote ratio is not unlike the ratio of phage/bacteria in seawater (Suttle, 2007). Whole-genome amplification and sequencing of single microbial cells/viruses is becoming a powerful tool in revealing genomic contents of environmental uncultivated microorganisms (Allen *et al.*, 2011; Yoon *et al.*, 2011). These studies reveal that a substantial fraction of the unicellular organisms in a population may be infected by viruses. The estimated relative genome abundance of NCLDVs (3% and 10% of bacteria in the TOP and GOS data sets, respectively) suggests that such single virus genomics approaches will be helpful in analyzing uncultivated marine NCLDVs from size-fractioned natural water samples.

The predicted abundance of NCLDV genomes was found to vary from 10^4^ to 10^5^ genomes ml^−1^ for most of the TOP euphotic samples. Interestingly, the suggested variation in the abundance of NCLDVs (at a high taxonomic level) across sampling sites makes a very sharp contrast with the known and more remarkable fluctuations (spanning more than several orders of magnitudes) in the abundance of specific viral species/strains measured in time series monitoring (Tomaru *et al.*, 2004). Moreover, our phylogenetic (Figure 4) and fragment recruitment analyses (Supplementary Figure S5) indicated that numerous distinct genotypes exist (for the Megaviridae family and the prasinovirus clade) in the analyzed samples (even within a single sample). It has been recently suggested (Rodriguez-Brito *et al.*, 2010) that dominant phage and bacterial taxa in microbial communities persist over time in stable ecosystems but their populations fluctuate at the genotype/strain levels in a manner predictable by the ‘killing-the-winner' hypothesis (Winter *et al.*, 2010). Multiple and perpetual prey–predator interactions and functional redundancy across species/genotypes may lead to the apparent stability they observed in the community composition at high taxonomic levels. A similar mechanism might be acting on marine NCLDV-host communities. The relatively stable NCLDV sequence abundance across geographically distant locations may be caused by compensating local community changes at low taxonomic levels, in which diverse NCLDV strains are involved in the control of specific eukaryotic host populations.

Isolation of new viruses requires host cultures. Among known hosts of NCLDVs, amoebas of the *Acanthamoeba* genus have been the most efficient laboratory hosts to isolate new NCLDVs from aquatic samples (Arslan *et al.*, 2011, Boyer *et al.*, 2009, La Scola *et al.*, 2010, Thomas *et al.*, 2011). Taxon association analysis on the TOP data set hinted at an unexpected sympatric association between Megaviridae and stramenopiles possibly distantly related to oomycetes. The two sets of reads involved in this correlation showed a clear difference in their G+C compositions. This rather suggests two distinct source organisms for these reads. Yet, an alternative scenario is that they originated from a single organism (a virus very recently acquiring cellular genes or a cellular organism with recently integrated viral genomes). In this case, the taxonomic association would not correspond to a direct observation of the co-occurring organisms but would be a by-product of very recent genetic exchanges between Megaviridae and oomycete relatives. However, there is no known example of a lysogenic virus of the Megaviridae family and recent research shows little evidence for recent HGTs between marine NCLDVs and eukaryotes (Monier *et al.*, 2007; Derelle *et al.*, 2008; Moreira and Brochier-Armanet, 2008; Filee and Chandler, 2010).

Oomycetes are filamentous eukaryotic microorganisms resembling fungi in many aspects of their biology, but they form a totally distinct phylogenetic group within the stramenopile (heterokont) supergroup (Richards *et al.*, 2011). Some of them are devastating crop pathogens, such as *Phytophthora infestans* causing late blight of potato (Haas *et al.*, 2009), but others include pathogens of fishes and algae, such as the water mold *Saprolegnia parasitica* causing diseases in fishes (Kale and Tyler, 2011) and *Eurychasma dicksonii* infecting marine brown algae (Grenville-Briggs *et al.*, 2011). To our knowledge, there is no report of a giant virus infecting oomycetes. However, other stramenopile lineages include *C. roenbergensis* (stramenopiles; Bicosoecida; Cafeteriaceae; Cafeteria) and brown algae (stramenopiles; Phaeophyceae; Ectocarpales), which are hosts of known NCLDVs (CroV and phaeoviruses). Yet, our sequence analysis of the predicted Megaviridae reads indicated that they are not closely related to the sequences from these viruses. The possible promiscuity of these two marine dwellers was further supported by the identification of several putative HGTs between Megaviridae and oomycete genomes. Incidentally, some of the analyzed trees exhibited oomycete homologs near the Phycodnaviridae clade (Supplementary Figure S8) and several fungal homologs adjacent to the Megaviridae/oomycete clade (Figure 7 and Supplementary Figure S9-1). Multiple gene transfers have been described from fungi to oomycetes, and the suggestion was made that they contributed to the evolution of the pathogenicity of oomycetes (Richards *et al.*, 2011).

We found in the literature an intriguing coincidence in the biogeography of Megaviridae and oomycetes. Megaviridae was identified as a dominant family of NCLDVs in a sample from a mangrove forest (Monier *et al.*, 2008a), while 20 years earlier marine oomycetes (for example, *Phytophthora vesicula*) were described as the major decomposers of mangrove leaves (Newell *et al.*, 1987). Taken together, these observations lead us to hypothesize that there is a yet unrecognized close interaction between Megaviridae and stramenopiles (distantly related to oomycetes), either as a direct virus/host couple (Monier *et al.*, 2009) or through co-infection of a common third partner (Ogata *et al.*, 2006; Boyer *et al.*, 2009). Limitations in the available genome data for marine stramenopiles and the scope of the present TOP data set, which targeted the girus/prokaryote size fraction, make it difficult to obtain finer taxonomic resolutions for the potential eukaryotic counterpart.

The present work provides a proof of principle that metagenomic sequence analyses promise to shed new light on the biodiversity of marine viruses and their interactions with potential hosts. Larger sets of environmental sequence data from diverse locations and different size fractions, such as those from remaining Tara Oceans samples, will be useful not only to test our ‘Megaviridae–stramenopile' hypothesis but also to provide a larger picture of NCLDV–eukaryote interactions.

## Figures and Tables

**Table 1:** General description of the samples analyzed in this study

*Name*	*Station number*	*Region*	*Marine system type*	*Depth (m)*	*Location*^a^	*T (°C)*	*Salinity (psu)*	*Chl a (mg Chl a m*^*−3*^)	*Date and time (UTC)*^a^	*Sample identifiers*
3_S	3	Atlantic Ocean	Open ocean	SRF	36°43.520'N 10°28.250'W	NA	NA	NA	2009/09/13 10:40	TARA-Y200000001 (A6.1)
4_S	4	Atlantic Ocean	Open ocean	SRF	36°33.200'N 6°34.010'W	NA	NA	NA	2009/09/15 10:15	TARA-Y200000002 (A11)
6_S	6	Mediterranean Sea	Enclosed sea	SRF	36°31.239'N 4°0.443'W	17.0	37.35	3.121	2009/09/21 14:49	TARA-Y200000003 (A32)
7_S	7	Mediterranean Sea	Enclosed sea	SRF	37°2.321'N 1°56.99'W	23.8	37.48	0.075	2009/09/23 17:05	TARA-A200000113
7_D	7	Mediterranean Sea	Enclosed sea	DCM (42 m)	37°2.321'N 1°56.99'W	17.8	37.09	0.296	2009/09/23 17:05	TARA-A200000159
23_S	23	Mediterranean Sea	Enclosed sea	SRF	42°10.462'N 17°43.163'E	17.1	38.22	0.036	2009/11/18 12:44	TARA-E500000066
23_D	23	Mediterranean Sea	Enclosed sea	DCM (56 m)	42°10.462'N 17°43.163'E	16.0	38.30	0.119	2009/11/18 12:44	TARA-E500000081
30_S	30	Mediterranean Sea	Enclosed sea	SRF	33°55.077'N 32°53.622'E	20.4	39.42	0.025	2009/12/14 12:44	TARA-A100001568
31_S	31	Red Sea	Enclosed sea	SRF	27°8.100'N 34°48.400'E	25.0	39.91	0.005	2010/01/09 10:03	TARA-A100001568
36_S	36	Arabian Sea	Semi-enclosed sea	SRF	20°49.053'N 63°30.727'E	26.0	36.53	0.047	2010/03/12 10:36	TARA-Y100000022
38_S	38	Arabian Sea	Semi-enclosed sea	SRF	19°2.318'N 64°29.620'E	26.3	36.62	0.052	2010/03/15 03:45	TARA-Y100000288
38_Z	38	Arabian Sea	Semi-enclosed sea	OMZ (350 m)	19°2.103'N 64°33.825'E	14.7	36.00	0.002	2010/03/16 06:14	TARA-Y100000294
39_S	39	Arabian Sea	Semi-enclosed sea	SRF	18°34.213'N 66°29.167'E	27.4	36.29	0.026	2010/03/18 09:56	TARA-Y100000029
39_Z	39	Arabian Sea	Semi-enclosed sea	OMZ (270 m)	18°44.043'N 66°23.375'E	15.6	35.91	0.003	2010/03/20 08:17	TARA-Y100000031
43_S	43	Indian Ocean	Lagoon	SRF	4°39.582'N 73°29.128'E	30.0	34.49	0.075	2010/04/05 08:50	TARA-Y100000074
46_S	46	Indian Ocean	Lagoon	SRF	0°39.748'S 73°9.664'E	30.1	35.11	0.050	2010/04/15 02:40	TARA-Y100000100
49_S	49	Indian Ocean	Open ocean	SRF	16°48.497'S 59°30.257'E	28.3	34.49	0.024	2010/04/23 10:29	TARA-Y100000120

Abbreviations: DCM, deep chlorophyll maximum; NA, not applicable; OMZ, oxyzen minimum zone; SRF, surface; UTC, Coordinated Universal Time.

a Locations, date and time correspond to events for the collection of contextual physicochemical data. Events for water sampling could slightly differ from these values.

**Table 2:** Quality-controlled Tara Oceans pyrosequence data

*Sample name*	*Total size (bp)*	*Number of reads*	*G+C (%)*	*Average size (bp)*	*Number of predicted ORFs*	*Average ORF size (aa)*
3_S	21 533 646	63 994	37	336	65 656	99
4_S	52 953 075	140 754	38	376	149 018	108
6_S	36 129 806	95 255	48	379	98 996	111
7_S	98 750 180	332 049	38	297	335 408	90
7_D	279 389 388	1 117 888	37	250	1 013 853	81
23_S	67 695 268	196 190	39	345	201 447	101
23_D	83 539 478	239 447	38	349	246 948	102
30_S	89 180 466	256 028	37	348	268 616	101
31_S	245 463 121	614 743	39	399	660 949	114
36_S	245 945 064	737 506	39	333	757 448	100
38_S	214 253 370	601 110	39	356	631 351	103
38_Z	223 188 575	638 843	45	349	659 041	104
39_S	233 273 851	590 664	43	395	629 501	114
39_Z	249 558 778	679 589	46	367	708 056	108
43_S	167 515 516	529 506	37	316	545 641	93
46_S	251 310 870	648 425	41	388	689 641	112
49_S	222 417 021	680 573	43	327	696 974	98

Abbreviation: ORF, open reading frame.

**Table 3:** Examples of positive and negative viral-cell associations

*Taxon 1*	*Taxon 2*	*ρ*	*q*	*ρ*'	*q*'
*Co-occurrence*					
Viruses; dsDNA viruses, no RNA stage; Mimiviridae	Eukaryota; stramenopiles; Oomycetes	0.949	2.22E-05	0.939	1.7E-02
Viruses; dsDNA viruses, no RNA stage; Iridoviridae; Lymphocystivirus; unclassified Lymphocystivirus	Bacteria; Tenericutes; Mollicutes; Mycoplasmataceae	0.883	1.44E-03	—	—
Viruses; unclassified phages; environmental samples	Bacteria; Cyanobacteria; environmental samples	0.864	2.92E-03	—	—
Viruses; dsDNA viruses, no RNA stage; Caudovirales; Siphoviridae	Eukaryota; Alveolata; Apicomplexa; Aconoidasida; Piroplasmida	0.861	3.26E-03	—	—
Viruses; dsDNA viruses, no RNA stage; Herpesvirales; Herpesviridae; Gammaherpesvirinae	Bacteria; Proteobacteria; Gammaproteobacteria; Thiotrichales; Thiotrichaceae	0.853	4.20E-03	—	—
Viruses; dsDNA viruses, no RNA stage; Phycodnaviridae	Bacteria; Proteobacteria; Gammaproteobacteria; Alteromonadales; Alteromonadales genera incertae sedis	0.838	6.30E-03	—	—
Viruses; dsRNA viruses; Reoviridae; Sedoreovirinae; Mimoreovirus	Eukaryota; Metazoa; Chordata; Craniata	0.834	6.98E-03	—	—
Viruses; dsDNA viruses, no RNA stage; Herpesvirales; Herpesviridae; Gammaherpesvirinae	Bacteria; Chloroflexi; Thermomicrobiales; Thermomicrobiaceae; Thermomicrobium	0.830	7.61E-03	—	—
Viruses; dsDNA viruses, no RNA stage; Herpesvirales; Herpesviridae; Gammaherpesvirinae	Bacteria; Proteobacteria; Magnetococcus	0.825	8.53E-03	—	—
Viruses; dsDNA viruses, no RNA stage; Phycodnaviridae; unclassified Phycodnaviridae	Eukaryota; Viridiplantae; Chlorophyta; Prasinophyceae; Mamiellales	0.821	9.36E-03	—	—
Viruses; dsDNA viruses, no RNA stage; Herpesvirales; Herpesviridae; Gammaherpesvirinae	Bacteria; Acidobacteria; Solibacteres; Solibacterales; Solibacteraceae	0.820	9.51E-03	—	—
Viruses; dsDNA viruses, no RNA stage; Herpesvirales; Herpesviridae; Gammaherpesvirinae	Bacteria; Proteobacteria; Deltaproteobacteria; Desulfobacterales; Desulfobacteraceae	0.820	9.51E-03	—	—
Viruses; dsDNA viruses, no RNA stage; Caudovirales; Myoviridae; T4-like viruses	Bacteria; Cyanobacteria; environmental samples	0.819	9.71E-03	—	—
Viruses; dsDNA viruses, no RNA stage; Caudovirales; Podoviridae; Autographivirinae	Bacteria; Cyanobacteria; environmental samples	0.817	1.02E-02	—	—
Viruses; dsDNA viruses, no RNA stage	Eukaryota; Alveolata; Ciliophora; Intramacronucleata; Spirotrichea	0.803	1.36E-02	—	—
Viruses; dsDNA viruses, no RNA stage; Caudovirales; Podoviridae; N4-like viruses	Bacteria; Firmicutes; Clostridia; Clostridiales; Peptococcaceae	0.802	1.38E-02	—	—
Viruses; dsDNA viruses, no RNA stage; Caudovirales	Eukaryota; Alveolata; Apicomplexa; Aconoidasida; Piroplasmida	0.802	1.39E-02	—	—
Viruses; dsDNA viruses, no RNA stage; Viruses; dsDNA viruses, no RNA stage; unclassified dsDNA viruses	Bacteria; Proteobacteria; Alphaproteobacteria; Rickettsiales; SAR11 cluster	0.801	1.39E-02	—	—
Viruses; dsDNA viruses, no RNA stage; Phycodnaviridae; Phaeovirus	Eukaryota; stramenopiles; Actinophryidae; Actinophrys	0.801	1.39E-02	—	—
Viruses; dsDNA viruses, no RNA stage; Phycodnaviridae; unclassified Phycodnaviridae	Eukaryota; Viridiplantae; Chlorophyta; Prasinophyceae; environmental samples	0.800	1.42E-02	—	—
*Mutual exclusion*					
Viruses; dsDNA viruses, no RNA stage; Caudovirales; Myoviridae; phiKZ-like viruses	Eukaryota; Euglenozoa; Kinetoplastida; Trypanosomatidae; Leishmania	−0.742	3.32E-02	—0.804	1.72E-02
Viruses; dsDNA viruses, no RNA stage; Iridoviridae; Ranavirus	Bacteria; candidate division OP8; environmental samples	−0.751	2.95E-02	−0.695	3.83E-02
Viruses; dsDNA viruses, no RNA stage; Caudovirales; Myoviridae; phiKZ-like viruses	Eukaryota; Rhodophyta; Bangiophyceae; Cyanidiales; Cyanidiaceae	—	—	−0.659	2.95E-02
Viruses; dsDNA viruses, no RNA stage; Caudovirales; Myoviridae; phiKZ-like viruses	Bacteria; Spirochaetes; Spirochaetales; Spirochaetaceae	—	—	−0.715	3.95E-02

Abbreviation: dsDNA, double-stranded DNA.

Statistical significance of taxon associations was assessed by two methods. *ρ* (Spearman's correlation coefficient) and *q* (false discovery rate) were calculated by the first method and *ρ*' (Spearman's correlation coefficient) and *q*' (false discovery rate) were calculated by a more stringent second method. See Materials and methods for details.
