# Supplementary material for: Exploring nucleo-cytoplasmic large DNA viruses in Tara Oceans microbial metagenomes
Source: ISME J. 2013 Apr 11;7(9):1678–95. doi: 10.1038/ismej.2013.59 (PMC3749498; doi:10.1038/ismej.2013.59)
Supplement: Supplementary Figures [file ismej201359x1.pdf]

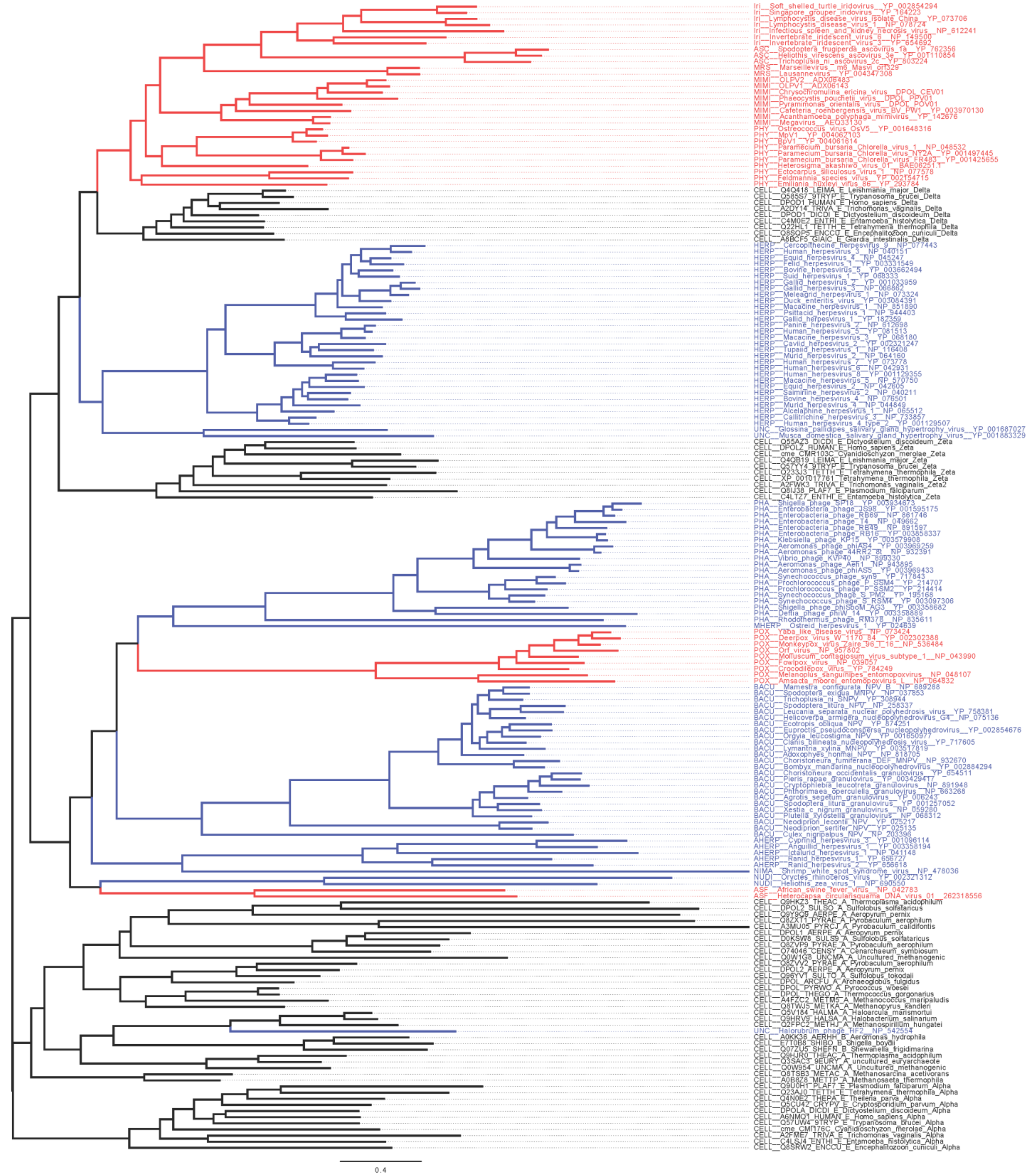

Figure S1. PolB reference tree used for phylogenetic mapping. This maximum likelihood (ML) tree was built based on type B DNA polymerase homologs (PolB) from diverse groups of viruses and cellular organisms and was used to classify metagenomic PolB-like reads (Figure 4) across different groups of NCLDVs. Color code is as follows: red (NCLDVs), blue (other viruses), black (eukaryotes, Archaea and Bacteria).

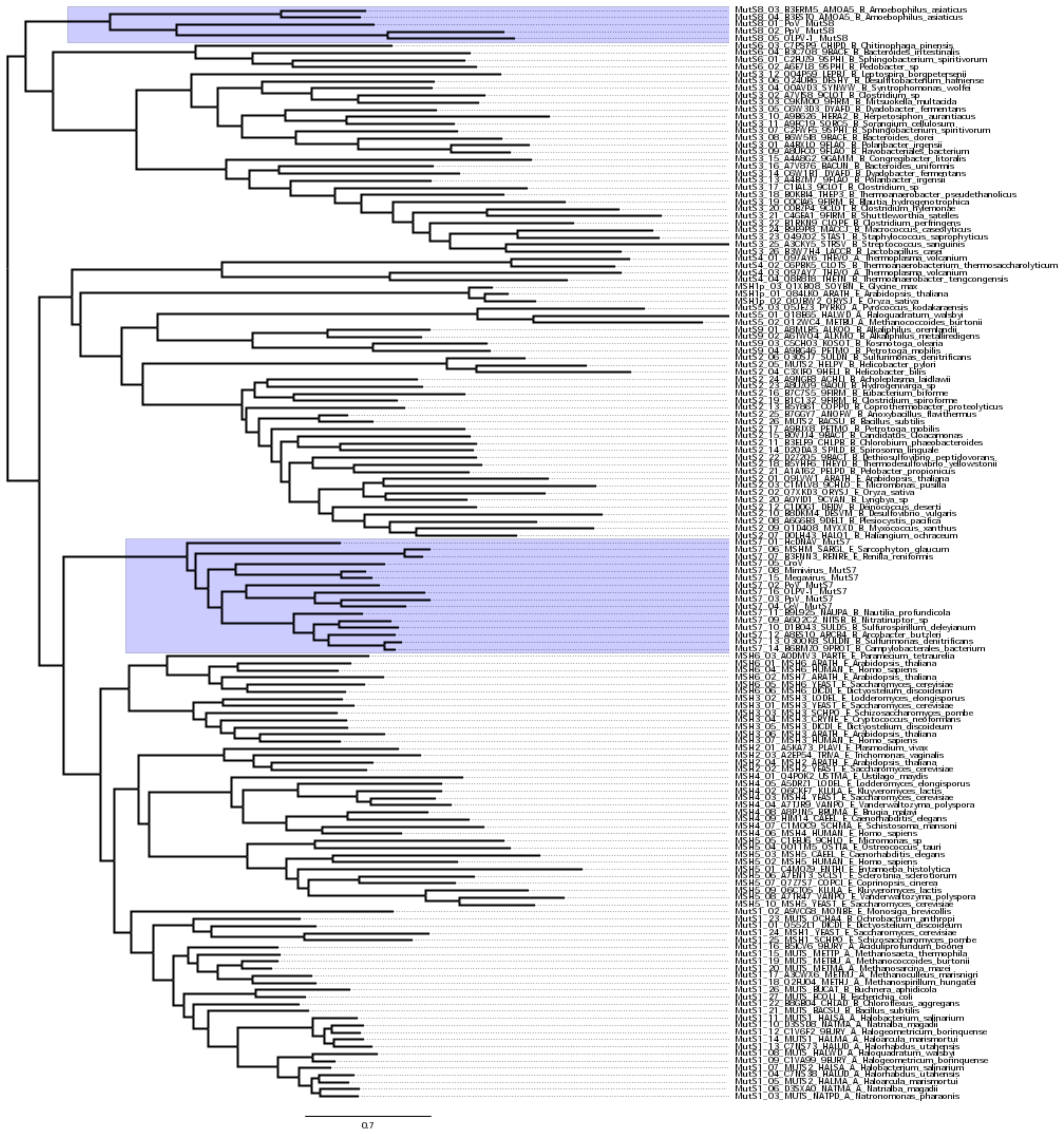

Figure S2. MutS reference tree used for phylogenetic mapping. This ML-tree was built based on protein sequences from different subfamilies of MutS homologs, including sequences from NCLDVs, eukaryotes, Bacteria and Archaea. The tree served as a reference to phylogenetically place metagenomic MutS-like reads for their classification (Figure S6a). Highlighted in blue are branches for the MutS7 and MutS8 subfamilies, which include known representatives from NCLDVs.

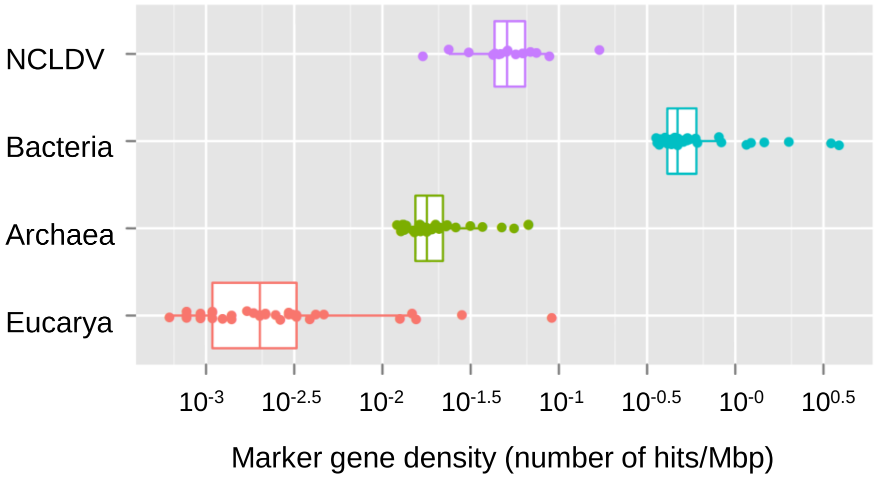

Figure S3. Metagenome-based relative abundance of NCLDV and cellular genomes in the GOS data set. Forty GOS metagenomes (0.1-0.8 µm size fraction) were pooled and analyzed as a single data set to generate this plot. Each dot in the plot represents the density of one of the marker genes used in this study (16 markers for NCLDVs and 35 markers for cellular genomes). The estimated abundance of NCLDVs genomes corresponds to 10% of bacterial genomes.

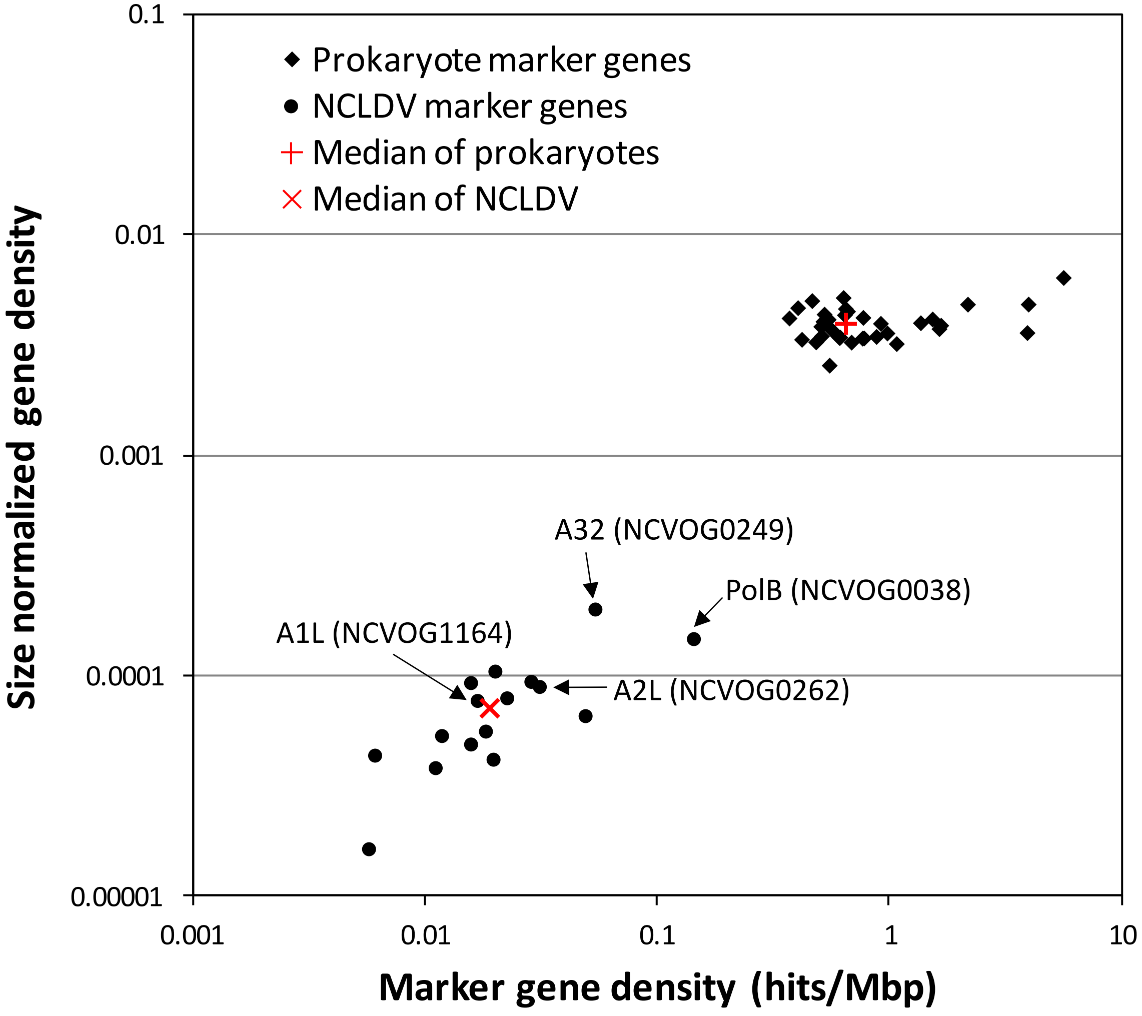

Figure S4. Effect of size normalization on the estimate of the relative genome abundance between NCLDVs and prokaryotes. Size normalized marker gene densities for NCLDVs (16 markers) and prokaryotes (35 markers) are plotted against marker gene densities (hits/Mbp) without size-normalization. Normalization was performed by dividing the marker gene density by the length of the multiple alignment of each marker gene model. NCLDV/Prokaryote median value ratio was 0.029 without normalization, while it was reduced to 0.018 (38% reduction) when the normalization process was introduced. Four marker genes that are conserved in most NCLDV genomes are indicated by black arrows. Other 12 NCLDV marker genes are conserved in most of the currently sequenced viruses from the Megaviridae and Phycodnaviridae families.

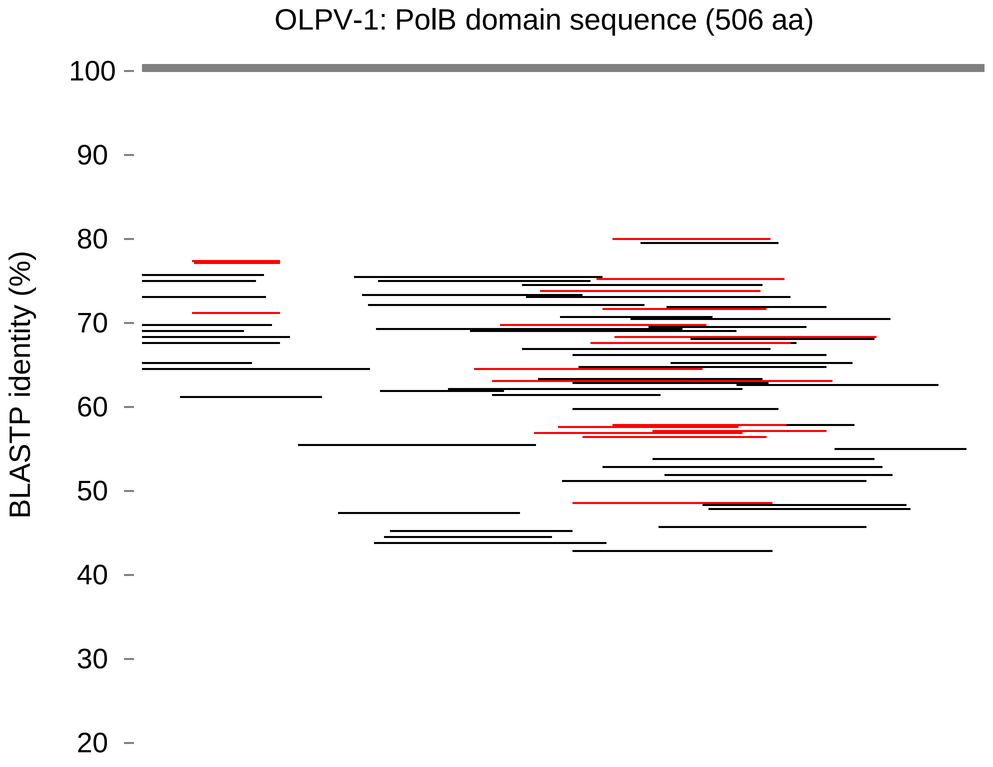

Figure S5. Fragment recruitment plot along the OLPV-1 PolB domain sequence. Reads from a selected single sample (Station 38/surface) are highlighted by red. This plot suggests a high level of richness among the detected metagenomic reads related to the OLPV-1 PolB sequence (even within a single sample).

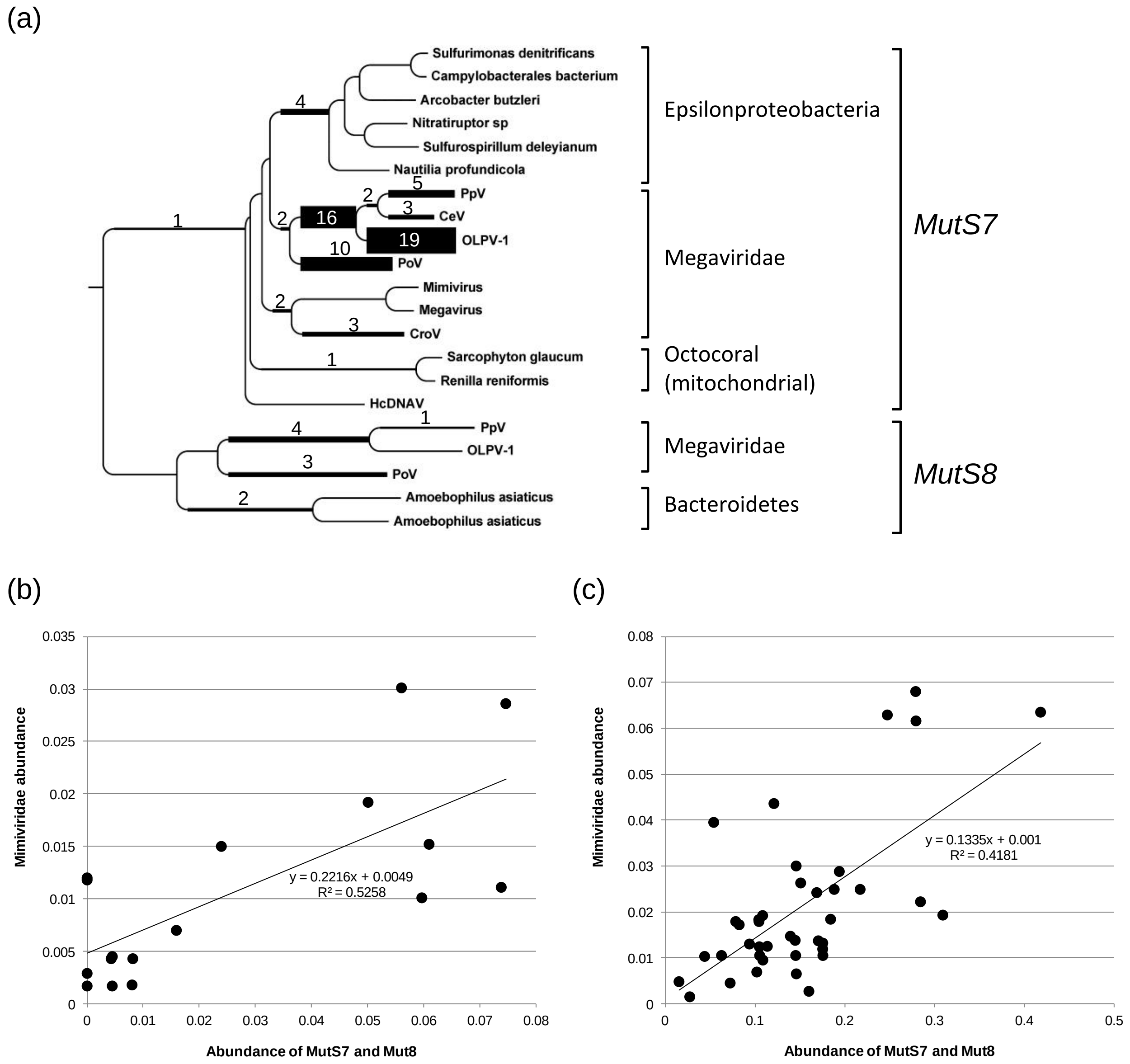

Figure S6. Correlation between the abundance of Megaviridae marker genes and MutS7/8 subfamilies. (a) Tara metagenomic reads assigned to the MutS7 and MutS8 subfamilies according to a Pplacer phylogenetic mapping analysis based on a reference tree derived from a large data set composed of diverse viral and cellular sequences (Supplementary Figure S2). Only part of the mapping result relevant to MutS7 and MutS8 subfamilies is shown here. (b) Correlation between the abundance of Magaviridae genomes assessed by the average marker gene density and the abundance of MutS7/8 sequences in the Tara metagenomes. (c) Correlation between the abundance of Magaviridae genomes assessed by the average marker gene density and the abundance of MutS7/8 sequences in the GOS metagenomes. The observed correlations indicate that putative Megaviridae captured in the metagenomic data encode these Girus-specific MutS subfamilies.

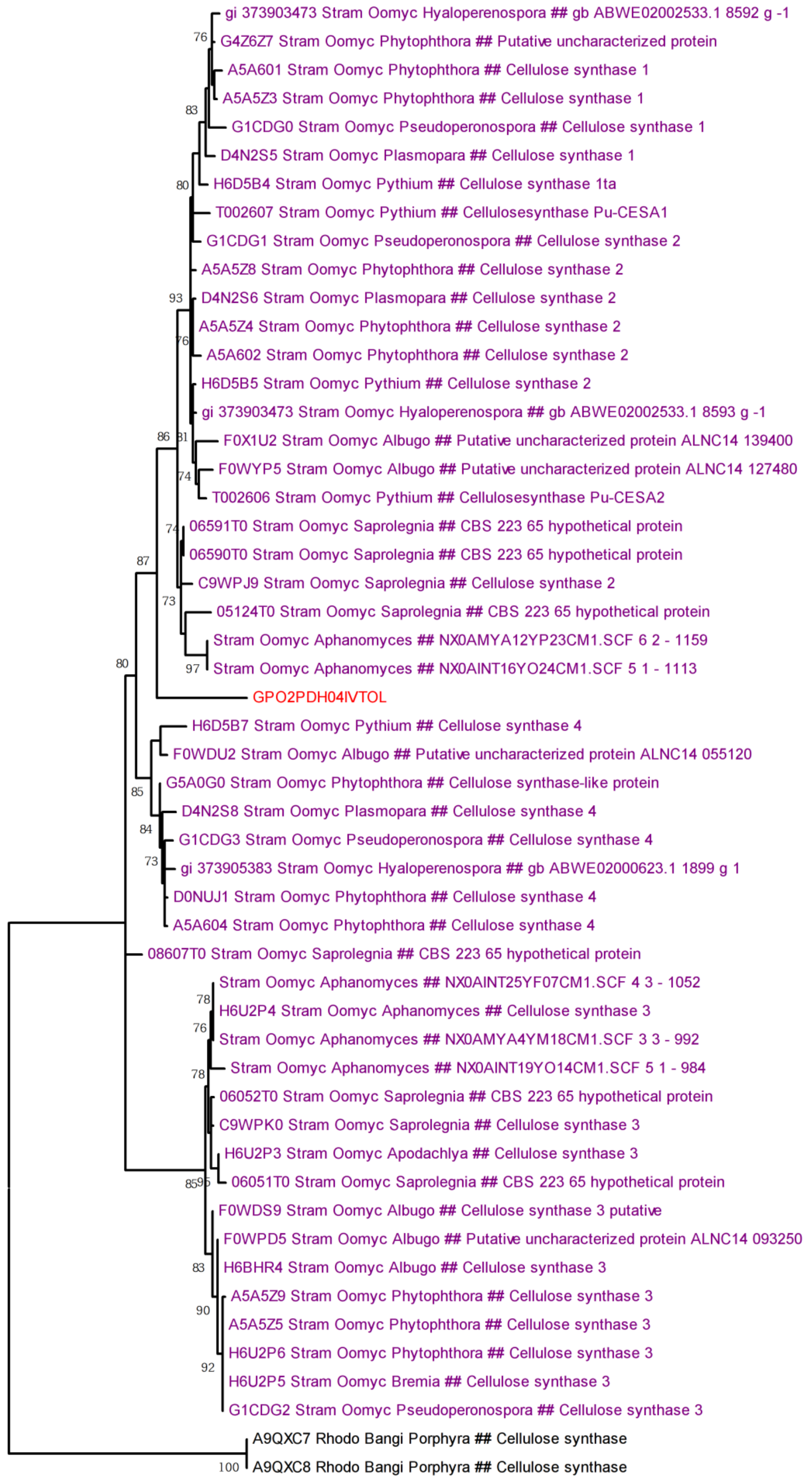

Figure S7. Maximum likelihood phylogenetic analysis of the nineteen reads assigned to the oomycetes taxonomic node by similarity searches. (S7-1) Cellulose synthase like sequences (GPO2PDH04IVTOL).

Translated sequences for the reads (identified by BLASTX HSPs) were aligned to their respective homologous sequences retrieved from the "UniRef100+stramenopiles" database by MUSCLE. After GBLOCKS and manual curation steps, phylogenetic analyses were performed using PhyML with the approximate likelihood ratio test for branch support (SH-like method). In addition to PhyML, we used Pplacer in case the use of the whole regions of the database sequences improved the resolution of the tree (i.e. Figure S7-10).

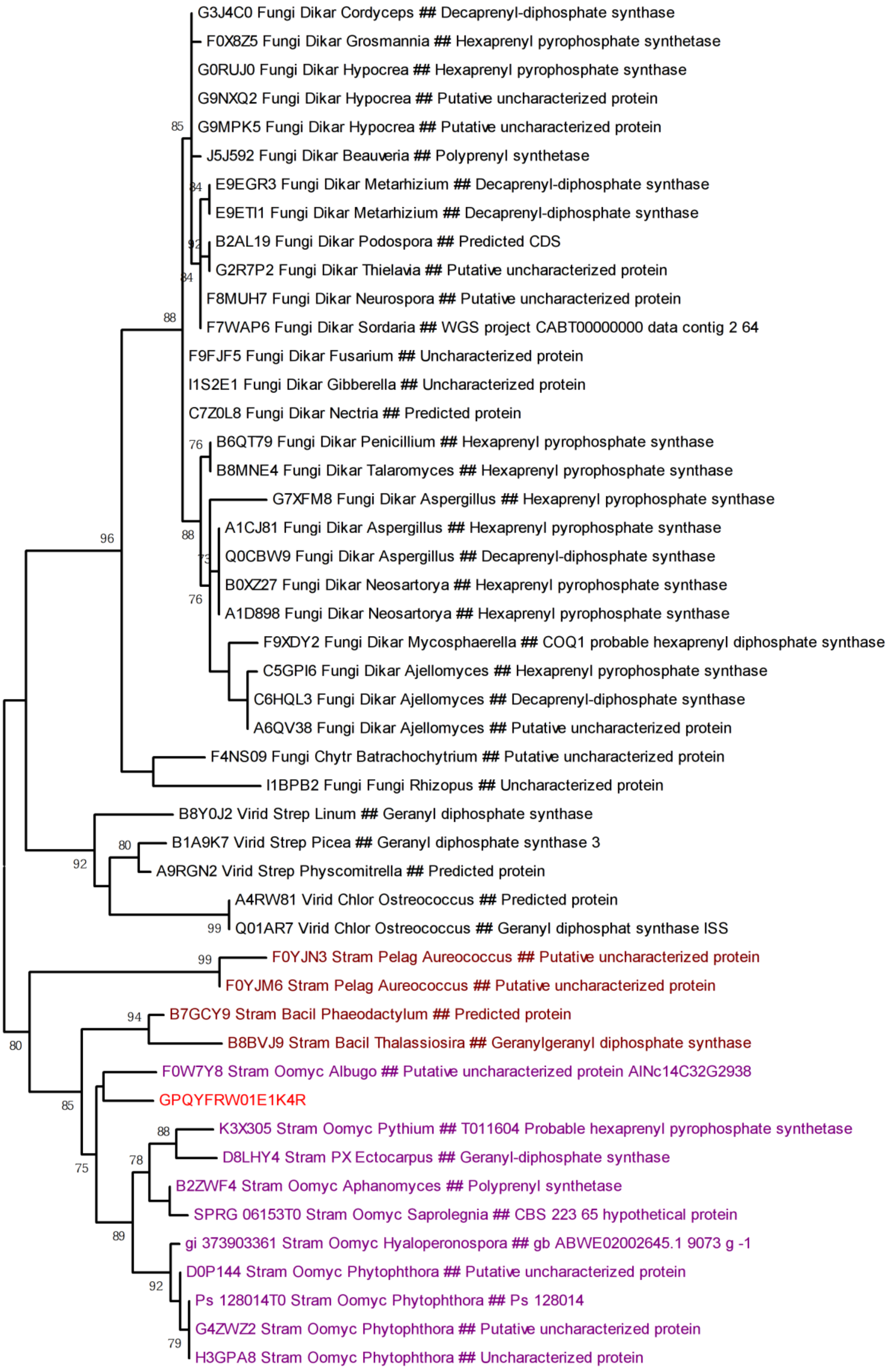

Figure S7-2. Geranylgeranyl diphosphage synthase homologs (GPQYFRW01E1K4R).

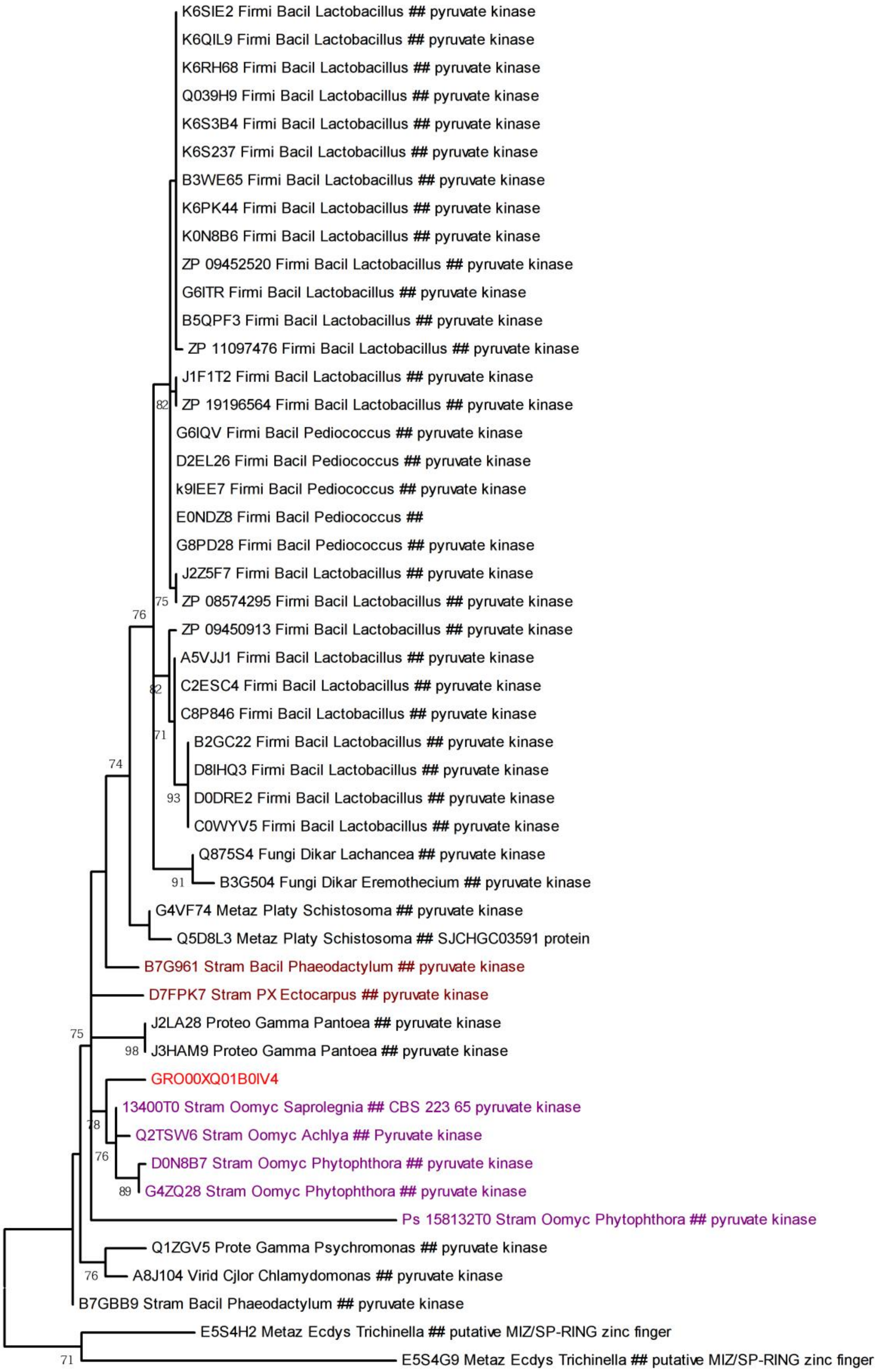

Figure S7-3. Pyruvate kinase (GRO00XQ01B0IV4).

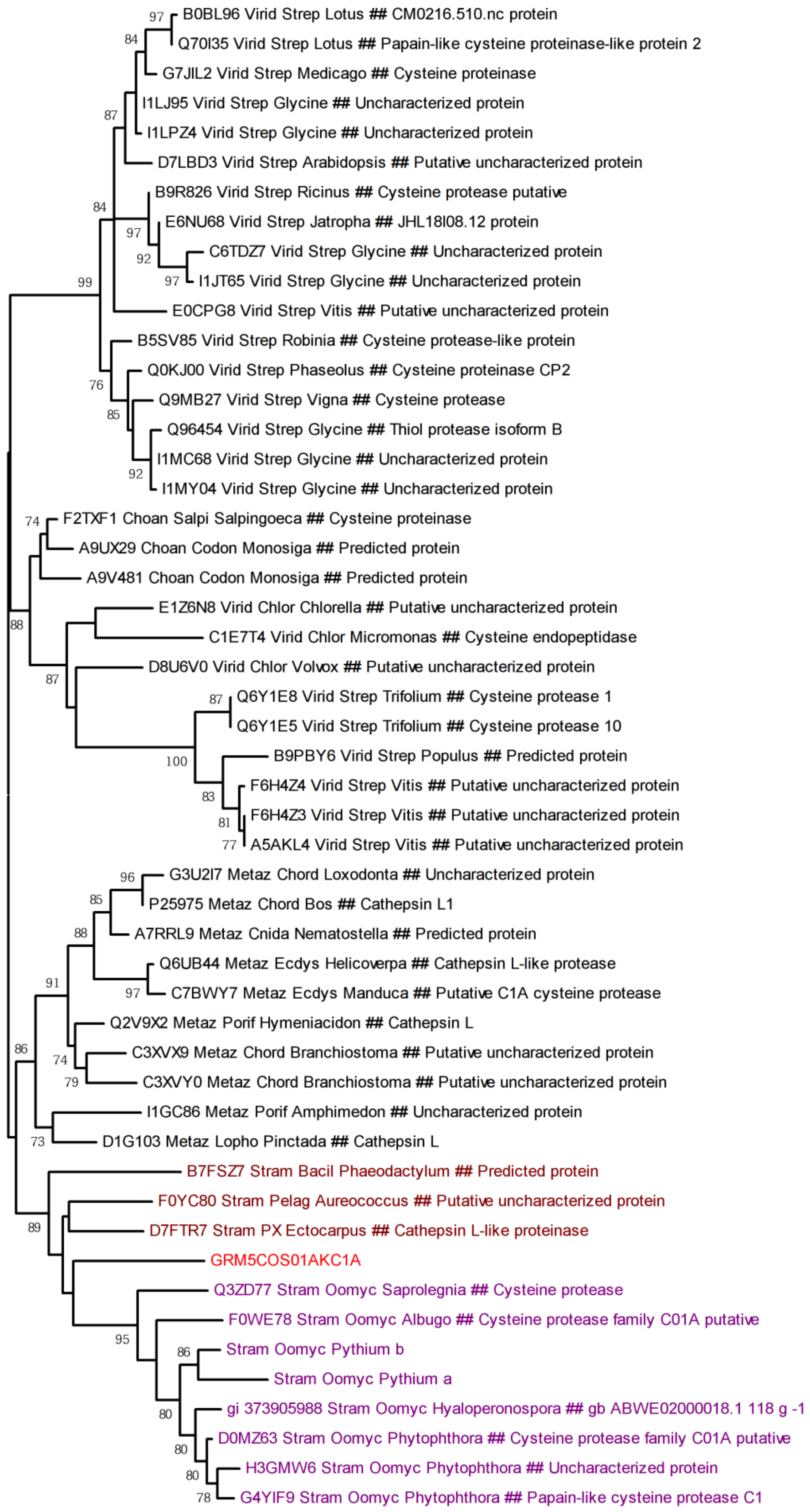

Figure S7-4. Cysteine protease homologs (GRM5COS01AKC1A).

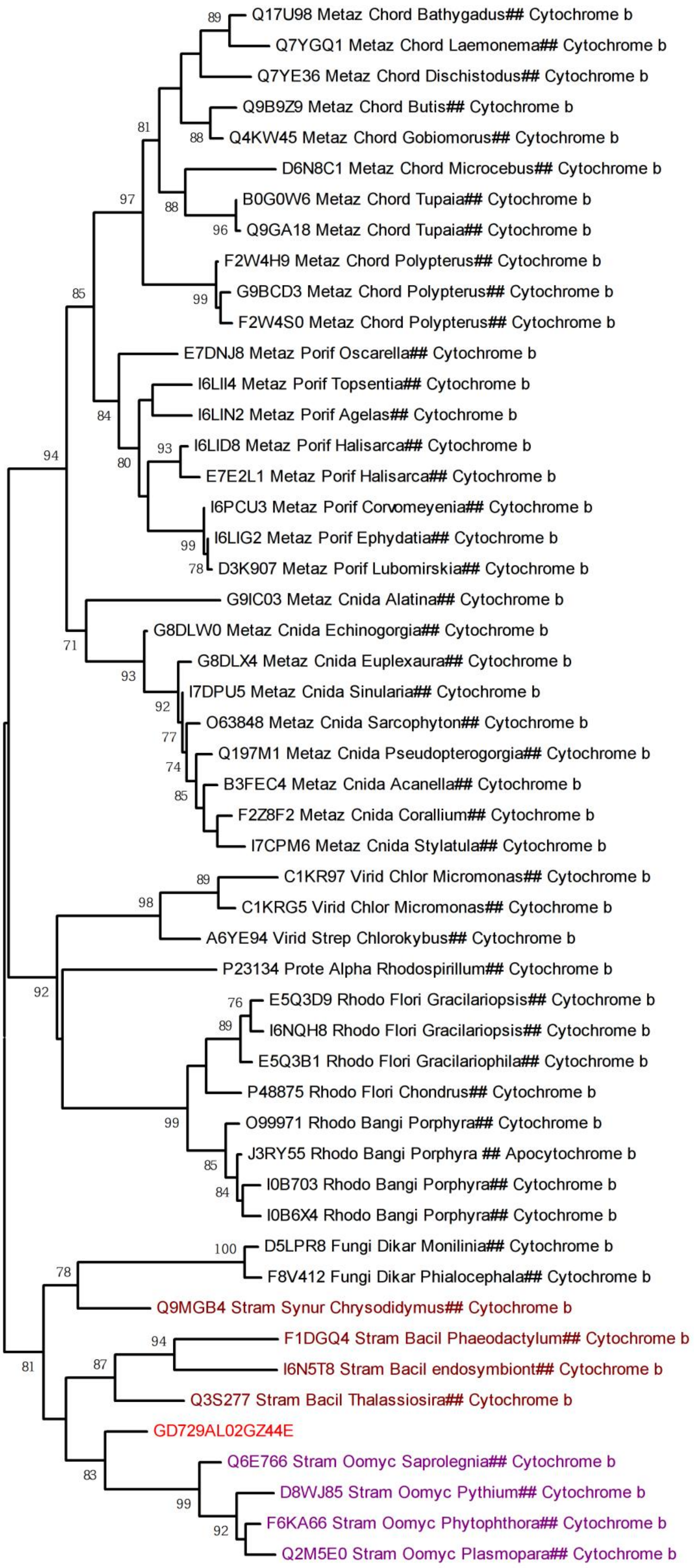

Figure S7-5. Cytochrome b homologs (GD729AL02GZ44E)

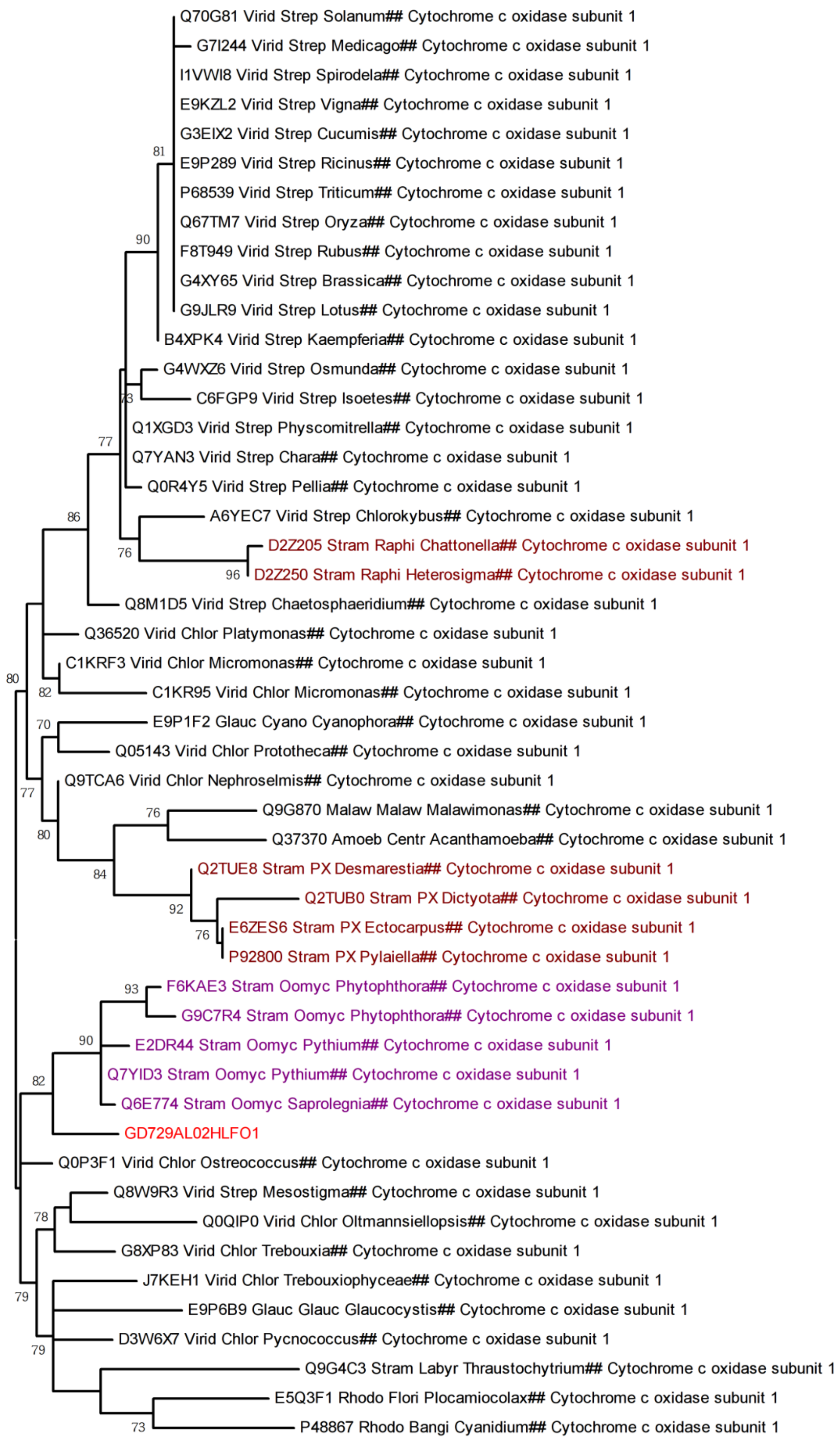

Figure S7-6. Cytochrome c oxidase subunit 1 (GD729AL02HLFO1).

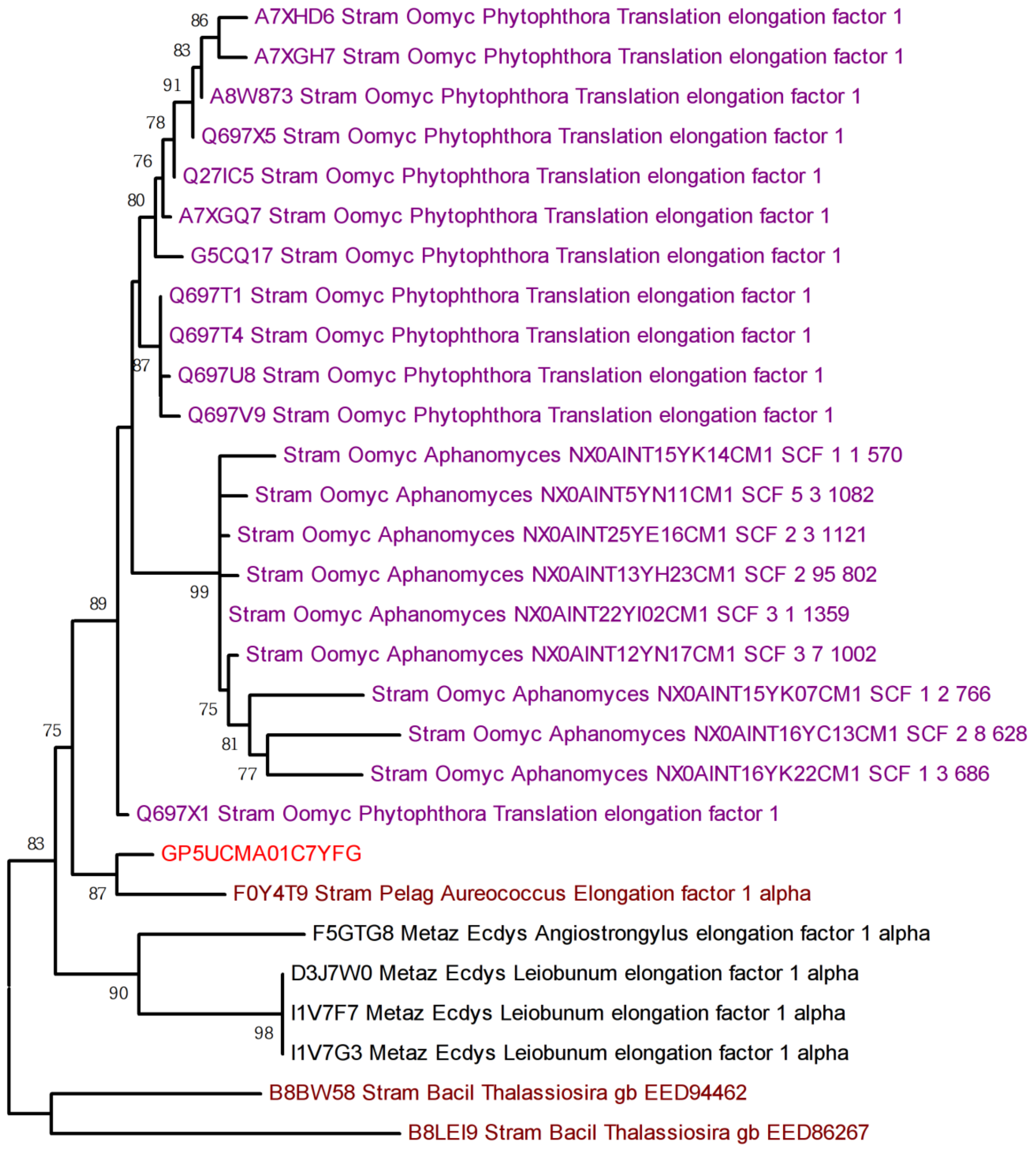

Figure S7-7. Translation elongation factor 1-alpha (GP5UCMA01C7YFG).

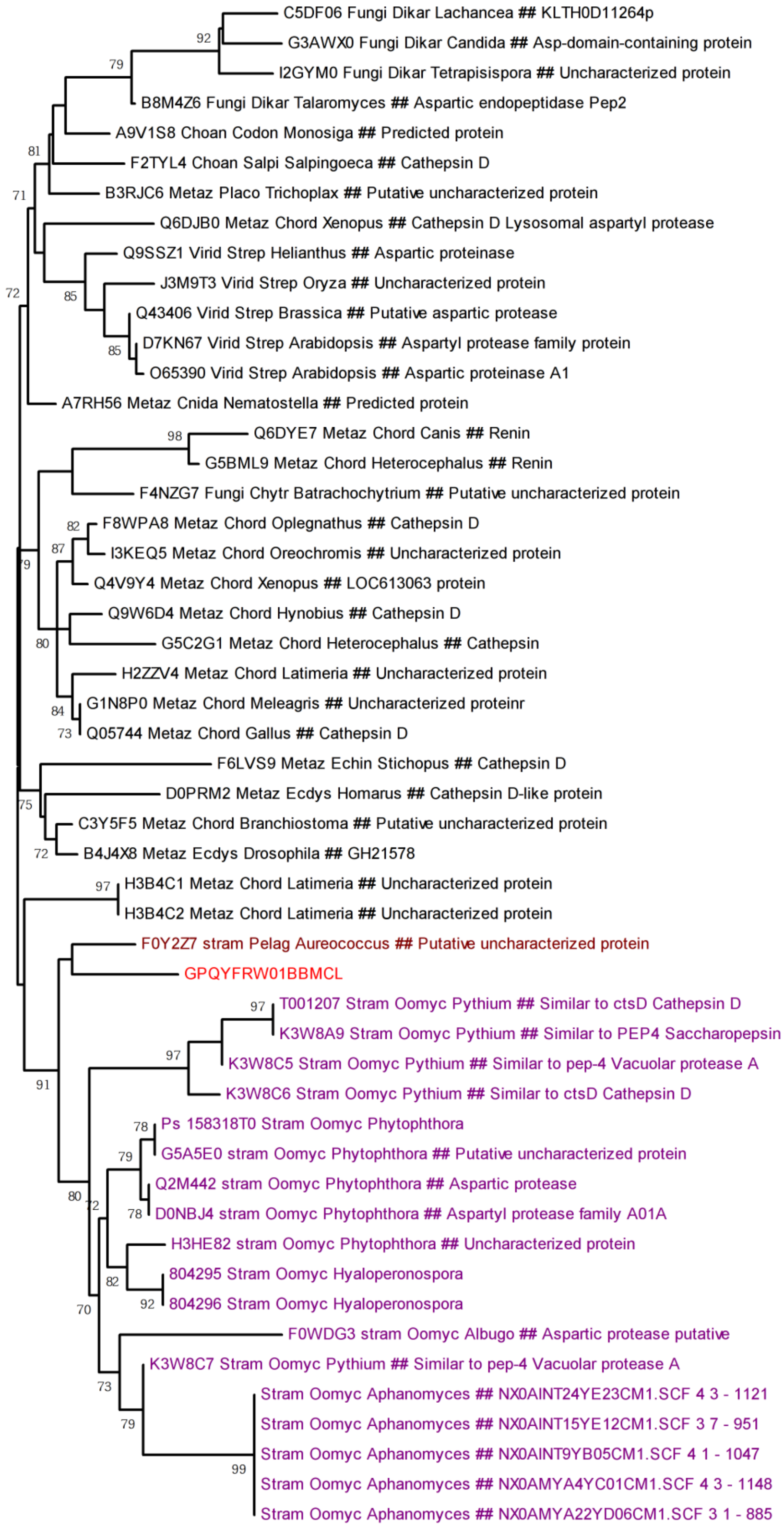

Figure S7-8. Aspartic proteinases (GPQYFRW01BBMCL).

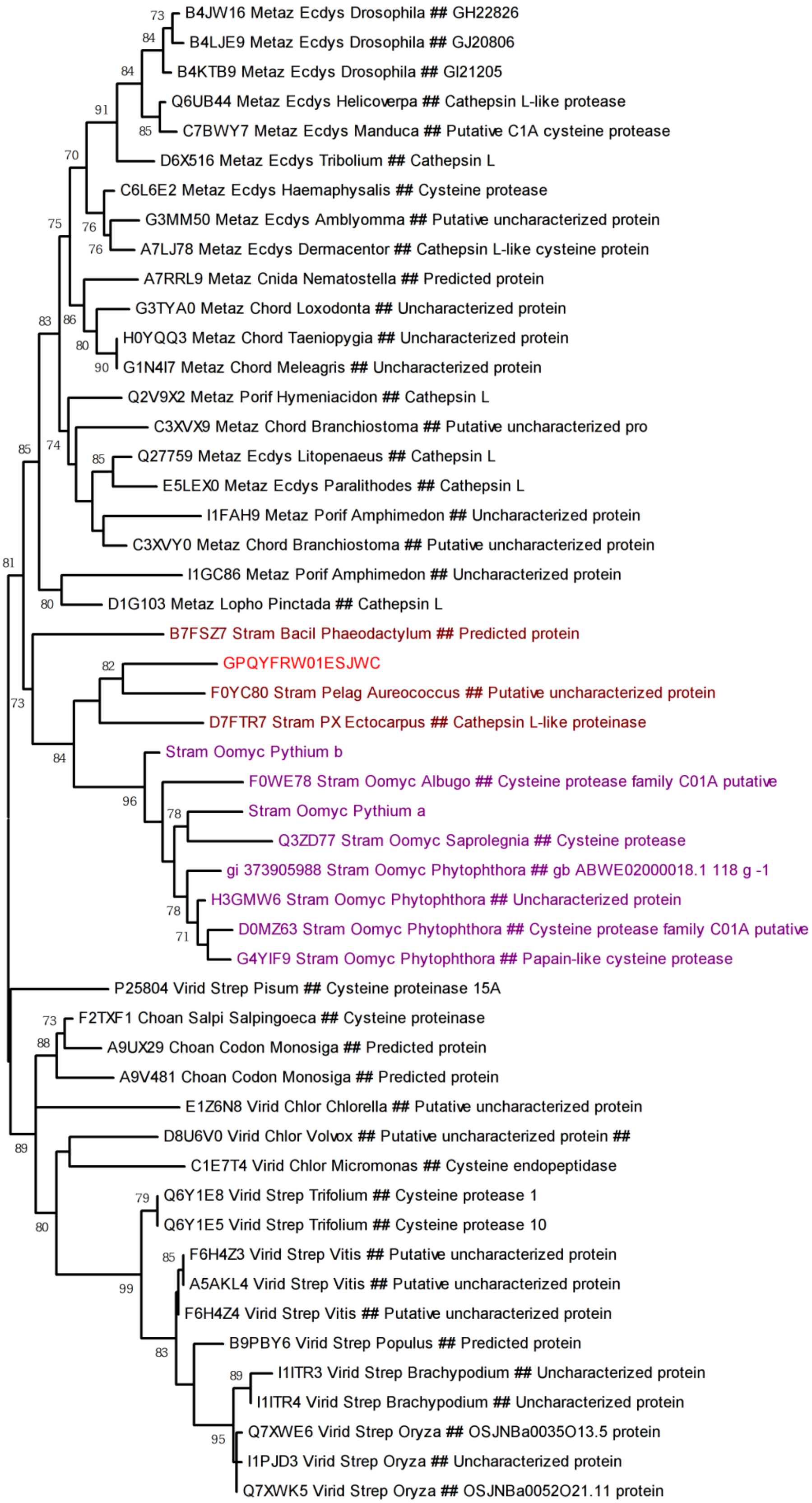

Figure S7-9. Cysteine protease homologs (GPQYFRW01ESJWC).

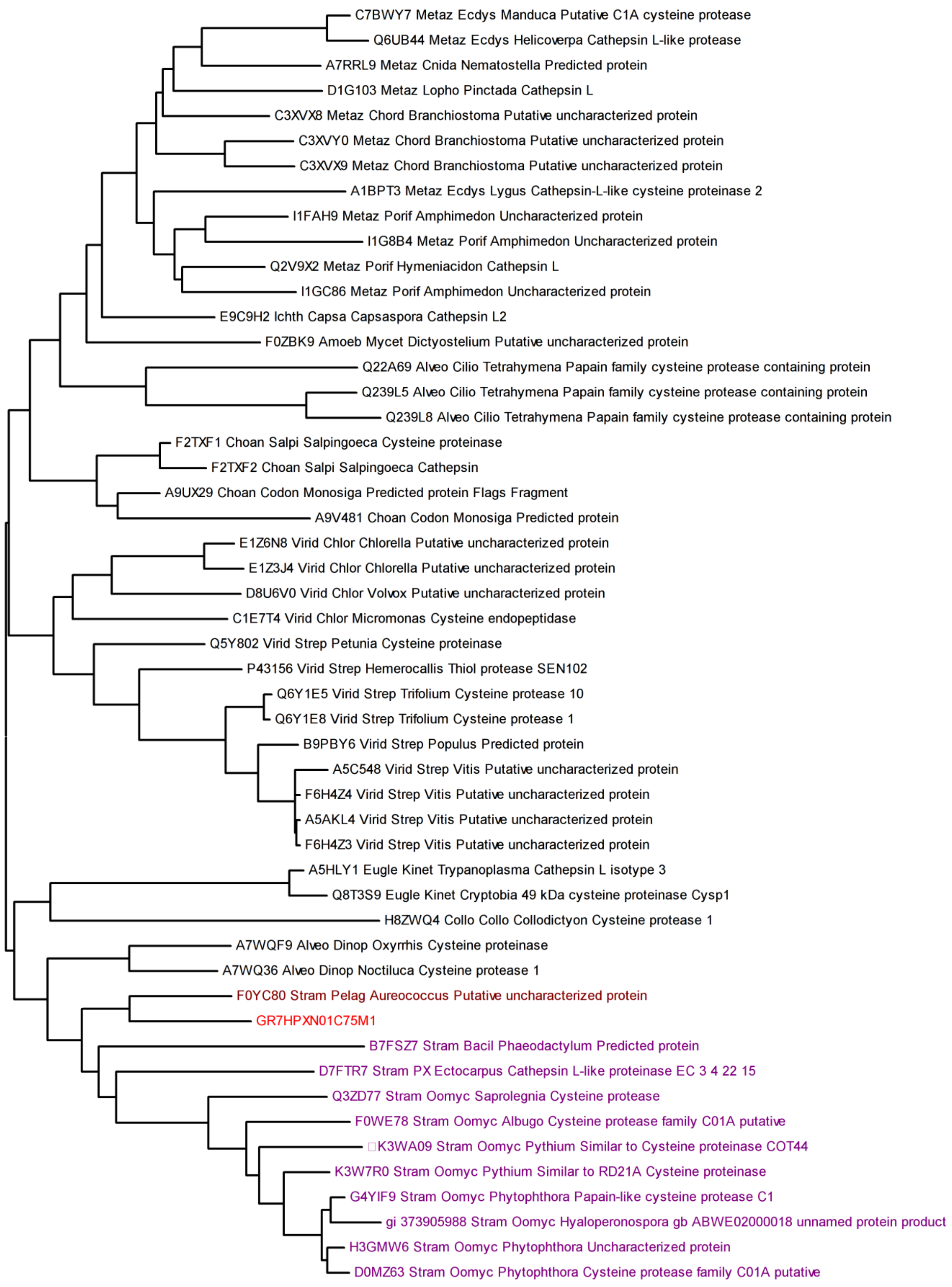

Figure S7-10. Cysteine protease homologs (GR7HPXN01C75M1). The phylogenetic analysis was performed using Pplacer on a reference tree built by PhyML.

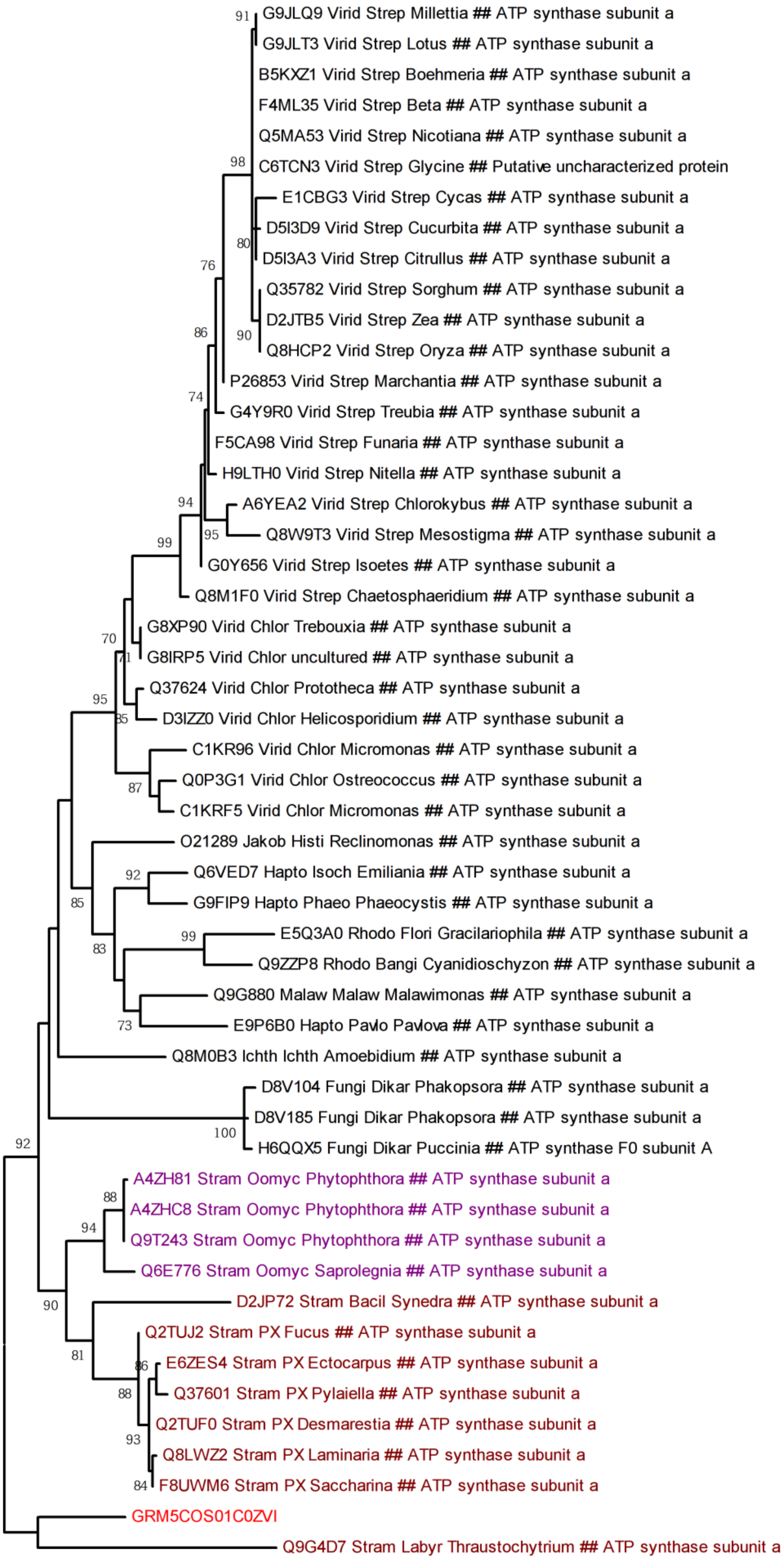

Figure S7-11. ATP synthase subunit a (GRM5COS01C0ZVI).

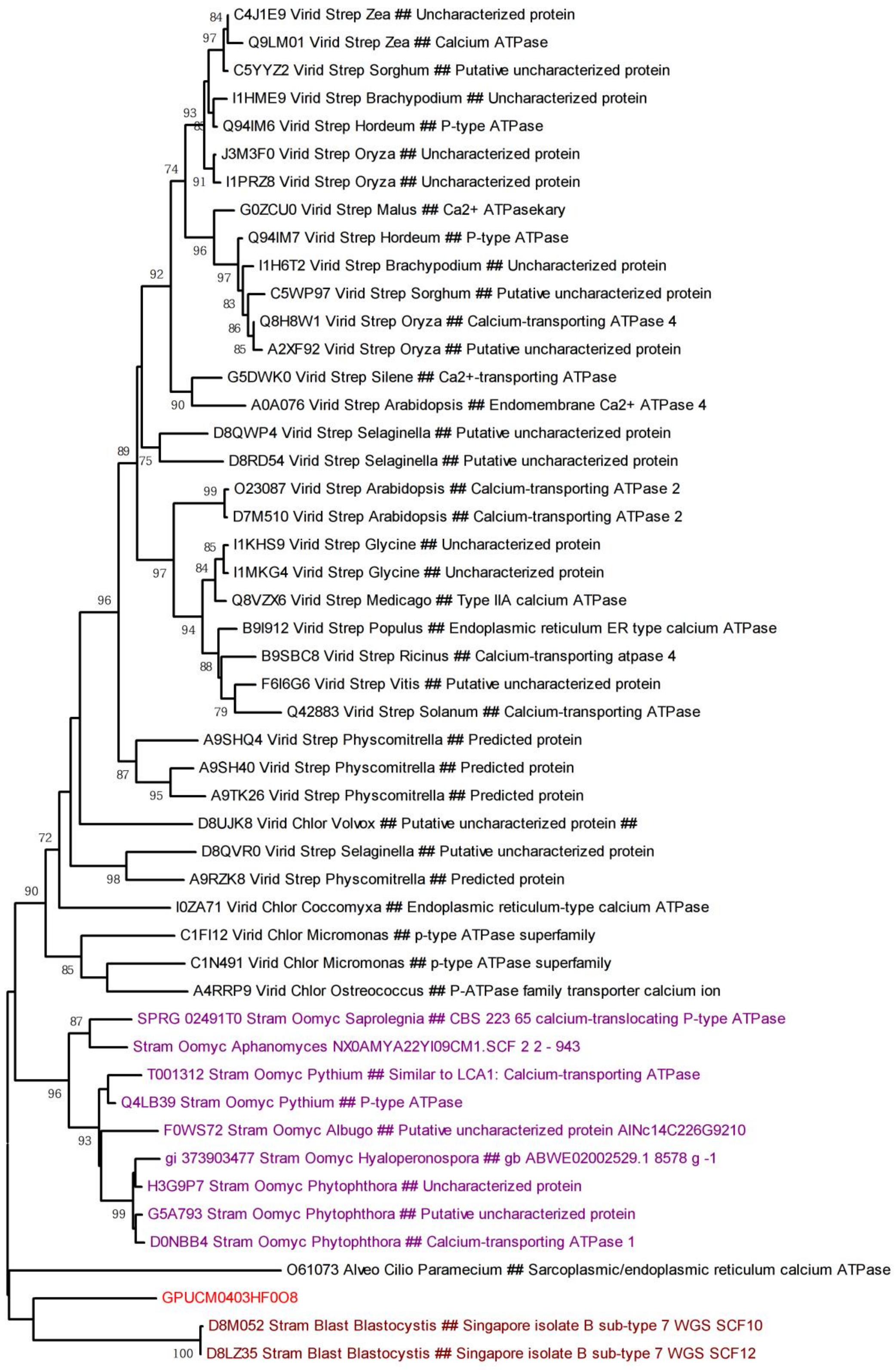

Figure S7-12. Calcium transporting ATPase (P-type ATPase) (GPUCM0403HF0O8).

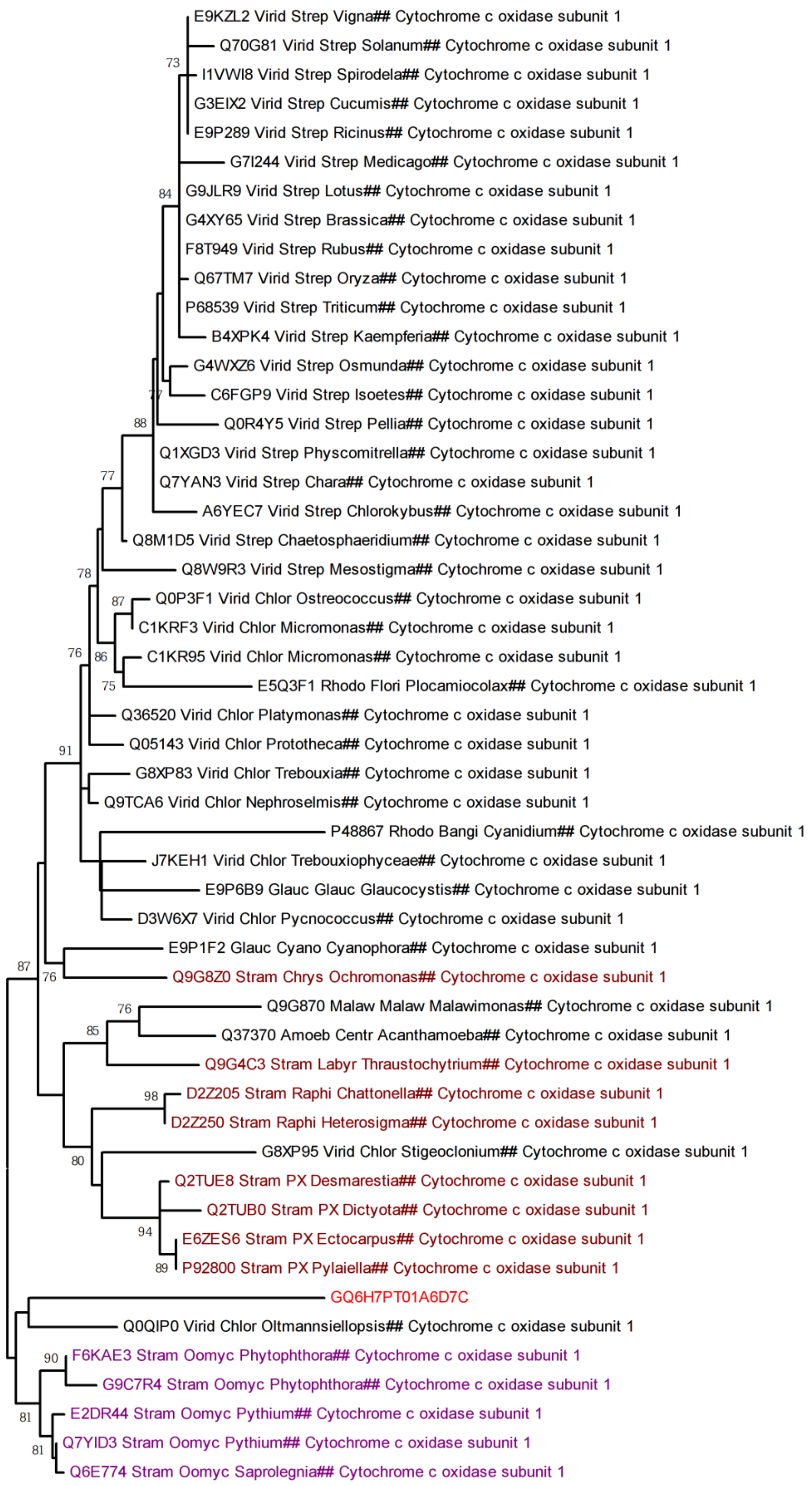

Figure S7-13. Cytochrome c oxidase subunit 1 (GQ6H7PT01A6D7C).

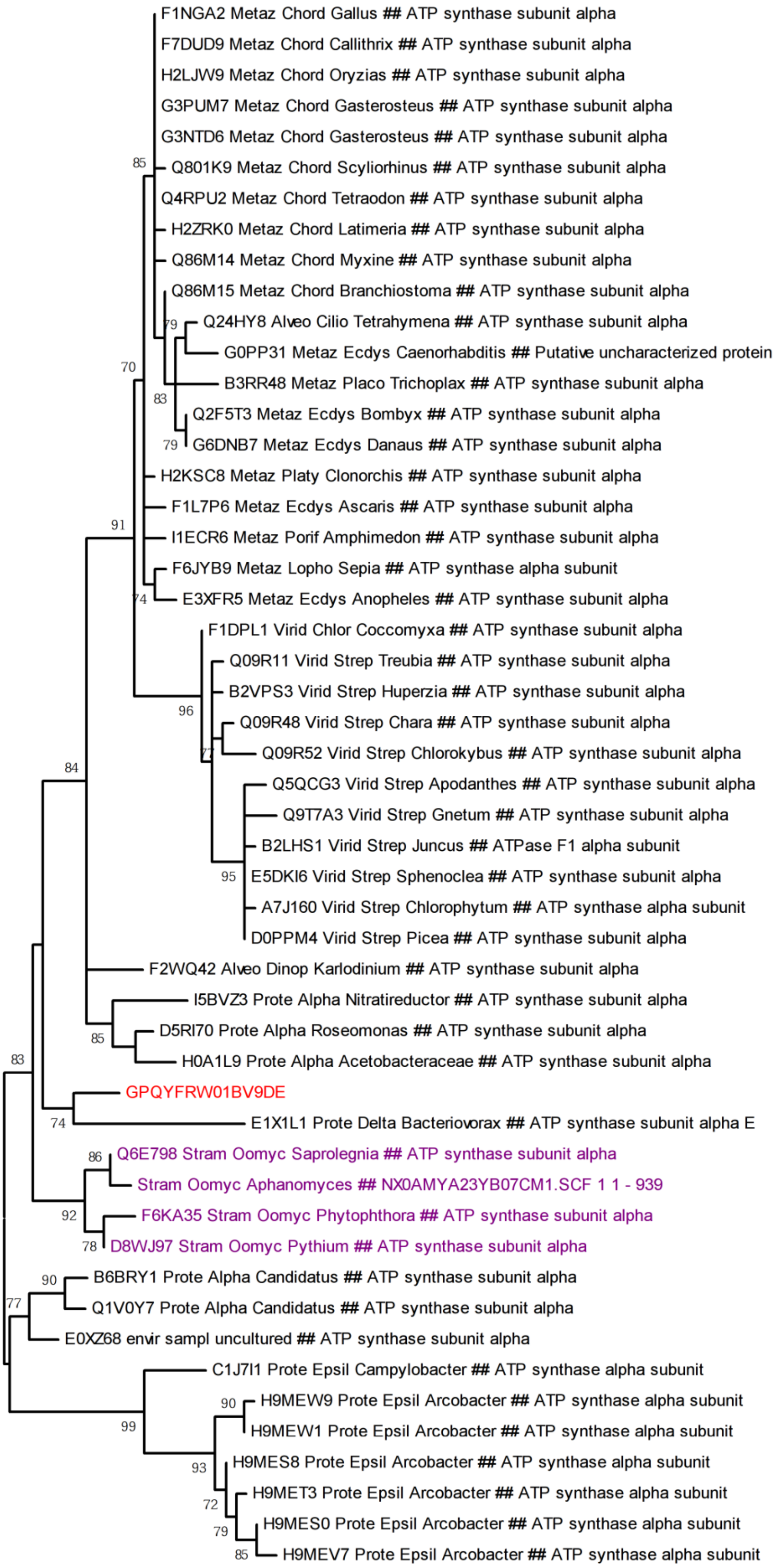

Figure S7-14. ATP synthase subunit alpha (GPQYFRW01BV9DE).

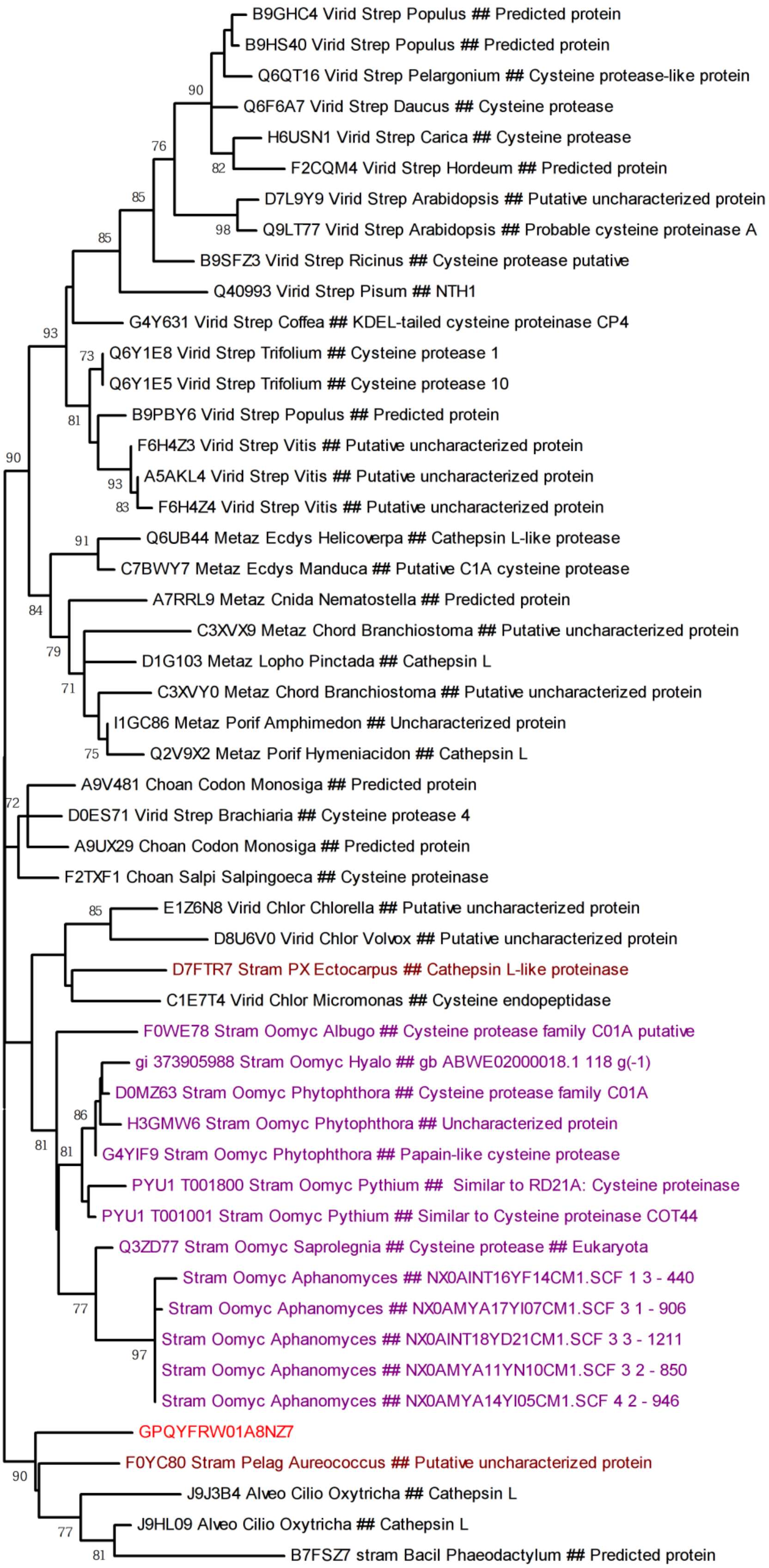

Figure S7-15. Cysteine protease homologs (GPQYFRW01A8NZ7).

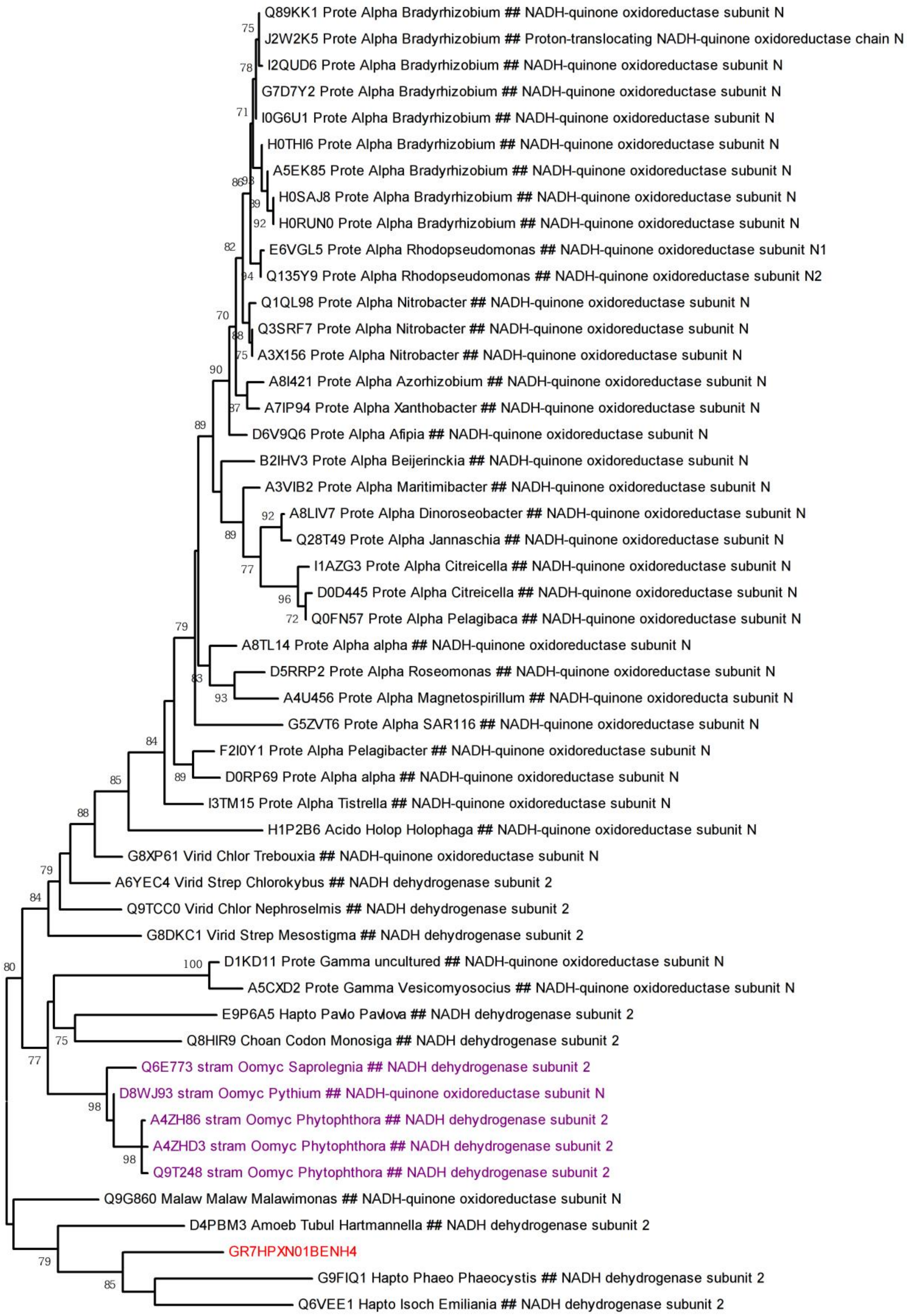

Figure S7-16. NADH dehydrogenase subunit 2 (GR7HPXN01BENH4).

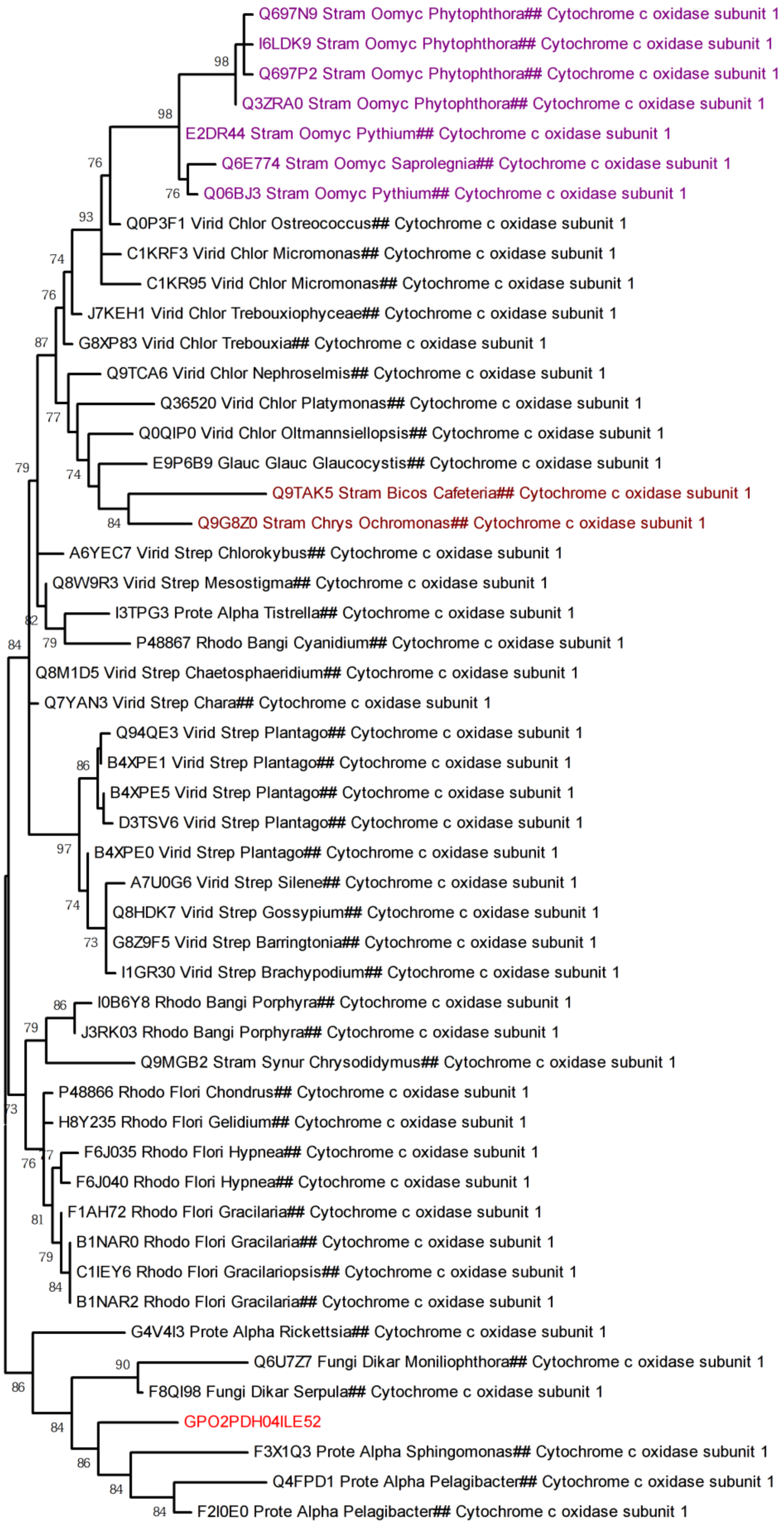

Figure S7-17. Cytochrome c oxidase subunit 1 (GPO2PDH04ILE52).

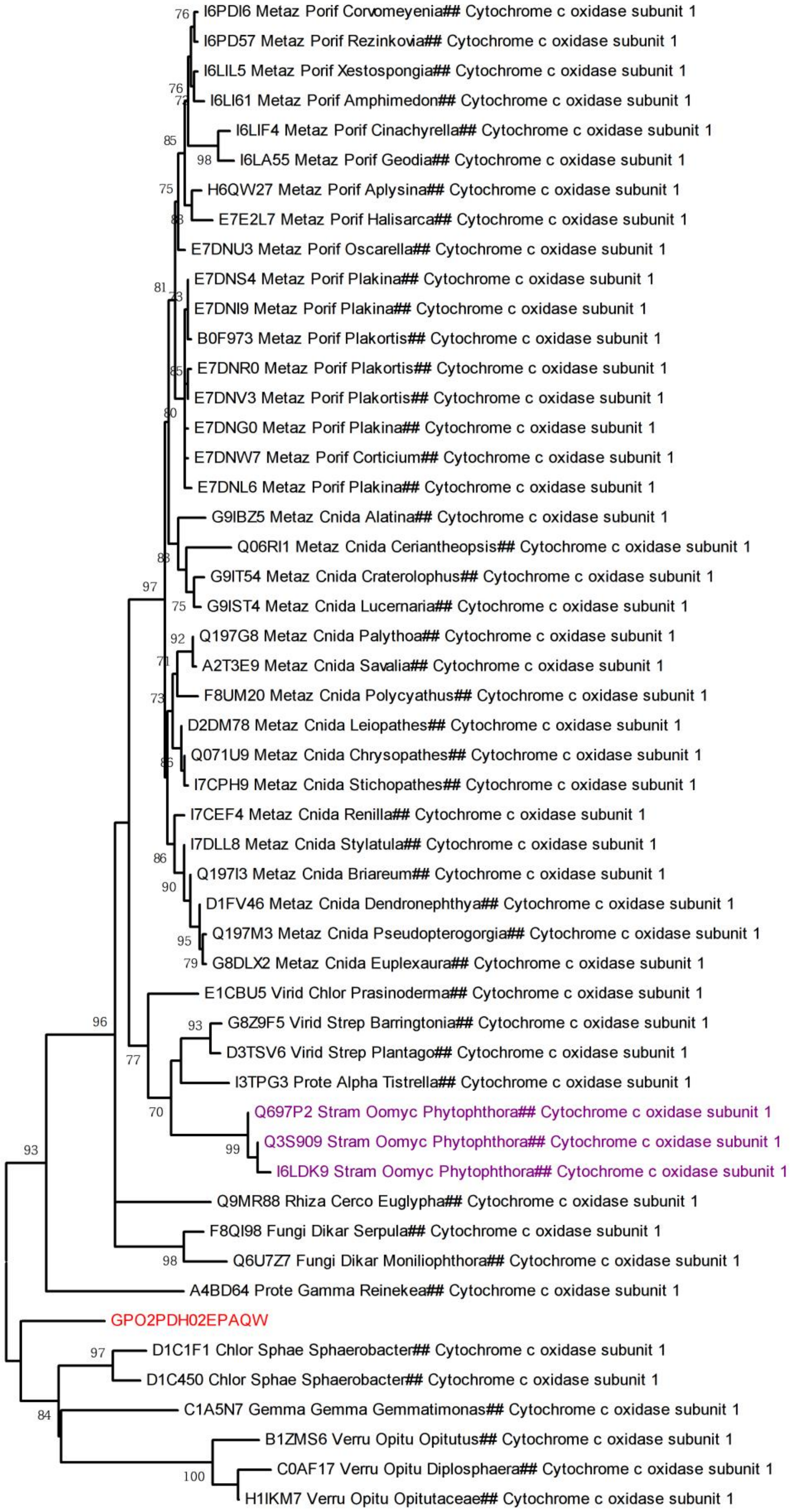

Figure S7-18. Cytochrome c oxidase subunit 1 (GPO2PDH02EPAQW).

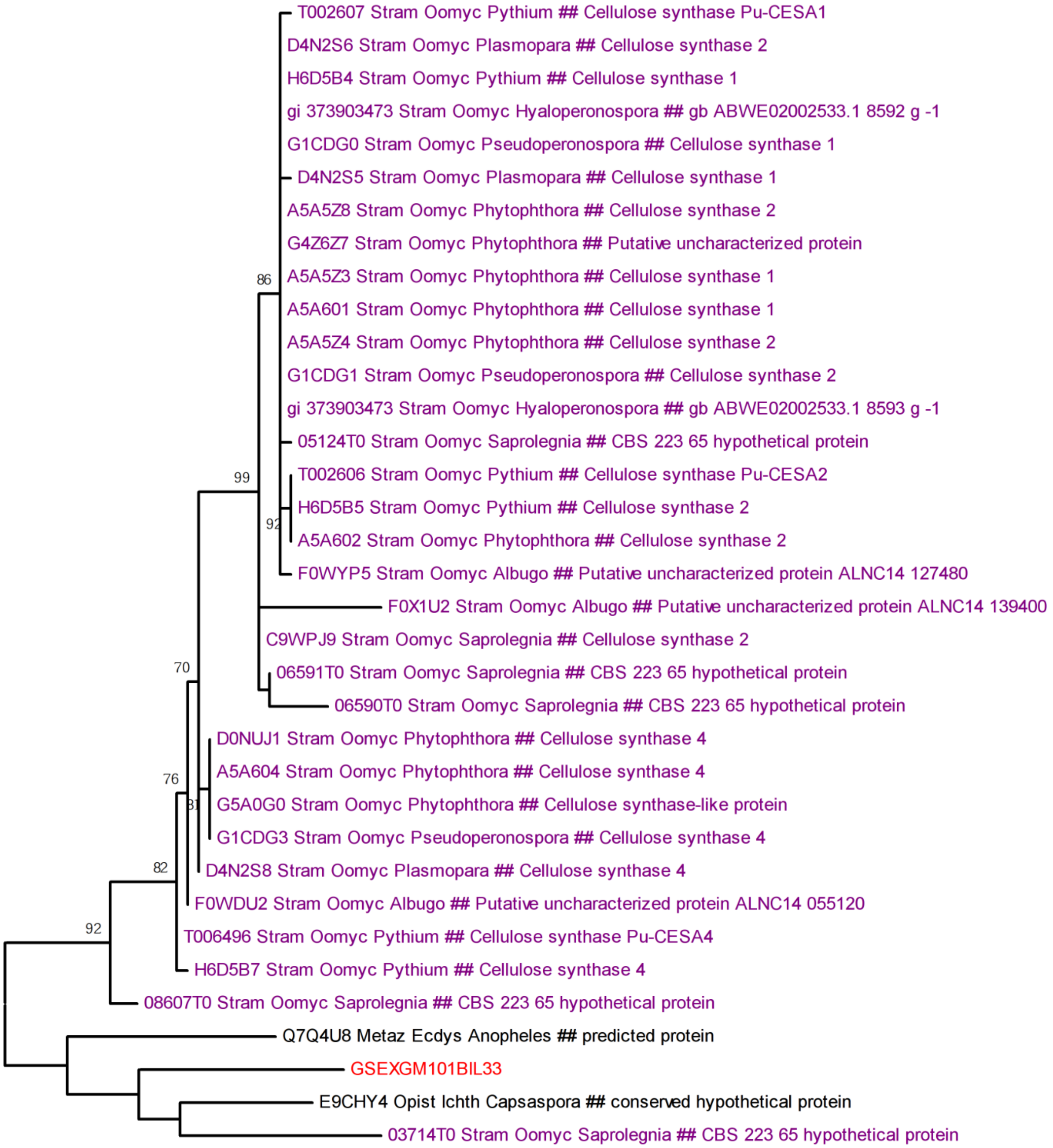

Figure S7-19. Cellulose synthase (GSEXGM101BIL33).

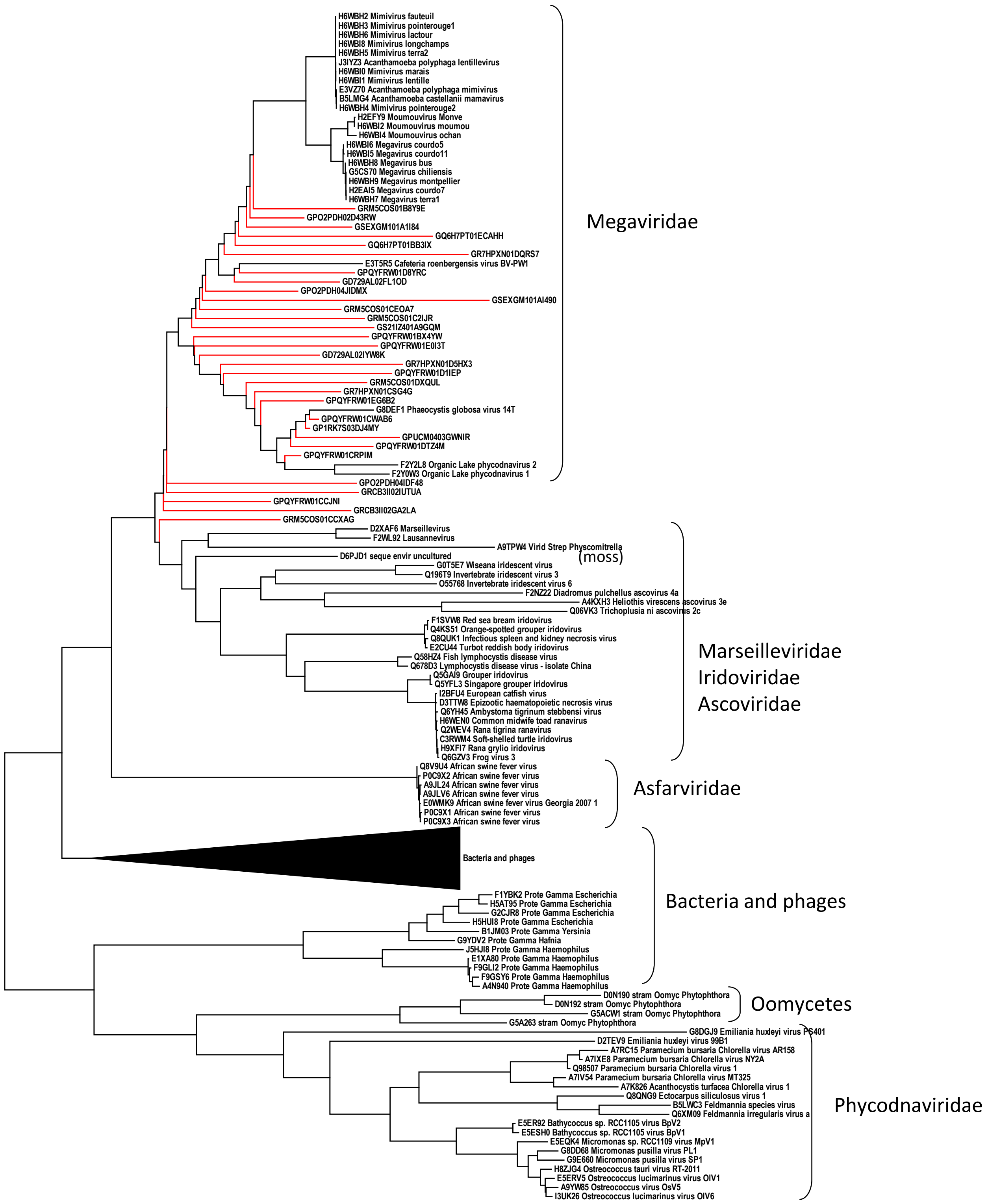

Figure S8. Phylogenetic analysis of 31 reads similar to predicted primases/helicasses (D5 family proteins). Tara Oceans peptides were mapped on a reference tree of D5 family proteins by Pplacer. They are highlighted by red branches. This tree is an superposition of 31 independent phylogenetic mapping results and the phylogenetic relationships among Tara Oceans peptides are not considered in the tree calculation. Therefore, only the position of each read (red) relative to the reference tree (black backbone branches) are meaningful. Most of the reads were classified within the Megaviridae clade. We noted that oomycetes have homologs related to Phycodnaviridae sequences. The moss *Physcomitrella* placed near Marseillevirus is known for its acquisition of viral genes by HGT (Yeu et al., Nat. Commun., 2012).

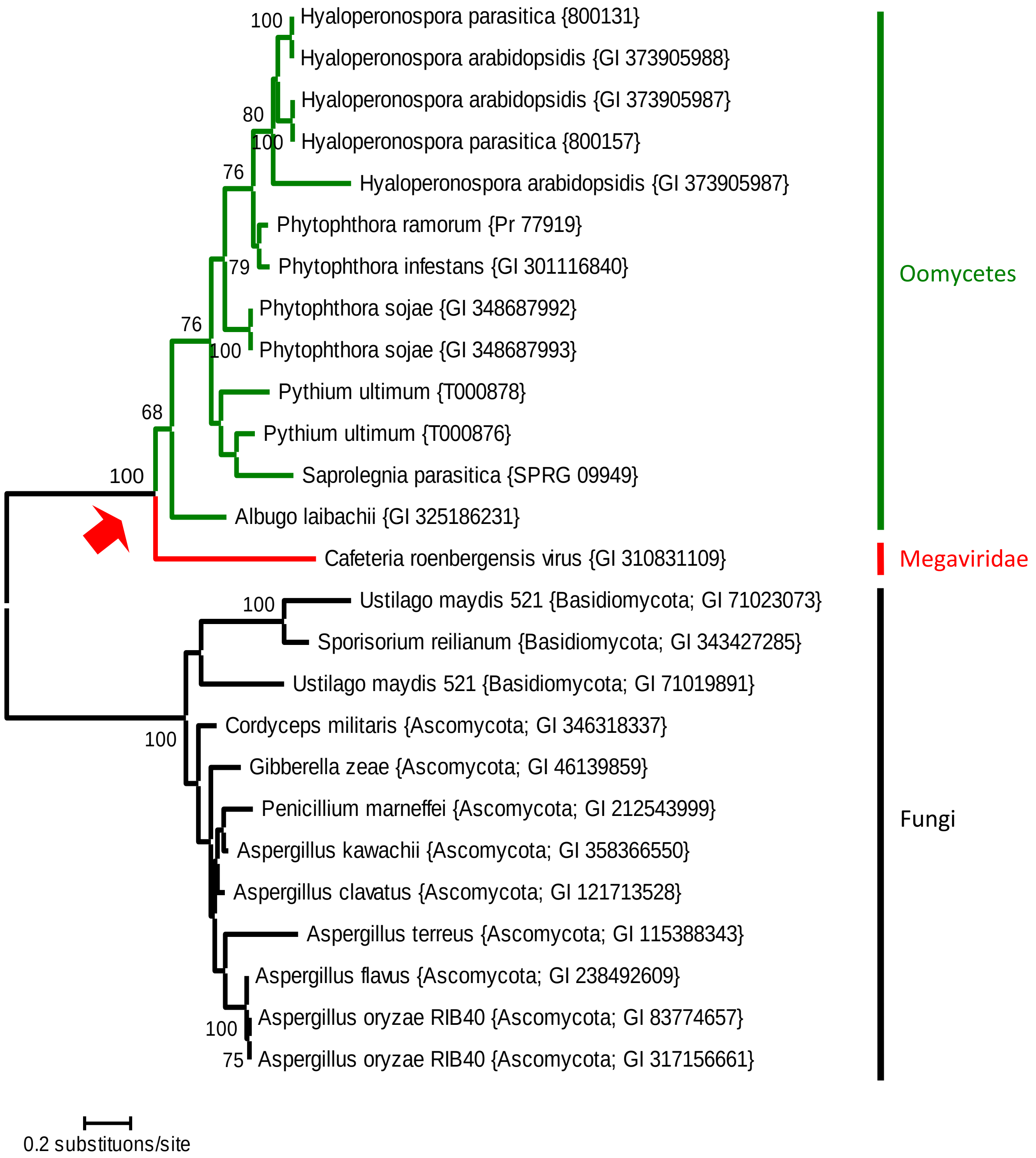

Figure S9. Evidence of horizontal gene transfer between the Megaviridae and Oomycete lineages. The phylogenetic trees were generated using PhyML based on protein sequences. The numbers on the branches indicate bootstrap percentages after 100 bootstrap sampling and the trees were mid-point rooted for visualization purpose. **(S9-1)** ML-tree based on the protein sequences of the CroV putative fatty acid hydroxylase (gi: 310831109) and its homologs.

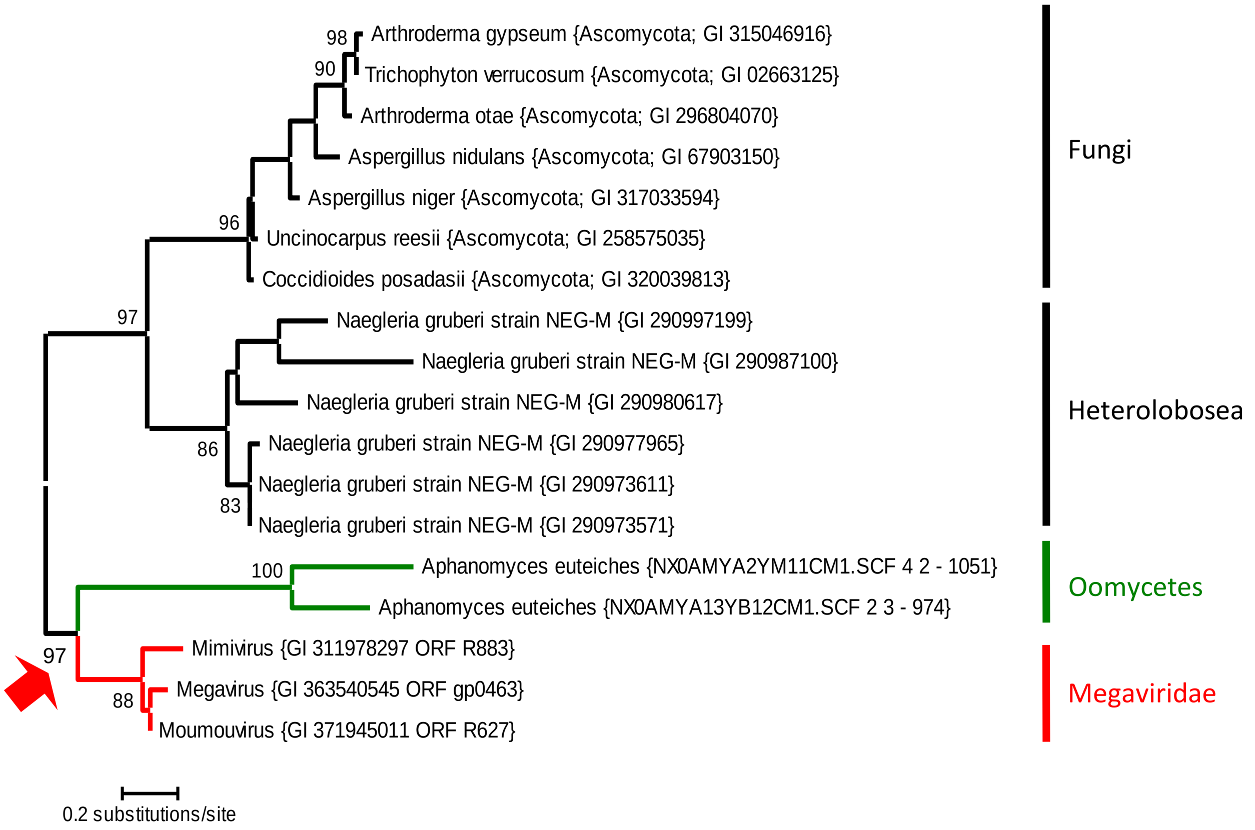

Figure S9-2. ML-tree based on the protein sequences of the Moumouvirus hypothetical protein (gi: 371945011) and its homologs.

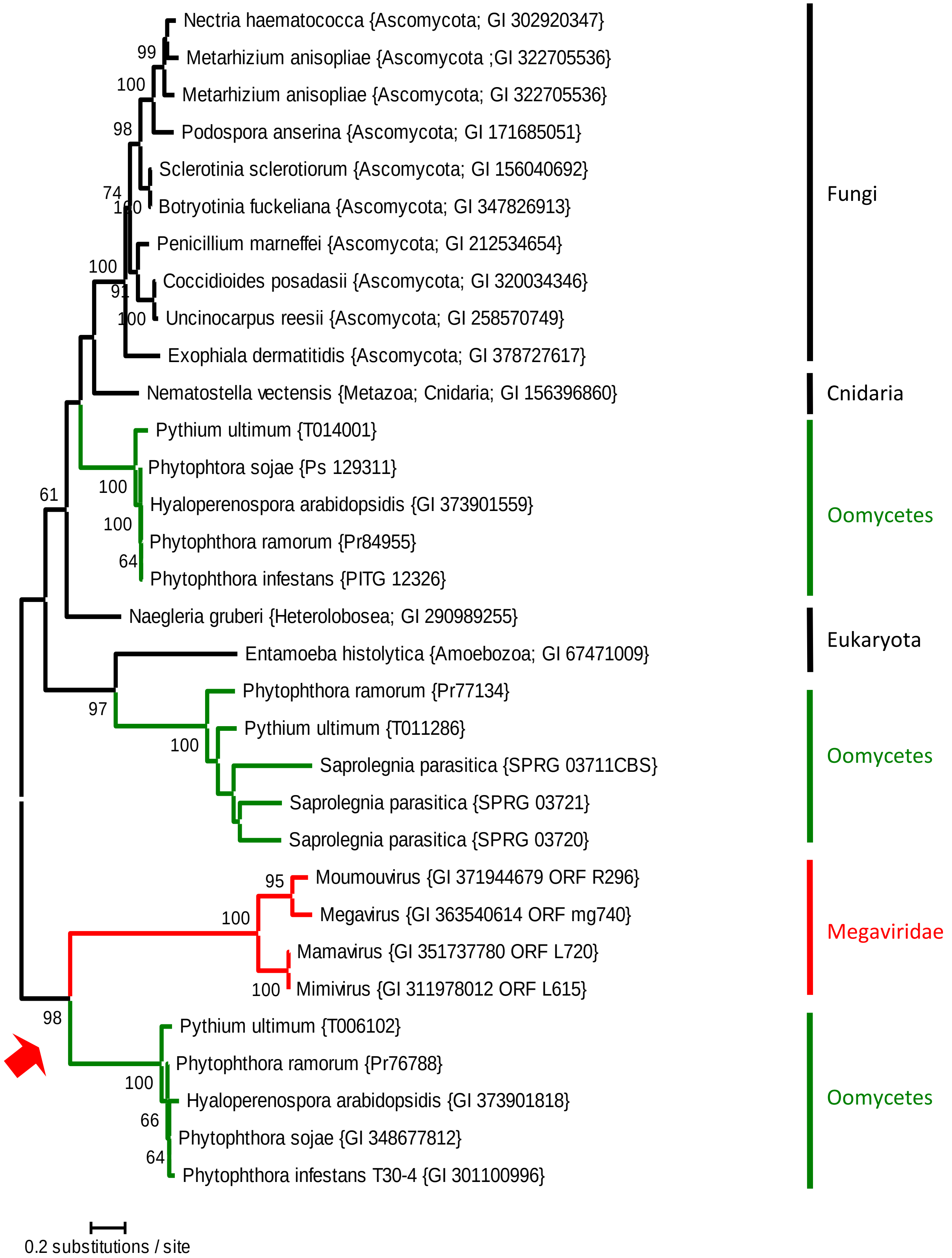

Figure S9-3. ML-tree based on the protein sequences of the Mimivirus putative phosphatidylinositol kinase (AEJ34862) and its homologs.

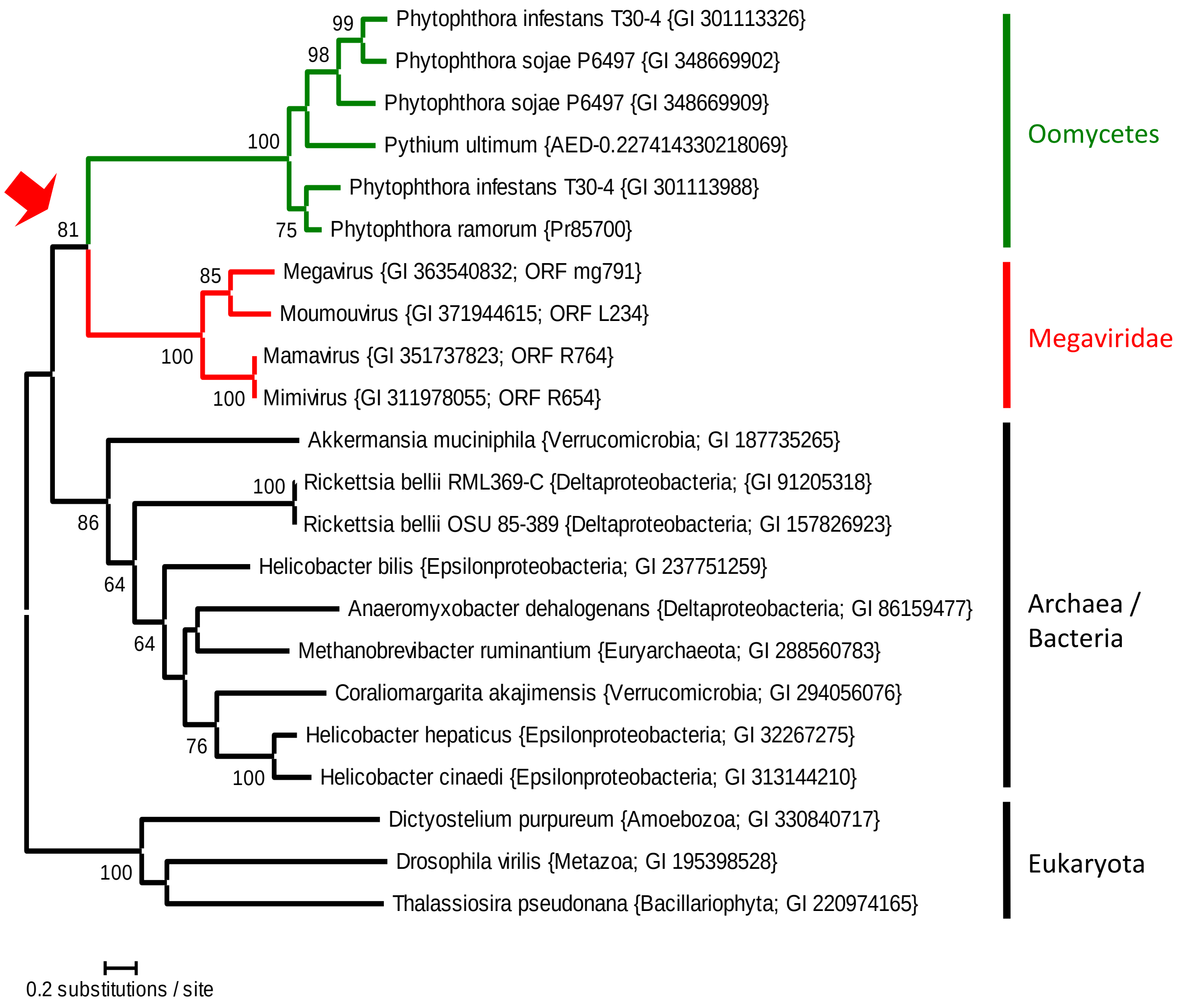

Figure S9-4. ML-tree based on the protein sequences of the Mimivirus putative fucosyltransferase (AEJ34901) and its homologs.

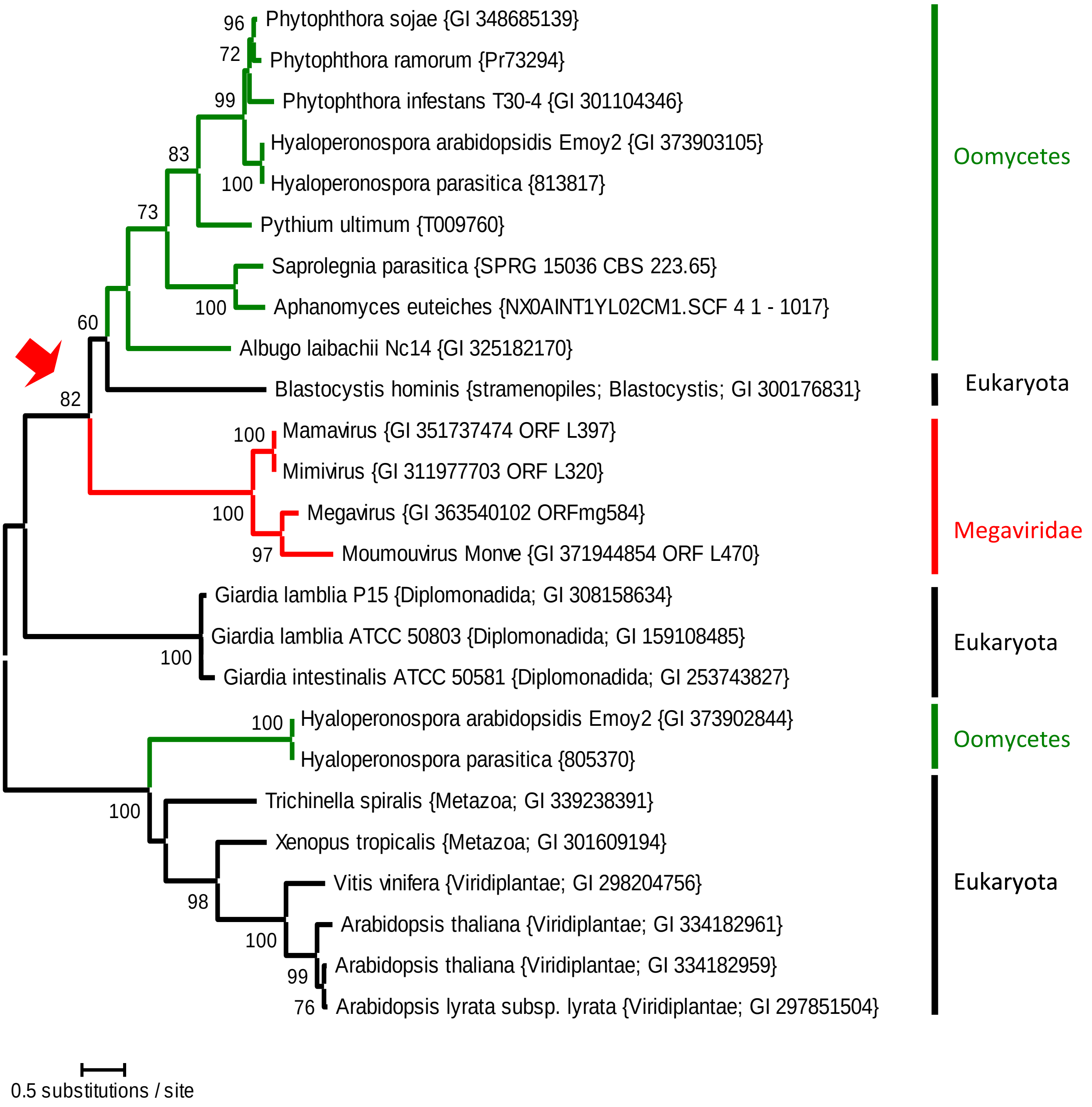

Figure S9-5. ML-tree based on the protein sequences of the Mimivirus putative RNA methylase (S-adenosyl-L-Methionine-dependent methyltransferase; gi: 311977703) and its homologs.
