# Supplementary material for: Exploring nucleo-cytoplasmic large DNA viruses in Tara Oceans microbial metagenomes
Source: ISME J. 2013 Apr 11;7(9):1678–95. doi: 10.1038/ismej.2013.59 (PMC3749498; doi:10.1038/ismej.2013.59)
Supplement: Supplementary Table S1 [file ismej201359x2.pdf]

**Supplementary Table S1.** NCLDV marker genes.

| Identifier | Description | Distribution of homologs (number of proteins/number of genomes) | | | | |
|---|---|---|---|---|---|---|
| | | NCLDV [a] (59) | Megaviridae [a] (3) | Phycodna-viridae [a] (14) | Non-NCLDV viruses [a] (3726) | Cellular organisms [a] (1439) |
| | **Conserved in most NCLDVs & nearly single copy** | | | | | |
| NCVOG1164 [b] | A1L transcription factor (Late transcription factor VLTF2 like) | 58/58 | 3/3 | 14/14 | 0/0 | 1/1 |
| NCVOG0262 [b] | A2L transcription factor (Late transcription factor VLTF3 like) | 60/59 | 4/3 | 14/14 | 0/0 | 1/1 |
| NCVOG0249 | A32-like DNA packaging ATPase | 63/59 | 6/3 | 15/14 | 5/4 | 1625/95 |
| NCVOG0038 | DNA polymerase family B | 60/58 | 4/3 | 15/14 | 179/168 | 1133/495 |
| | **Conserved in most Megaviridae and Phycodnaviridae & nearly single copy** | | | | | |
| NCVOG0158 [b] | Hypothetical conserved protein | 28/28 | 3/3 | 14/14 | 0/0 | 1/1 |
| NCVOG1137 [b] | Hypothetical conserved protein | 17/16 | 4/3 | 13/13 | 0/0 | 1/1 |
| NCVOG1342 [b] | Hypothetical conserved protein | 16/16 | 3/3 | 13/13 | 0/0 | 3/2 |
| NCVOG1129 [b] | Hypothetical conserved protein | 14/14 | 3/3 | 11/11 | 0/0 | 0/0 |
| NCVOG1216 [b] | Hypothetical conserved protein | 14/14 | 3/3 | 11/11 | 0/0 | 0/0 |
| NCVOG0276 | Ribonucleotide reductase small subunit | 49/49 | 3/3 | 14/14 | 139/138 | 1396/1075 |
| NCVOG1353 | Ribonucleotide reductase large subunit | 37/36 | 4/3 | 14/14 | 174/167 | 1812/1304 |
| NCVOG0278 | Holliday junction resolvases (RuvC) | 54/50 | 3/3 | 17/13 | 0/0 | 35/34 |
| NCVOG1354 | Ribonuclease III | 30/28 | 3/3 | 11/11 | 0/0 | 1513/1264 |
| NCVOG0313 | TATA box binding protein (TBP) | 15/15 | 3/3 | 11/11 | 0/0 | 267/211 |
| NCVOG1127 | Transcription initiation factor IIB | 18/18 | 3/3 | 11/11 | 0/0 | 610/261 |
| NCVOG1166 | Poxvirus C4/C10 protein (Prolyl 4-hydroxylase alpha subunit homologues) | 48/31 | 3/3 | 11/11 | 7/6 | 452/142 |

***a***: The number of genomes analyzed is shown in parentheses. Viruses included in Megaviridae are Mimivirus, Megavirus and CroV.
***b***: Seven NCLDV marker genes that are nearly absent in non-NCLDV viruses and cells.
