# Supplementary material for: Exploring nucleo-cytoplasmic large DNA viruses in Tara Oceans microbial metagenomes
Source: ISME J. 2013 Apr 11;7(9):1678–95. doi: 10.1038/ismej.2013.59 (PMC3749498; doi:10.1038/ismej.2013.59)
Supplement: Supplementary Table S2 [file ismej201359x3.pdf]

Supplementary Table S2. Source of oomycete genomic/transcriptomic sequences.

Species	Databases
Aphanomyces euteiches ESTs	AphanoDB: http://www.polebio.scsv.ups-tlse.fr/aphano/download/EST_db.fasta
Hyaloperonospora arabidopsidis	NCBI: ftp://ftp.ncbi.nlm.nih.gov/genbank/wgs/wgs.ABWE.1.fsa_nt.gz
Pythium ultimum	Pythium Genome Database: http://pythium.plantbiology.msu.edu/data/pythium_ultimum_proteins.fasta.zip
Hyaloperonospora parasitica, Phytophthora infestans, Phytophthora ramorum, Phytophthora sojae, Saprolegnia parasitica	The Broad Institute of Harvard and MIT “Saprolegnia and Phytophthora Sequencing Project”: http://www.broadinstitute.org/
Thalassiosira pseudonana	NCBI: http://www.ncbi.nlm.nih.gov/genome/?term=txid296543
