# Supplementary material for: Exploring nucleo-cytoplasmic large DNA viruses in Tara Oceans microbial metagenomes
Source: ISME J. 2013 Apr 11;7(9):1678–95. doi: 10.1038/ismej.2013.59 (PMC3749498; doi:10.1038/ismej.2013.59)
Supplement: Supplementary Table S4 [file ismej201359x5.pdf]

Supplementary Table S4. Summary of phylogenetic analyses of the metagenome reads assigned to "oomycetes" by 2bLCA.

Figure no.	Read ID	Definition of homologous sequences	Phylogenetic analysis method	Close homologs
Figure S7-1	GPO2PDH04IVTOL	Cellulose synthase	PhyML	Stramenopile (related to oomycetes)
Figure S7-2	GPQYFRW01E1K4R	Geranylgeranyl diphosphage synthase	PhyML	Stramenopile (related to oomycetes)
Figure S7-3	GRO00XQ01B0IV4	Pyruvate kinase	PhyML	Stramenopile (related to oomycetes)
Figure S7-4	GRM5COS01AKC1A	Cysteine protease; Cathepsin L-like proteinase	PhyML	Stramenopile (related to oomycetes)
Figure S7-5	GD729AL02GZ44E	Cytochrome b	PhyML	Stramenopile (related to oomycetes)
Figure S7-6	GD729AL02HLFO1	Cytochrome c oxidase subunit 1	PhyML	Stramenopile (related to oomycetes)
Figure S7-7	GP5UCMA01C7YFG	Translation elongation factor 1-alpha	PhyML	Stramenopile (related to Aureococcus)
Figure S7-8	GPQYFRW01BBMCL	Aspartic proteinases	PhyML	Stramenopile (related to Aureococcus)
Figure S7-9	GPQYFRW01ESJWC	Cysteine protease; Cathepsin L-like proteinase	PhyML	Stramenopile (related to Aureococcus)
Figure S7-10	GR7HPXN01C75M1	Cysteine protease; Cathepsin L-like proteinase	PhyML+Pplacer	Stramenopile (related to Aureococcus)
Figure S7-11	GRM5COS01C0ZVI	ATP synthase subunit a	PhyML	Stramenopile (related to Thraustochytrium)
Figure S7-12	GPUCM0403HF0O8	Calcium transporting ATPase (P-type ATPase)	PhyML	Stramenopile (related to Blastocystis)
Figure S7-13	GQ6H7PT01A6D7C	Cytochrome c oxidase subunit 1	PhyML	Viridiplantae (related to Oltmannsiellopsis)
Figure S7-14	GPQYFRW01BV9DE	ATP synthase subunit alpha	PhyML	Proteobacteria (related to Bacteriovorax)
Figure S7-15	GPQYFRW01A8NZ7	Cysteine protease; Cathepsin L-like proteinase	PhyML	Stramenopile, Alveolata
Figure S7-16	GR7HPXN01BENH4	NADH dehydrogenase subunit 2	PhyML	Haptophyte
Figure S7-17	GPO2PDH04ILE52	Cytochrome c oxidase subunit 1	PhyML	Proteobacteria (Pelagibacter)
Figure S7-18	GPO2PDH02EPAQW	Cytochrome c oxidase subunit 1	PhyML	Chloroflexi, Verrucomicrobia
Figure S7-19	GSEXGM101BIL33	Cellulose synthase	PhyML	Stramenopile, Opisthokonta
