# Supplementary material for: Exploring nucleo-cytoplasmic large DNA viruses in Tara Oceans microbial metagenomes
Source: ISME J. 2013 Apr 11;7(9):1678–95. doi: 10.1038/ismej.2013.59 (PMC3749498; doi:10.1038/ismej.2013.59)
Supplement: Supplementary Table S5 [file ismej201359x6.pdf]

Supplementary Table S5. List of HGT candidates obtained by reciprocal BLAST search.

Mimiviridae proteins		Cellular Reciprocal Best BLAST Hit	
Viruses; dsDNA viruses, no RNA stage; Mimiviridae;	crov535	Archaea; Euryarchaeota; Methanomicrobia; Methanosarcinales; Methanosarcinaceae; Methanosarcina;	lcl|UniRef100_Q8PUY7
Viruses; dsDNA viruses, no RNA stage; Mimiviridae;	crov242	Bacteria; Bacteroidetes; Bacteroidia; Bacteroidales; Bacteroidaceae; Bacteroides;	lcl|UniRef100_C3R4Q1
Viruses; dsDNA viruses, no RNA stage; Mimiviridae;	crov478	Bacteria; Chlamydiae; Chlamydiales; Waddliaceae; Waddlia;	lcl|UniRef100_F8LFE4
Viruses; dsDNA viruses, no RNA stage; Mimiviridae;	crov269	Bacteria; Cyanobacteria; Chroococcales; Synechococcus;	lcl|UniRef100_UPI00020024B3
Viruses; dsDNA viruses, no RNA stage; Mimiviridae;	crov011	Bacteria; environmental samples;	lcl|UniRef100_G8DB35
Viruses; dsDNA viruses, no RNA stage; Mimiviridae;	crov224	Bacteria; Proteobacteria; environmental samples;	lcl|UniRef100_UPI0001E7AE94
Viruses; dsDNA viruses, no RNA stage; Mimiviridae;	crov237	Bacteria; Proteobacteria; environmental samples;	lcl|UniRef100_UPI0001924CDF
Viruses; dsDNA viruses, no RNA stage; Mimiviridae;	crov325	Bacteria; Proteobacteria; environmental samples;	lcl|UniRef100_UPI0001E7AE96
Viruses; dsDNA viruses, no RNA stage; Mimiviridae;	crov454	Bacteria; Proteobacteria; environmental samples;	lcl|UniRef100_UPI0001E7AE98
Viruses; dsDNA viruses, no RNA stage; Mimiviridae;	crov497	Bacteria; Proteobacteria; environmental samples;	lcl|UniRef100_UPI0001E7AE9A
Viruses; dsDNA viruses, no RNA stage; Mimiviridae;	crov349	Bacteria; Proteobacteria; Gammaproteobacteria; Alteromonadales; Alteromonadaceae; Saccharophagus;	lcl|UniRef100_Q21EC4
Viruses; dsDNA viruses, no RNA stage; Mimiviridae;	crov503	Bacteria; Tenericutes; Mollicutes; Mycoplasmataceae; Mycoplasma;	lcl|UniRef100_F9UKI1
Viruses; dsDNA viruses, no RNA stage; Mimiviridae;	crov149	Eukaryota; Alveolata; Ciliophora; Intramacronucleata; Oligohymenophorea; Peniculida; Parameciidae; Paramecium;	lcl|UniRef100_A0DL57
Viruses; dsDNA viruses, no RNA stage; Mimiviridae;	crov002	Eukaryota; Amoebozoa; Mycetozoa; Dictyosteliida; Dictyostelium;	lcl|UniRef100_F0Z9X5
Viruses; dsDNA viruses, no RNA stage; Mimiviridae;	crov031	Eukaryota; Amoebozoa; Mycetozoa; Dictyosteliida; Dictyostelium;	lcl|UniRef100_F0ZM65
Viruses; dsDNA viruses, no RNA stage; Mimiviridae;	crov527	Eukaryota; Amoebozoa; Mycetozoa; Dictyosteliida; Dictyostelium;	lcl|UniRef100_F0ZPF1
Viruses; dsDNA viruses, no RNA stage; Mimiviridae;	UniRef100_Q5UQ73	Eukaryota; Amoebozoa; Mycetozoa; Dictyosteliida; Dictyostelium;	lcl|UniRef100_F0ZTJ7
Viruses; dsDNA viruses, no RNA stage; Mimiviridae;	crov002	Eukaryota; Amoebozoa; Mycetozoa; Dictyosteliida; Polysphondylium;	lcl|UniRef100_D3AX89
Viruses; dsDNA viruses, no RNA stage; Mimiviridae;	crov148	Eukaryota; Heterolobosea; Schizopyrenida; Vahlkampfiidae; Naegleria;	lcl|UniRef100_D2V5I7
Viruses; dsDNA viruses, no RNA stage; Mimiviridae;	crov424	Eukaryota; Heterolobosea; Schizopyrenida; Vahlkampfiidae; Naegleria;	lcl|UniRef100_D2W103
Viruses; dsDNA viruses, no RNA stage; Mimiviridae;	UniRef100_Q5UQR2	Eukaryota; stramenopiles; **Oomycetes**; Hyaloperonospora_arabidopsidis	lcl|Hyalo_arabi_gi|373903105|gb
Viruses; dsDNA viruses, no RNA stage; Mimiviridae;	UniRef100_Q5UQX5	Eukaryota; stramenopiles; **Oomycetes**; Aphanomyces_euteiches	lcl|Apha_eut_NX0AMYA13YB12CM1.SCF_2
Viruses; dsDNA viruses, no RNA stage; Mimiviridae;	UniRef100_Q5UR69	Eukaryota; stramenopiles; **Oomycetes**; Peronosporales; Phytophthora;	lcl|UniRef100_D0NN40
Viruses; dsDNA viruses, no RNA stage; Mimiviridae;	UniRef100_F8V6J7	Eukaryota; stramenopiles; **Oomycetes**; Phytophthora_ramorum	lcl|Phyto_ramo_Pr_85700T0
Viruses; dsDNA viruses, no RNA stage; Mimiviridae;	crov120	Eukaryota; stramenopiles; **Oomycetes**; Pythium_ultimum	lcl|Pythi_ulti_PYU1_T000876
Viruses; dsDNA viruses, no RNA stage; Mimiviridae;	UniRef100_Q5UP50	Eukaryota; stramenopiles; **Oomycetes**; Saprolegnia_parasitica	lcl|Sapro_parasi_SPRG_18395T0
Viruses; dsDNA viruses, no RNA stage; Mimiviridae;	UniRef100_Q5UQ35	Eukaryota; stramenopiles; **Oomycetes**; Saprolegnia_parasitica	lcl|Sapro_parasi_SPRG_19367T0
Viruses; dsDNA viruses, no RNA stage; Mimiviridae;	crov114	Eukaryota; stramenopiles; PX clade; Phaeophyceae; Ectocarpales; Ectocarpaceae; Ectocarpus;	lcl|UniRef100_D7FNE9
Viruses; dsDNA viruses, no RNA stage; Mimiviridae;	crov417	Eukaryota; stramenopiles; PX clade; Phaeophyceae; Ectocarpales; Ectocarpaceae; Ectocarpus;	lcl|UniRef100_D7FXM5
Viruses; dsDNA viruses, no RNA stage; Mimiviridae;	crov527	Eukaryota; stramenopiles; PX clade; Phaeophyceae; Ectocarpales; Ectocarpaceae; Ectocarpus;	lcl|UniRef100_D7G5P9
Viruses; dsDNA viruses, no RNA stage; Mimiviridae;	crov530	Eukaryota; stramenopiles; PX clade; Phaeophyceae; Ectocarpales; Ectocarpaceae; Ectocarpus;	lcl|UniRef100_D8LD35
