# Supplementary figures and images for: Exploring nucleo-cytoplasmic large DNA viruses in Tara Oceans microbial metagenomes
Source: ISME J. 2013 Apr 11;7(9):1678–95. doi: 10.1038/ismej.2013.59 (PMC3749498; doi:10.1038/ismej.2013.59)

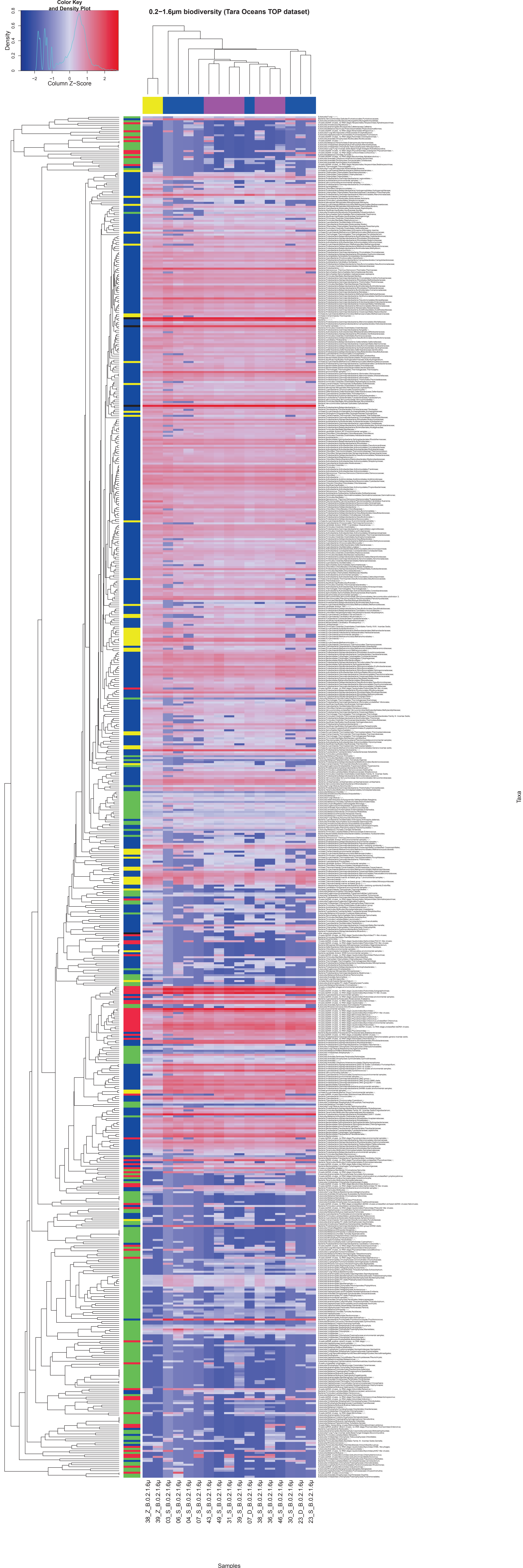

Supplement: Supplementary File 2 [file ismej201359x8.pdf]
